# Impact of immune checkpoint inhibitors as neoadjuvant/perioperative therapy for locally advanced resectable gastric/gastroesophageal junction adenocarcinoma: a systematic review and meta-analysis

**DOI:** 10.3389/fimmu.2026.1909466

**Published:** 2026-09-25

**Authors:** Zhengyi Chen, Xin Liu, Hanling Sun, Nini Cao, Junlin Zhou, Zihan Chen, Yang Song, Jun Yao

**Affiliations:** 1 Department of Gastroenterology, Shenzhen People’s Hospital, The Second Clinical Medical College, Jinan University, Shenzhen, China; 2 The Fourth School of Clinical Medicine, Guangzhou University of Chinese Medicine, Guangzhou, China; 3 Shenzhen College of Clinical Medicine, Guangzhou University of Chinese Medicine, Shenzhen, China; 4 Department of Gastroenterology, Shenzhen People’s Hospital (the Second Clinical Medical College, Jinan University, The First Affiliated Hospital, Southern University of Science and Technology), Shenzhen, China

**Keywords:** gastric/gastroesophageal junction adenocarcinoma, immune checkpoint inhibitors, meta-analysis, neoadjuvant, perioperative, systematic review

## Abstract

**Background:**

Immune checkpoint inhibitor (ICI)-based neoadjuvant/perioperative strategies are expanding for locally advanced resectable gastric/gastroesophageal junction (G/GEJ) adenocarcinoma, but the evidence base differs substantially across strategies, and improved pathological response has not consistently translated into survival benefit. We aimed to synthesize strategy-specific comparative and prospective single-arm evidence.

**Methods:**

PubMed, Web of Science, Embase, the Cochrane Library, and major oncology conference proceedings were searched from inception to August 3, 2026. RCTs and prospective single-arm trials evaluating ICI-containing treatment initiated before surgery were included. Five strategies were evaluated: ICI plus chemotherapy (ICI+CT), ICI plus chemotherapy and anti-angiogenic therapy (ICI+CT+A), ICI plus chemoradiotherapy (ICI+CRT), ICI plus chemotherapy and anti-HER2 therapy (ICI+CT+H), and dual-ICI therapy (ICI+ICI). Random-effects comparative meta-analyses were conducted separately by strategy, while single-arm analyses provided descriptive absolute estimates.

**Results:**

A total of 74 trials, including 16 RCTs and 58 prospective single-arm trials, were included. In randomized comparisons, ICI+CT was associated with improved pCR (RR, 3.01; 95% CI, 2.19-4.15) and MPR (RR, 1.59; 95% CI, 1.36-1.85) and, in perioperative trials, OS (HR, 0.81; 95% CI, 0.70-0.93) and EFS (HR, 0.74; 95% CI, 0.66-0.83) compared with chemotherapy. ICI+CT+A was associated with improved pCR (RR, 3.43; 95% CI, 1.85-6.37) and MPR (RR, 1.40; 95% CI, 1.12-1.76), but survival evidence was limited to one phase II RCT reporting OS. Comparative evidence for ICI+CRT was limited to one small RCT. Evidence for ICI+CT+H and ICI+ICI was derived mainly from small biomarker-selected noncomparative studies.

**Conclusions:**

Evidence differed substantially across the five strategies. ICI+CT was associated with improved pCR and MPR and, in perioperative RCTs, OS and EFS; it had the largest and most mature randomized evidence base. ICI+CT+A was associated with improved pCR and MPR, whereas comparative evidence for ICI+CRT, ICI+CT+H, and ICI+ICI remained limited. Further comparative trials are required, particularly for safety and long-term survival.

**Systematic review registration:**

https://www.crd.york.ac.uk/PROSPERO/view/CRD420251271789, identifier CRD420251271789.

## 1 Introduction

Gastric cancer is the fifth most common cancer worldwide and the fifth leading cause of cancer-related deaths (1). Phase III clinical trials such as CheckMate-649 (2) and KEYNOTE-859 (3) have demonstrated that ICIs plus chemotherapy significantly improve overall survival (OS) in the first-line treatment of advanced gastric cancer, prompting its exploration in the neoadjuvant/perioperative setting. Theoretically, the presence of the primary tumor during the neoadjuvant phase may provide abundant tumor-associated antigens and promote stronger *in situ* immune activation (4). Based on this, multiple clinically distinct ICI-based neoadjuvant/perioperative strategies have been investigated. In this review, these strategies were classified according to their preoperative treatment components as ICI plus chemotherapy (ICI+CT), ICI plus chemotherapy and anti-angiogenic therapy (ICI+CT+A), ICI plus chemoradiotherapy (ICI+CRT), ICI plus chemotherapy and anti-HER2 therapy (ICI+CT+H), and dual-ICI therapy (ICI+ICI).

Although ICI-based neoadjuvant/perioperative strategies have shown encouraging pathological responses, their clinical interpretation remains challenging. Some strategies have been evaluated in randomized controlled trials, while others have been explored primarily in single-arm studies or biomarker-selected cohorts. Furthermore, improvements in pathological response do not always translate into survival benefits, as illustrated by the inconsistent results of the KEYNOTE-585 (5) and MATTERHORN (6) trials. This discrepancy indicates that pathological response cannot be directly equated with durable clinical benefit and underscores the need to consider the strength, design, and maturity of the supporting evidence for each strategy.

Previous meta-analyses have generally focused on narrower questions, such as ICI plus chemotherapy versus chemotherapy alone. However, the field has rapidly expanded beyond a single treatment model. Anti-angiogenic combinations, HER2-directed chemoimmunotherapy, chemoradiotherapy-based intensification, and chemotherapy-free dual ICI strategies in deficient mismatch repair tumors are now being explored. Therefore, the key challenge is to characterize the evidence for each strategy separately, considering study design, biomarker selection, treatment intensity, toxicity, perioperative feasibility, and survival maturity.

This systematic review and meta-analysis evaluated ICI-based neoadjuvant/perioperative treatment for locally advanced resectable gastric/gastroesophageal junction (G/GEJ) adenocarcinoma. We synthesized comparative randomized evidence separately by treatment strategy and summarized prospective single-arm evidence as strategy-specific descriptive estimates. Pathological, survival, surgical, radiological, and safety outcomes were assessed.

## 2 Methods

### 2.1 Study protocol

This systematic review and meta-analysis followed the PRISMA reporting guideline. The study protocol was registered with PROSPERO (CRD420251271789). Differences between the registered protocol and the final review are reported in **Supplementary eTable 1**.

### 2.2 Eligibility criteria

Inclusion criteria were defined according to the PICOS framework: (i) Population: patients with histologically or cytologically confirmed, locally advanced resectable G/GEJ adenocarcinoma (clinical stage ≥cT2 and/or cN+ and no metastasis); GEJ adenocarcinomas were included according to the definition used in the original study, including reported Siewert subtypes, whereas studies restricted to esophageal adenocarcinoma or those in which eligible G/GEJ adenocarcinoma data could not be separately extracted were excluded; (ii) Intervention: ICI-based neoadjuvant/perioperative treatment initiated before surgery. Strategies were classified according to their preoperative treatment components as ICI plus chemotherapy (ICI+CT), ICI plus chemotherapy and anti-angiogenic therapy (ICI+CT+A), ICI plus chemoradiotherapy (ICI+CRT), ICI plus chemotherapy and anti-HER2 therapy (ICI+CT+H), or dual-ICI therapy (ICI+ICI); (iii) Comparison: the corresponding ICI-free chemotherapy-alone control strategy for pairwise meta-analyses; (iv) Outcomes: pCR, major pathologic response (MPR), OS, event-free survival (EFS), progression-free survival (PFS), surgical resection rate, R0 resection rate, disease control rate (DCR), objective response rate (ORR), treatment-related adverse events (TRAEs), immune-related adverse events (irAEs), and postoperative complications; (v) Study design: RCTs and prospective single-arm clinical trials. Conference abstracts were eligible if they met the predefined eligibility criteria and provided sufficient extractable outcome data. Retrospective studies, studies involving non-specified regimens (e.g., ICI monotherapy), and studies with non-extractable data were excluded.

### 2.3 Information sources and search strategy

A systematic search was conducted in PubMed, Web of Science, Embase, and the Cochrane Library, as well as conference abstracts from American Society of Clinical Oncology (ASCO), European Society for Medical Oncology (ESMO), and American Association for Cancer Research (AACR). The search covered the period from database inception to August 3, 2026, with no language or country restrictions. The detailed search strategy is provided in **Supplementary eTables 2–5**.

### 2.4 Selection process and data collection

After removing duplicates, two investigators (Z.Y.C. and X.L.) independently screened the titles and abstracts of all retrieved records against the predefined eligibility criteria. Full texts of the remaining records were then independently assessed for eligibility by the same two investigators. Any disagreement at either stage was resolved by consultation with a third investigator (H.L.S.). When multiple reports described the same study or overlapping cohort, they were treated as a single study, with the most complete and/or most recent report preferentially used for each outcome. Conference abstracts were not counted separately when a corresponding full-text report was available.

Data were independently extracted by two investigators (Z.Y.C. and X.L.), with disagreements resolved by consultation with a third investigator (H.L.S.). The intention-to-treat (ITT) population was used as the primary analysis population for efficacy endpoints (pCR, MPR, R0 resection rate, ORR, and DCR). Corresponding data based on the per-protocol (PP) population were additionally extracted or calculated for sensitivity analyses. TRAEs and irAEs were synthesized separately according to severity (any grade and grade ≥3) and the source-reported neoadjuvant or perioperative safety period, using the reported safety or treated population. Study-level observation windows, analysis denominators, CTCAE versions, and attribution criteria were also extracted. See **Supplementary eMethods** for details.

### 2.5 Quality assessment and certainty of evidence

Two reviewers (Z.Y.C. and X.L.) independently assessed the risk of bias of RCTs using the revised Cochrane risk-of-bias ROB 2.0 tool and the methodological quality of single-arm studies using the Methodological Index for Non-Randomized Studies (MINORS) checklist. The quality of evidence was evaluated using the GRADE tool. Any disagreements were resolved by a third reviewer (H.L.S.).

### 2.6 Statistical analysis

Analyses were performed using the meta package in R software (version 4.2.1). For pairwise meta-analyses, treatment effects were pooled separately by treatment strategy using risk ratios (RRs) for binary outcomes and hazard ratios (HRs) for time-to-event outcomes, with restricted maximum likelihood (REML) estimation for random-effects models. For single-arm meta-analyses, pooled proportions and 95% CIs were calculated separately by treatment strategy, and, where applicable, regimen subtype using inverse-variance random-effects models with logit transformation. These estimates were interpreted descriptively and were not compared across treatment strategies or regimen subtypes.

Across all forest plots in the main article and Supplement, k denotes the number of unique underlying trials contributing to each pooled estimate, and N denotes the corresponding outcome-specific analysis denominator; detailed outcome-specific definitions of N are provided in Supplementary eMethods. Multiple reports from the same trial were counted once for k, and no participant was counted more than once within any single outcome analysis.

Leave-one-out sensitivity analyses were conducted for pCR and MPR. Where sufficient data were available, additional sensitivity meta-analyses were performed only when at least 2 eligible studies remained. In pairwise meta-analyses of ICI+CT versus chemotherapy, sensitivity analyses excluding conference-abstract-derived outcome data and RCTs at high risk of bias were conducted for pCR, MPR, surgical resection rate, and R0 resection rate. Sensitivity analyses excluding trials enrolling biomarker-enriched populations were conducted for pCR, MPR, and EFS. No separate OS sensitivity meta-analysis was conducted because all RCTs contributing OS data were full-text reports assessed as having low risk of bias, and none met the biomarker-enrichment exclusion criterion. No separate sensitivity estimate was generated for ICI+CT+A versus chemotherapy because all contributing outcome data were derived from full-text reports, both RCTs were assessed as having low risk of bias, and neither trial enrolled a biomarker-enriched population. For single-arm meta-analyses, sensitivity analyses excluding conference-abstract-derived outcome data were conducted for pCR and MPR across treatment strategies and regimen subtypes when applicable. Additional sensitivity analyses excluding biomarker-enriched cohorts were conducted for pCR and MPR in the ICI+CT category. Per-protocol analyses were conducted for pCR, MPR, DCR, ORR, and R0 resection rate when sufficient data were available, with intention-to-treat analyses considered primary.

Exploratory subgroup analyses were performed in ICI+CT versus chemotherapy when sufficient data were available for pCR, MPR, OS, and EFS. Factors evaluated included PD-L1 expression, MMR status, Lauren classification, T stage, primary tumor location, ECOG performance status, sex, age, and region. Subgroup analyses were not systematically performed for single-arm meta-analyses because subgroup data were inconsistently reported and no common comparator was available.

Publication-bias assessments were limited to strategy-specific pairwise meta-analyses with at least 10 included comparisons. Funnel plots were inspected visually, and Harbord’s test was used for binary outcomes. Trim-and-fill analyses were undertaken only as exploratory sensitivity analyses when asymmetry was suspected.

## 3 Results

### 3.1 Study characteristics

The literature search yielded 2,807 records, and a total of 74 trials (involving 82 literature reports) were ultimately included (**Figure 1**), comprising 16 RCTs (23 literature reports) and 58 prospective single-arm trials (59 literature reports). A detailed report-to-trial linkage mapping, including patient overlap declarations and handling rules for multiple reports from the same trial, is provided in **Supplementary eTable 6**.

**Figure 1:**
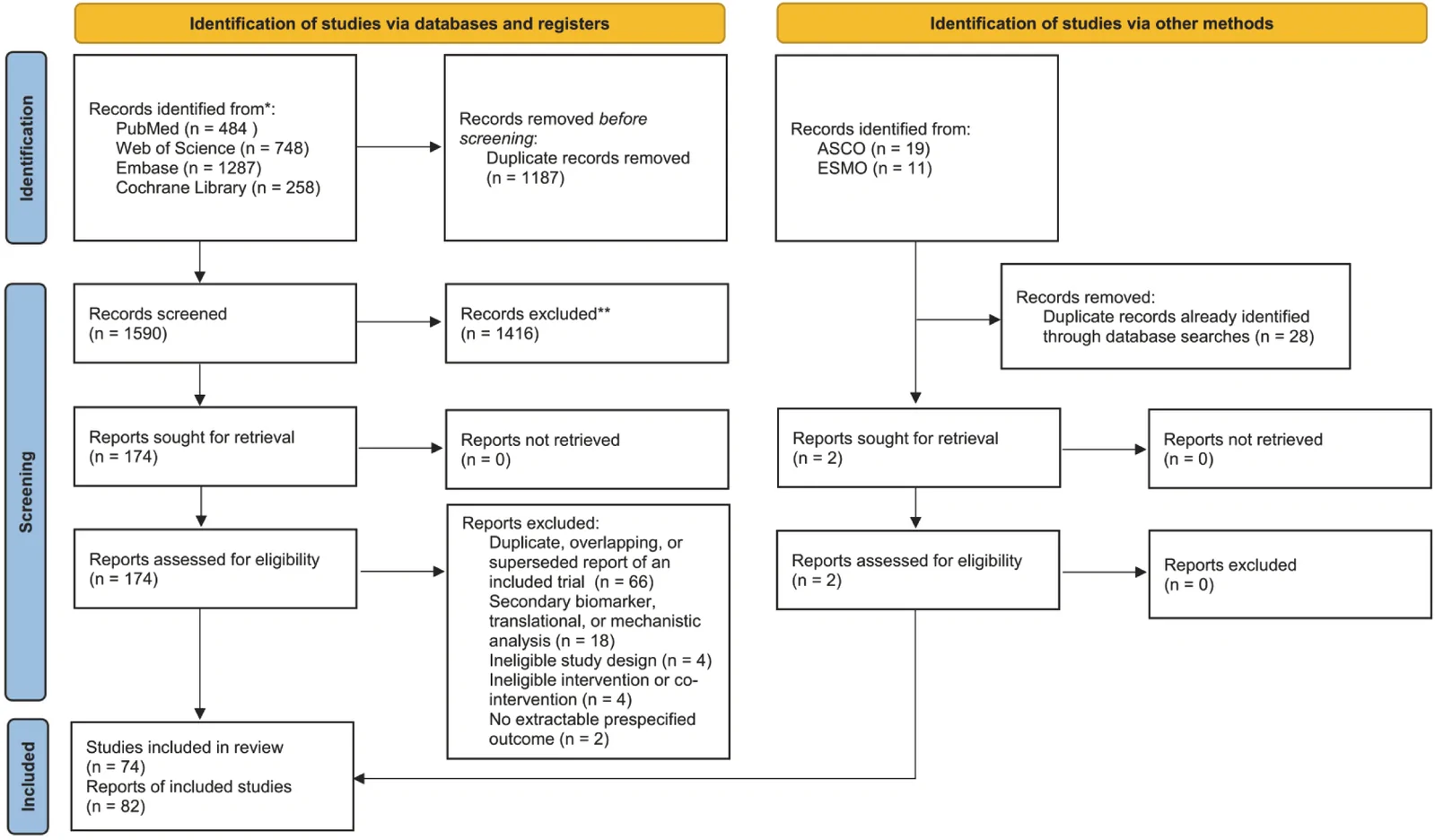
Study flowchart.

Among the 16 RCTs, 10 compared ICI+CT with chemotherapy (5–20). In Mountain-02, patients were stratified by prespecified tsMHC-II status before randomization; for the pairwise meta-analysis, the tsMHC-II-positive and tsMHC-II-negative strata were combined within each treatment group (A+C vs. B+D) to obtain the overall randomized comparison. In KEYNOTE-585, the main and FLOT cohorts were analyzed separately when cohort-specific outcome data were available; when only combined results were reported, the combined estimate was used without simultaneous inclusion of either cohort-specific estimate. The 2 cohorts were counted as 1 underlying trial for study-level accounting. 2 compared ICI+CT+A with chemotherapy (21–23); 1 was a three-arm trial (ICI+CRT vs. CRT vs. chemotherapy), from which only the ICI+CRT and chemotherapy arms were extracted according to the inclusion criteria (24); and 1 compared trastuzumab-based ICI+CT+H with CT+H, from which only the trastuzumab-based ICI+CT+H arm was included in the single-arm meta-analysis (25). In addition, 1 trial compared ICI plus different chemotherapy intervals, and the results of the two arms were reported together in the original article, so the combined results were included in the single-arm meta-analysis (26); another trial compared ICI plus different chemotherapy regimens, and the two arms were separately included in the single-arm meta-analysis (27).

Among the RCTs, ASTRUM-006 enrolled a biomarker-enriched population restricted to patients with PD-L1 CPS ≥5 (8), whereas Peng 2025 enrolled patients with HER2-positive disease (25). The remaining RCTs did not enroll biomarker-enriched populations.

Among the 58 single-arm trials, there were 35 ICI+CT trials (28–63), 6 ICI+CT+A trials (64–69), 8 ICI+CRT trials (70–77), 7 ICI+CT+H trials (78–84), and 2 ICI+ICI trials (85, 86). The 35 ICI+CT trials contributed 36 non-overlapping treatment cohorts, because NCT04744649 included separate EBV-associated and dMMR/MSI-H cohorts; these were counted as one trial for study-level accounting and as two cohorts in the single-arm analyses.

Among the single-arm trials, within the ICI+CT category, three cohorts were biomarker-enriched, comprising dMMR/MSI-H, EBV-positive, or PD-L1-selected populations (29, 33, 47), whereas the remaining cohorts were not biomarker-enriched. No ICI+CRT study enrolled a biomarker-enriched population; this category comprised 7 single-ICI+CRT cohorts and one dual-ICI-containing multimodality cohort, which was reported separately. All ICI+CT+H cohorts were HER2-selected, comprising 3 disitamab vedotin-based and 4 trastuzumab-based cohorts, which were analyzed separately. Trastuzumab-based cohorts required HER2 positivity defined as IHC 3+ or IHC 2+ with ISH positivity, whereas disitamab vedotin-based cohorts enrolled patients with HER2-overexpressing tumors defined as IHC 2+ or 3 +. Both ICI+ICI studies were restricted to patients with dMMR/MSI-H tumors.

In summary, 13 RCTs involving 4,088 patients were included in pairwise meta-analysis (**Table 1**). Eligible ICI-containing treatment arms from all 16 RCTs, together with all 58 prospective single-arm trials, were included in the single-arm meta-analysis (**Supplementary eTable 7**), involving 4,128 patients. The sample sizes for the evaluated regimens in the single-arm analysis were as follows: ICI+CT (n=3,186), ICI+CT+A (n=405), ICI+CRT (n=280), ICI+CT+H (n=207), and ICI+ICI (n=50).

**Table 1:** Basic characteristics of the RCTs included in the pairwise meta-analysis.

Group	Study	Registration No.	Country	Phase	Sample size	Age	Proportion of males (%)	Proportion of cT4 (%)	Neoadjuvant therapy	Adjuvant therapy
ICI+CT vs. chemotherapy	Xu 2026 (7)	NCT06374901	China	II	68	/	76.5	82.4	Tislelizumab+Chemo (SOX/XELOX)	Tislelizumab+Chemo (SOX/XELOX)
					68	/	78	79.4	Chemo (SOX/XELOX)	Chemo (SOX/XELOX)
ICI+CT vs. chemotherapy	Shen 2026 (8)	NCT04139135	China	III	292	61.5 (55-66)	80.1	60.6	Serplulimab+Chemo (SOX)	Serplulimab
					296	60 (55-65)	77.7	60.8	Chemo (SOX)	Chemo (SOX)
ICI+CT vs. chemotherapy	Janjigian 2023 (9), Janjigian 2025 (10), Tabernero 2025 (6)	NCT04592913	United States	III	474	62 (26-84)	68.8	24.7	Durvalumab+Chemo (FLOT)	Durvalumab+Chemo (FLOT)
					474	63 (28-83)	75.1	24.7	Placebo+Chemo (FLOT)	Placebo+Chemo (FLOT)
ICI+CT vs. chemotherapy	Shitara 2024 (11), Shitara 2025 (5), Shitara 2026 (12)	NCT03221426^a^	Japan	III	402	64 (56-70)	72	41	Pembrolizumab+Chemo (XP/FP)	Pembrolizumab+Chemo (XP/FP)
					402	63 (55-69)	71	41	Placebo+Chemo (XP/FP)	Placebo+Chemo (XP/FP)
ICI+CT vs. chemotherapy	Al-Batran 2024 (13), Shitara 2024 (11), Shitara 2025 (5)	NCT03221426^a^	Japan	III	100	61.5 (54.5-69)	78	/	Pembrolizumab+Chemo (FLOT)	Pembrolizumab+Chemo (FLOT)
					103	61 (52-68)	69	/	Placebo+Chemo (FLOT)	Placebo+Chemo (FLOT)
ICI+CT vs. chemotherapy	Lorenzen 2024 (14)	NCT03421288	Germany	II/III	146	61 (29-79)	80	5	Atezolizumab+Chemo (FLOT)	Atezolizumab+Chemo (FLOT)
					149	62 (23-80)	68	11	Chemo (FLOT)	Chemo (FLOT)
ICI+CT vs. chemotherapy	Yuan 2024 (15), Nie 2026 (16)	NCT04250948	China	II	54	58 (48–67)	64.8	61.1	Toripalimab+Chemo (SOX/XELOX)	Toripalimab+Chemo (SOX/XELOX)
					54	62 (54–68)	64.8	68.5	Chemo (SOX/XELOX)	Chemo (SOX/XELOX)
ICI+CT vs. chemotherapy	Li 2026 (17)	NCT06576921	China	II (Abstract)	23	/	/	/	Serplulimab+Chemo (SOX+nab-paclitaxel)	Serplulimab+Chemo (SOX+nab-paclitaxel)
					28	/	/	/	Chemo (SOX+nab-paclitaxel)	Chemo (SOX+nab-paclitaxel)
ICI+CT vs. chemotherapy	Yang 2025 (18)	NCT06192186	China	II (Abstract)	55	/	/	/	Adebrelimab+Chemo (SOX)	Determined according to postoperative pathological findings
					55	/	/	/	Chemo (SOX)	Determined according to postoperative pathological findings
ICI+CT vs. chemotherapy	Ding 2025 (19)	NCT04982939	China	II (Abstract)	131	/	/	/	Sintilimab+Chemo (SOX)	Sintilimab+Chemo (SOX)
					133	/	/	/	Chemo (SOX)	Chemo (SOX)
ICI+CT vs. chemotherapy	Min 2022 (20)	ChiCTR2000030610	China	II (Abstract)	33	/	/	/	Camrelizumab+Chemo (FLOT)	Not reported
					30	/	/	/	Chemo (FLOT)	Not reported
ICI+CT+A vs. chemotherapy	Li 2025 (21)	NCT04208347	China	III	180	63 (28–75)	83.9	66.1	Camrelizumab+Apatinib+Chemo (SOX)	Camrelizumab+Apatinib+SOX (first 3 cycles) → Camrelizumab+Apatinib (next 3 cycles)
					180	63 (34–75)	80.6	67.2	Chemo (SOX)	Chemo (SOX)
ICI+CT+A vs. chemotherapy	Lin 2024 (22), Lin 2025 (23)	NCT04195828	China	II	51	63 (57–68)	82.4	90.2	Camrelizumab+Apatinib+Chemo (SAP)	Camrelizumab+Apatinib+Chemo (SAP)
					53	63 (56–68)	66.0	84.9	Chemo (SAP)	Chemo (SAP)
ICI+CRT vs. chemotherapy	Wei 2026 (24)	NCT05687357	China	IIb (Abstract)	35	/	/	/	Tislelizumab+Radiotherapy+Chemo (SOX/SAP)	Chemo (S-1)
					19	/	/	/	Chemo (SOX/SAP)	Chemo (S-1)

FLOT, Fluorouracil, Leucovorin, Oxaliplatin and Docetaxel; FP, Fluorouracil and Cisplatin; XP: Capecitabine and Cisplatin; SOX, S-1 and Oxaliplatin; CapeOX (XELOX), Capecitabine and Oxaliplatin; SAP, S-1 and Nab-paclitaxel.

^a^KEYNOTE-585 main and FLOT cohorts were counted as 1 underlying trial. Cohort-specific data were analyzed separately when available; when only combined data were reported, the combined estimate was used without simultaneous inclusion of either cohort-specific estimate.

Among the 16 included RCTs, 8 were assessed as having a low overall risk of bias, 2 as having some concerns, and 6 as having a high overall risk of bias (**Supplementary eFigure 1**). Among the 9 RCTs published as full-text articles (5, 7, 8, 10, 14, 15, 21, 22, 25), all were assessed as having a low overall risk of bias except Peng 2025 (25), which was rated as high risk because of missing outcome data. Among the 7 RCTs reported only as conference abstracts, 2 studies were assessed as having some concerns: Li 2026 (17) because of concerns regarding the randomization process and Gao 2024 (26) because of concerns regarding the selection of the reported result. The remaining 5 RCTs (18–20, 24, 27) were assessed as having a high overall risk of bias, primarily because of missing outcome data. Among the included single-arm trials, most were assessed as high quality (≥12 points) (**Supplementary eTable 8**).

### 3.2 Pathological response

#### 3.2.1 pCR

In pairwise meta-analyses, ICI+CT (RR, 3.01; 95% CI, 2.19-4.15; I² = 42.3%) and ICI+CT+A (RR, 3.43; 95% CI, 1.85-6.37; I² = 0%) were each associated with higher pCR than chemotherapy. The single ICI+CRT randomized trial reported an RR of 10.44 (95% CI, 0.64-169.86) (**Figure 2A**).

**Figure 2:**
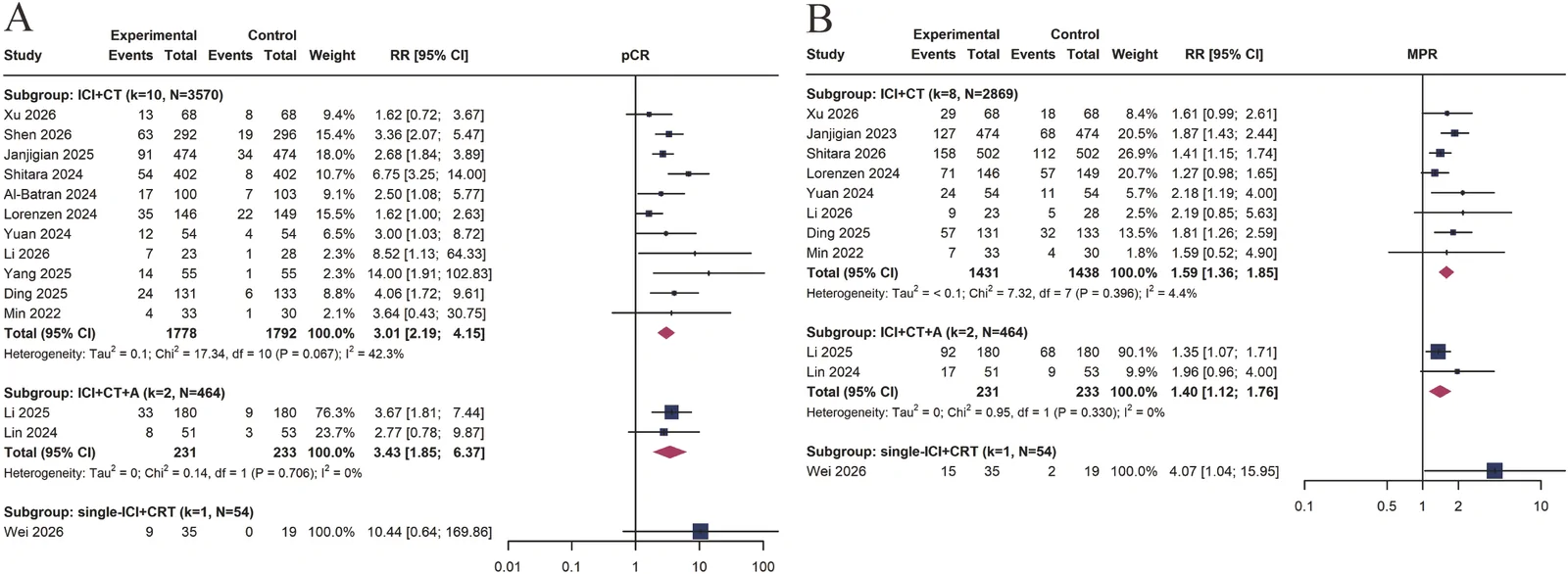
Pooled risk ratios of pCR **(A)** and MPR **(B)** across randomized clinical trials.

In single-arm meta-analyses, the pooled pCR rates were 21% (95% CI, 19%-23%; I² = 41.7%) for ICI+CT, 19% (95% CI, 15%-23%; I² = 0%) for ICI+CT+A, and 29% (95% CI, 23%-35%; I² = 8.2%) for single-ICI+CRT. The dual-ICI-containing multimodality study reported a pCR rate of 30% (95% CI, 15%-49%) and was not included in the single-ICI+CRT pooled estimate. Within the ICI+CT+H category, the pooled pCR rates were 26% (95% CI, 18%-37%; I² = 6.6%) for disitamab vedotin-based regimens and 39% (95% CI, 29%-50%; I² = 32.5%) for trastuzumab-based regimens. The pooled pCR rate was 52% (95% CI, 38%-65%; I² = 0%) for ICI+ICI (**Supplementary eFigure 2**). These estimates were descriptive and were not compared across treatment strategies or regimen subtypes.

#### 3.2.2 MPR

In pairwise meta-analyses, ICI+CT (RR, 1.59; 95% CI, 1.36-1.85; I² = 4.4%) and ICI+CT+A (RR, 1.40; 95% CI, 1.12-1.76; I² = 0%) were each associated with higher MPR than chemotherapy. The single ICI+CRT randomized trial reported an RR of 4.07 (95% CI, 1.04-15.95) (**Figure 2B**).

In single-arm meta-analyses, the pooled MPR rates were 39% (95% CI, 35%-44%; I² = 75.5%) for ICI+CT, 38% (95% CI, 29%-48%; I² = 67.0%) for ICI+CT+A, and 52% (95% CI, 37%-67%; I² = 74.3%) for single-ICI+CRT. The dual-ICI-containing multimodality study reported an MPR rate of 47% (95% CI, 28%-66%) and was not included in the single-ICI+CRT pooled estimate. Within the ICI+CT+H category, the pooled MPR rates were 44% (95% CI, 27%-63%; I² = 65.5%) for disitamab vedotin-based regimens and 54% (95% CI, 45%-63%; I² = 0%) for trastuzumab-based regimens. The pooled MPR rate was 64% (95% CI, 50%-76%; I² = 0%) for ICI+ICI (**Supplementary eFigure 3**). These estimates were descriptive and were not compared across treatment strategies or regimen subtypes.

#### 3.2.3 Exploratory subgroup analyses

Exploratory subgroup analyses were conducted only for ICI+CT versus chemotherapy because the number of ICI+CT+A trials was insufficient for reliable subgroup analyses. For pCR (**Supplementary eFigure 4**), point estimates favored ICI+CT over chemotherapy across all evaluated subgroups. The pooled effect did not reach nominal statistical significance in the PD-L1 CPS <1 subgroup (RR, 1.65; 95% CI, 0.80-3.41). No statistically significant subgroup difference was detected for any evaluated factor.

For MPR (**Supplementary eFigure 5**), subgroup analyses were feasible only for PD-L1 expression and MMR status. Point estimates favored ICI+CT over chemotherapy across the available subgroups, although nominal statistical significance was not consistently observed within individual subgroups. No statistically significant subgroup difference was detected.

### 3.3 Survival outcomes

All RCTs contributing to the pooled comparative OS and EFS analyses used perioperative designs. Long-term follow-up data for 3-year OS were reported in 2 phase III and 2 phase II RCTs, while 3-year EFS data were available from 3 phase III and 1 phase II RCTs. ICI+CT was associated with improved OS (HR, 0.81; 95% CI, 0.70-0.93; I² = 29.8%) and EFS (HR, 0.74; 95% CI, 0.66-0.83; I² = 0%) compared with chemotherapy (**Figure 3**). The single ICI+CT+A trial reporting survival yielded an OS HR of 0.99 (95% CI, 0.48-2.03) and PFS HR of 0.51 (95% CI, 0.26-0.998). No comparative survival data were available for the remaining strategies. In an exploratory analysis of patients achieving MPR, the pooled EFS HR was 0.47 (95% CI, 0.26-0.86; I²=48.5%) across two ICI+CT RCTs (**Supplementary eFigure 6**).

**Figure 3:**
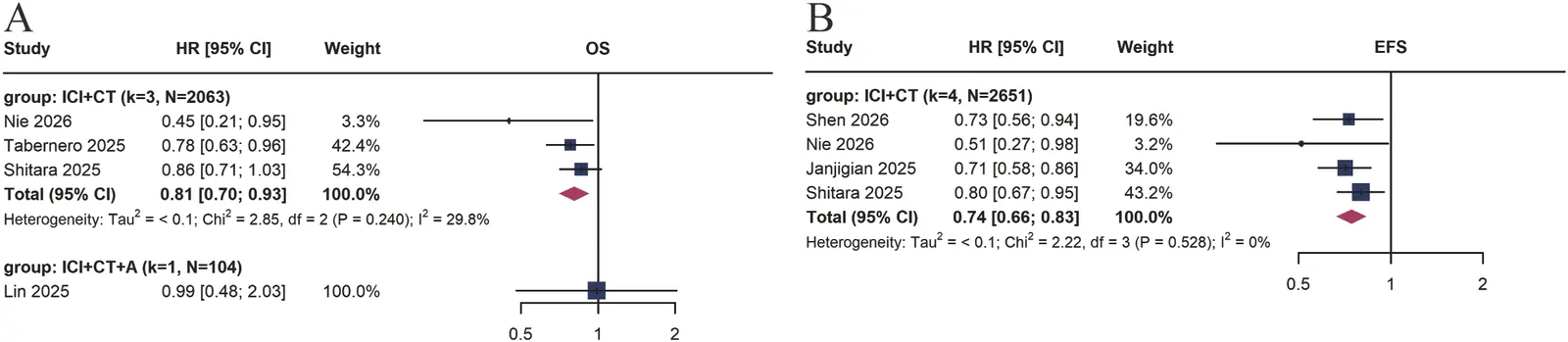
Pooled hazard ratios of OS **(A)** and EFS **(B)** across randomized clinical trials.

The majority of single-arm trials have not yet reported follow-up data for 3-year OS, EFS and PFS. The 1-, 2-, and 3-year rates for OS, EFS and PFS are shown in **Supplementary eFigures 7–9**.

In exploratory subgroup analyses for OS and EFS (**Supplementary eFigures 10, 11**), point estimates generally favored ICI+CT over chemotherapy across all evaluated clinical and biomarker-defined subgroups. Statistical significance was not consistently observed across individual subgroups, and no statistically significant interaction was identified for any subgroup factor.

### 3.4 Surgical outcomes

In pairwise meta-analyses, the RR for surgical resection was 1.02 (95% CI, 1.00-1.05; I²=0%) for ICI+CT versus chemotherapy and 1.01 (95% CI, 0.95-1.07; I²=0%) for ICI+CT+A versus chemotherapy. The corresponding R0 resection RRs were 1.04 (95% CI, 1.01-1.07; I²=12.6%) and 1.08 (95% CI, 0.98-1.20; I²=33.3%), respectively (**Supplementary eFigure 12**). The single ICI+CRT randomized trial reported an RR of 0.96 (95% CI, 0.65–1.42) for surgical resection; comparative R0 resection data were unavailable.

Strategy-specific single-arm estimates of surgical and R0 resection rates are shown in **Supplementary eFigures 13, 14** and were interpreted descriptively only, without comparisons across strategies or ICI+CT+H regimen subtypes because of their noncomparative design and clinical heterogeneity.

### 3.5 Radiologic response

Radiologic response was assessed according to RECIST version 1.1 and was summarized only in single-arm analyses (**Supplementary eFigure 15**). For ICI+CT, the pooled DCR was 84% (95% CI, 67%-93%) and the pooled ORR was 55% (95% CI, 43%-67%). For ICI+CT+A, the corresponding pooled estimates were 96% (95% CI, 91%-98%) for DCR and 70% (95% CI, 50%-84%) for ORR. Within the ICI+CT+H category, pooled DCR and ORR estimates for trastuzumab-based regimens were 71% and 56%, respectively, whereas the single disitamab vedotin-based cohort reported a DCR of 88% and an ORR of 75%. The single ICI+ICI cohort reporting radiologic outcomes had a DCR of 88% and an ORR of 53%. No pooled radiologic-response estimate was available for ICI+CRT. Owing to heterogeneity and the noncomparative nature of these data, all single-arm estimates were interpreted descriptively and were not compared across strategies or regimen subtypes.

### 3.6 Safety analysis

Study-level safety observation windows, analysis denominators, CTCAE versions, and attribution criteria were extracted (**Supplementary eTables 9, 10**). Comparative evidence from RCTs was prioritized, with perioperative and neoadjuvant safety data analyzed separately according to the periods reported in each study.

For grade ≥3 TRAEs, ICI+CT did not differ significantly from chemotherapy during either the perioperative (RR, 1.01; 95% CI, 0.89-1.15; I² = 59.4%) or neoadjuvant period (RR, 1.04; 95% CI, 0.81-1.32; I² = 0%), whereas ICI+CT+A was associated with increased risk during both periods (perioperative: RR, 1.70; 95% CI, 1.29-2.25; I² = 18.6%; neoadjuvant: RR, 1.68; 95% CI, 1.09-2.58; I² = 36.9%) (**Figures 4A, B**). For grade ≥3 irAEs, ICI+CT was associated with an increased risk during the perioperative period (RR, 2.93; 95% CI, 1.19-7.23; I² = 25.9%) but not significantly during the neoadjuvant period (RR, 4.88; 95% CI, 0.84-28.30; I² = 0%) (**Figures 4C, D**). Comparative grade ≥3 irAE data for ICI+CT+A were insufficient for pooling. Any-grade comparative results are presented in **Supplementary eFigure 16**. Because safety assessment varied across trials with respect to observation windows, analysis populations, grading systems, and attribution criteria, the pooled RRs should be interpreted in the context of each study’s reported safety period and not as estimates based on a uniform exposure window. Single-arm safety estimates and postoperative complications are presented descriptively in **Supplementary eFigure 17–21** and were not compared across strategies.

**Figure 4:**
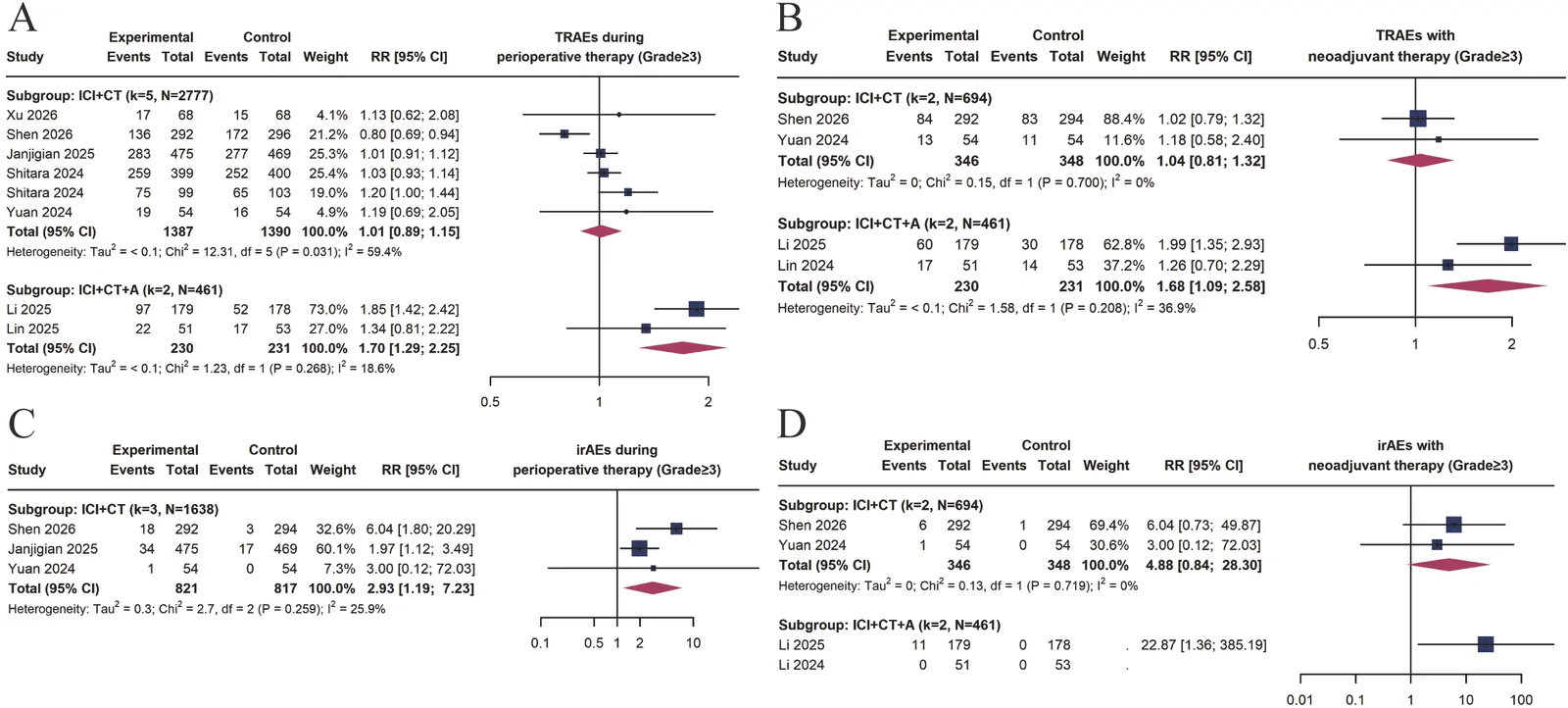
Pooled risk ratios of TRAEs (grade ≥3) during the perioperative period **(A)**, TRAEs (grade ≥3) during the neoadjuvant period **(B)**, irAEs (grade ≥3) during the perioperative period **(C)**, and irAEs (grade ≥3) during the neoadjuvant period **(D)** across randomized clinical trials.

### 3.7 Sensitivity analyses

In pairwise analyses, the direction and statistical significance of the pCR and MPR effects were unchanged after exclusion of conference-abstract-derived outcome data or RCTs at high risk of bias. Estimates for surgical and R0 resection remained similar in magnitude, although confidence intervals widened after these exclusions (**Supplementary eTable 11**). In single-arm analyses, sensitivity analyses showed generally stable pathological-response estimates for ICI+CT and ICI+CT+A, although some less precise analyses changed after exclusions, particularly MPR for single-ICI+CRT (**Supplementary eTable 12**). After exclusion of biomarker-enriched ICI+CT cohorts, pairwise estimates for pCR, MPR, and EFS, and single-arm estimates for pCR and MPR, remained materially unchanged (**Supplementary eTables 11, 12**). Leave-one-out analyses for pCR and MPR likewise showed no material change (**Supplementary eFigures 22–24**).

In PP analyses, the direction and statistical significance of the pairwise pCR and MPR effects were consistent with the ITT analyses, whereas the R0 resection advantage for ICI+CT was attenuated and no longer statistically significant (**Supplementary eTable 13**). Single-arm PP estimates were generally higher than the corresponding ITT estimates across pathological, surgical, and radiological outcomes, consistent with the more selected analysis populations included in PP analyses (**Supplementary eTable 14**).

### 3.8 Publication bias and level of evidence

Publication-bias assessment was undertaken only for pCR in the ICI+CT versus chemotherapy comparison, which was the only strategy-specific pairwise meta-analysis including 10 unique underlying trials. The funnel plot appeared symmetric, and Harbord’s test did not suggest small-study effects (P = 0.487) (**Supplementary eFigure 25**). Publication-bias assessments were not undertaken for the remaining outcomes or treatment strategies because fewer than 10 unique underlying trials contributed to each meta-analysis.

GRADE assessments showed high certainty for pCR, MPR, R0 resection rate, and perioperative OS and EFS with ICI+CT versus chemotherapy; moderate certainty for surgical resection rate. Comparative safety evidence was rated low to very low owing to heterogeneous observation windows and sparse events. Evidence for ICI+CT+A was moderate across all reported outcomes (**Supplementary eTable 15**).

## 4 Discussion

This systematic review and meta-analysis found that ICI+CT was associated with improved pCR and MPR and, in perioperative trials, OS and EFS compared with chemotherapy. ICI+CT+A was also associated with improved pCR and MPR, but comparative survival evidence remained limited. The other strategies lacked comparative survival data. Comparative findings should therefore be interpreted separately by treatment strategy rather than as a common ICI-based class effect. Currently, ICI+CT has the largest randomized evidence base and the most mature survival data, whereas evidence for ICI+CT+A, ICI+CRT, ICI+CT+H, and ICI+ICI remains more limited or heterogeneous and includes few randomized trials, prospective single-arm studies, and biomarker-selected cohorts.

Several overlapping meta-analyses have examined this topic (87–94), but their questions and evidence bases differ (**Supplementary eTable 16**). Early broad syntheses mainly pooled small single-arm studies across heterogeneous neoadjuvant ICI regimens, sometimes with historical chemotherapy cohorts, whereas later comparative reviews focused primarily on perioperative ICI+CT or phase III RCTs. Other reviews addressed narrower settings, including GEJ cancer or ICI+CRT, or combined randomized, prospective, and retrospective immunochemotherapy evidence. Building on these studies, our review includes only RCTs and prospective single-arm trials and organizes evidence according to five clinically distinct preoperative strategies. Randomized comparisons are synthesized separately by strategy, while noncomparative estimates are presented descriptively without cross-strategy ranking. This framework distinguishes findings supported by comparative survival data from hypothesis-generating evidence for anti-angiogenic, chemoradiotherapy, HER2-directed, and dual-ICI approaches.

Among the evaluated strategies, ICI+CT currently has the largest randomized evidence base and the most mature survival evidence. In analyses of randomized trials, ICI+CT was associated with higher pCR and MPR rates, as well as improved OS and EFS. These findings support ICI+CT as the current reference framework for evaluating other ICI-based neoadjuvant/perioperative strategies. Nevertheless, ICI+CT should not be viewed as a single uniform treatment model. The included trials differed in chemotherapy backbone, perioperative design, postoperative treatment exposure, patient characteristics, and biomarker distribution. A recent analysis of the MATTERHORN trial showed that patients who completed all adjuvant therapy had the highest event-free survival rate, and the durvalumab group demonstrated improved EFS compared with the chemotherapy group regardless of treatment completion status (95). Therefore, future research should clarify not only whether immunotherapy should be added, but also which chemotherapy backbone, treatment duration, and patient population provide the most favorable balance between efficacy and feasibility.

For ICI+CT+A, only one phase III trial (DRAGON IV/CAP 05) (21) and one phase II trial (Arise-FJ-G005) (22) have provided randomized controlled data confirming its advantage in pathological response. However, no trial directly compared ICI+CT+A with ICI+CT, and the pooled response rates from separate single-arm analyses cannot establish incremental benefit, equivalence, or inferiority between these strategies. Direct comparative evidence is therefore needed to determine whether adding anti-angiogenic therapy provides sufficient clinical benefit to justify the additional toxicity and perioperative complexity. Evidence for ICI+CRT was derived mainly from heterogeneous prospective single-arm studies, with only one small phase IIb randomized trial (24) providing comparative data. In the single-arm analyses, pooled estimates were restricted to single-ICI+CRT studies, whereas the dual-ICI-containing multimodality study was reported separately because of its distinct treatment composition. The comparative efficacy of ICI+CRT therefore remains uncertain.

Biomarker-selected strategies are an important direction for future research. PD-L1 expression has become a key screening indicator. ASTRUM-006, a phase III trial restricted to patients with PD-L1 CPS ≥5, evaluated perioperative serplulimab plus chemotherapy followed by postoperative serplulimab monotherapy. Both pCR and EFS were significantly improved, suggesting that perioperative chemoimmunotherapy can improve efficacy while reducing adjuvant toxicity through simultaneous optimization of biological characteristics and treatment intensity. In dMMR tumors, the pooled estimates for ICI+ICI were derived from two small phase 2, biomarker-selected cohorts and should be interpreted descriptively. The reported pathological responses were consistent with the biological sensitivity of dMMR/MSI-H gastric cancer to immune checkpoint blockade. These findings have motivated further investigation of chemotherapy-free treatment, treatment de-escalation, and organ-preservation strategies in selected patients. However, these approaches remain investigational because the available evidence is noncomparative, sample sizes are limited, and survival follow-up remains immature. It is noteworthy that the NEONIPIGA phase II trial (96) reported a 4-year overall survival (OS) rate of 84.1% in its long-term follow-up, suggesting that this strategy may provide a durable survival benefit. However, this still requires confirmation through prospective controlled trials. In HER2-selected tumors, trastuzumab-based and disitamab vedotin-based ICI+CT+H regimens were analyzed separately as clinically distinct approaches. Evidence for both subtypes was derived mainly from small noncomparative studies, and comparative survival data remain unavailable. Differences in HER2 eligibility criteria, chemotherapy backbones, and postoperative treatment further limit interpretation. Whether these biomarker-based selection strategies can improve long-term survival or alter surgical treatment decisions requires further prospective validation.

A key question in this field is whether pathological response can reliably predict long-term survival. Both KEYNOTE-585 (5) and MATTERHORN (10) significantly improved pCR and MPR, but only MATTERHORN (6) demonstrated significant OS and EFS improvements. This inconsistency may stem from differences in chemotherapy backbone, perioperative treatment design, postoperative treatment exposure and patient selection. Among these, the role of chemotherapy backbone has received considerable attention. One possible explanation is that oxaliplatin induces immunogenic cell death more strongly than cisplatin, which theoretically may enhance its synergistic anti-tumor immunity with ICIs (97); however, this hypothesis is still speculative and requires direct verification through prospective studies. Therefore, improvements in pathological response should not be assumed to be equivalent to survival benefits. In this analysis, among patients who achieved MPR, ICI+CT was associated with longer EFS compared to chemotherapy, supporting the prognostic relevance of pathological response. Nevertheless, these findings do not establish pCR or MPR as reliable alternative endpoints for OS or EFS in this context.

The exploratory subgroup analyses require careful interpretation. These analyses were restricted to ICI+CT versus chemotherapy because the available ICI+CT+A evidence comprised only 2 RCTs and ICI+CRT versus chemotherapy was informed by a single RCT. Across the available clinical and biomarker-defined subgroups, point estimates generally favored ICI+CT, and no statistically significant interaction was observed for pCR, MPR, OS, or EFS. However, the absence of a statistically significant interaction should not be interpreted as evidence of equal benefit across subgroups. The analyses were based on study-level subgroup estimates, with inconsistent reporting, limited statistical power, and no adjustment for multiple comparisons. Likewise, statistical significance within an individual subgroup should not be used to infer a differential treatment effect. These findings are therefore exploratory and may help inform the design of future biomarker-stratified trials.

In the context of radical treatment, perioperative feasibility is as important as tumor response. Our strategy-specific randomized analyses did not indicate that ICI+CT or ICI+CT+A compromised the likelihood of surgical resection, although the R0 resection estimates should be interpreted cautiously and were not intended for between-strategy comparison. The safety evidence remains less mature and less standardized than the pathological-response evidence. Within this review, comparative safety data were available principally for ICI+CT and, to a more limited extent, ICI+CT+A. In contrast, safety evidence for ICI+CRT, ICI+CT+H, and ICI+ICI was derived predominantly from small prospective single-arm cohorts, with substantial variation in treatment composition, perioperative management, and follow-up. More consistent reporting of safety observation windows, treatment exposure, analysis denominators, CTCAE versions, event attribution, surgical delays, and treatment completion is needed before the safety profiles of these strategies can be meaningfully compared. This study has several limitations. First, although randomized controlled trials and prospective single-arm trials were included to maximize coverage of existing evidence, the evidence base is not balanced among the treatment strategies. Comparative estimates were primarily from ICI+CT and ICI+CT+A, while ICI+CRT, ICI+CT+H, and ICI+ICI relied mainly on single-arm studies. Therefore, pooled absolute estimates from single-arm studies should be interpreted descriptively rather than comparatively. Second, the lack of common control groups for most treatment strategies hindered reliable network meta-analysis. Third, subgroup analyses were based on pooled data at the study level and were limited by inconsistent reporting of subgroup-specific outcomes. Although no statistically significant interactions were observed, these analyses may have insufficient power and lacked multiple comparison correction. Fourth, the RCTs contributing to pooled OS/EFS compared perioperative ICI+CT with chemotherapy alone; therefore, the observed survival benefits cannot be attributed solely to the preoperative ICI component or generalized to neoadjuvant-only strategies. Fifth, the follow-up periods in some studies are still relatively short, and mature long-term survival data remain limited, especially for populations screened for biomarkers and for strategies with fewer studies, such as ICI+CT+H, ICI+CRT, and ICI+ICI. In addition, safety observation windows, analysis populations, CTCAE versions, and attribution criteria varied across studies. Although neoadjuvant and perioperative safety results were analyzed separately, the pooled estimates do not represent a standardized common exposure period. Finally, most of the existing evidence comes from Asian populations. Molecular characteristics, surgical practices, perioperative chemotherapy regimens, and postoperative treatment modalities may differ across regions, and whether these findings can be generalized to Western populations requires further prospective validation.

## 5 Conclusions

ICI+CT was associated with improved pCR and MPR and, in perioperative RCTs, OS and EFS versus chemotherapy; it had the largest and most mature randomized evidence base. ICI+CT+A was associated with improved pathological response, but survival evidence was limited to one phase II RCT reporting OS. Comparative evidence for ICI+CRT was limited to one small RCT, and its efficacy and safety require further evaluation. Evidence for ICI+CT+H in HER2-selected tumors and ICI+ICI in dMMR tumors was derived predominantly from small, heterogeneous, or noncomparative studies, without mature comparative survival data. Future trials should move beyond pCR-driven strategies and define optimal patient selection, treatment intensity, and the balance between pathological response, safety, and surgical feasibility.

## Data Availability

The original contributions presented in the study are included in the article/**Supplementary Material**. Further inquiries can be directed to the corresponding authors.
