# Supplementary material for: Impact of immune checkpoint inhibitors as neoadjuvant/perioperative therapy for locally advanced resectable gastric/gastroesophageal junction adenocarcinoma: a systematic review and meta-analysis
Source: Front Immunol. 2026 Sep 25;17:1909466. doi: 10.3389/fimmu.2026.1909466 (PMC13650001; doi:10.3389/fimmu.2026.1909466)
Supplement: Supplementary file 1 [file SupplementaryFile1.pdf]

Supplemental Content

eMethods

eFigure 1. Randomized Clinical Trial Risk of Bias Assessment

eFigure 2. Pooled pCR Rates for Treatment Strategy: ICI+CT (A), ICI+CT+A (B), ICI+CRT (C), ICI+CT+H (D), and ICI+ICI (E)

eFigure 3. Pooled MPR Rates for Treatment Strategy: ICI+CT (A), ICI+CT+A (B), ICI+CRT (C), ICI+CT+H (D), and ICI+ICI (E)

eFigure 4. Pooled Risk Ratios of pCR for ICI+CT Versus Chemotherapy by PD-L1 CPS cutoff of 1 (A), 5 (B), and 10 (C), MMR Status (D) , Lauren Classification (E) , ECOG Performance Status (F), Sex (G), Age (H), and Primary Tumor Location (I)

eFigure 5. Pooled Risk Ratios of MPR for ICI+CT Versus Chemotherapy by PD-L1 CPS cutoff of 1 (A), 5 (B), and MMR Status (C)

eFigure 6. Pooled Hazard Ratios of EFS among Patients Achieving MPR in Perioperative ICI+CT Versus Chemotherapy

eFigure 7. Pooled 1-year OS (A), EFS (B), and PFS (C) Rates

eFigure 8. Pooled 2-year OS (A), EFS (B), and PFS (C) Rates

eFigure 9. Pooled 3-year OS (A), EFS (B), and PFS (C) Rates

eFigure 10. Pooled Hazard Ratios of OS for Perioperative ICI+CT Versus Chemotherapy by Lauren Classification (A) , ECOG Performance Status (B), Sex (C), Age (D), Primary Tumor Location (E), Region (F), and MMR Status (G)

eFigure 11. Pooled Hazard Ratios of EFS for Perioperative ICI+CT Versus Chemotherapy by PD-L1 CPS cutoff of 5 (A), 10 (B), Lauren's Classification (C) , ECOG Performance Status (D), Age (E), Sex (F), Primary Tumor Location (G), and Region (H), and MMR Status (I)

eFigure 12. Pooled Risk Ratios of Surgical Resection Rates (A) and R0 Resection Rates (B) Across Randomized Clinical Trials

eFigure 13. Pooled Surgical Resection Rates for Treatment Strategy: ICI+CT (A), ICI+CT+A (B), ICI+CRT (C), ICI+CT+H (D), and ICI+ICI (E)

eFigure 14. Pooled R0 Resection Rates for Treatment Strategy: ICI+CT (A), ICI+CT+A (B), ICI+CRT (C), and ICI+CT+H (D)

eFigure 15. Pooled DCR Rates (A) and ORR Rates (B)

eFigure 16. Pooled Risk Ratios of TRAEs (Any Grade) during the Perioperative Period (A), TRAEs (Any Grade) during the Neoadjuvant Period (B), irAEs (Any Grade) during the Perioperative Period (C), and irAEs (Any Grade) during the Neoadjuvant Period (D) Across Randomized Clinical Trials

eFigure 17. Pooled TRAEs (Grade ≥3) (A) and irAEs (Grade ≥3) (B) Rates during Perioperative Therapy
eFigure 18. Pooled TRAEs (Grade ≥3) (A) and irAEs (Grade ≥3) (B) Rates during Neoadjuvant Therapy
eFigure 19. Pooled TRAEs (Any Grade) (A) and irAEs (Any Grade) (B) Rates during Perioperative Therapy
eFigure 20. Pooled TRAEs (Any Grade) (A) and irAEs (Any Grade) (B) Rates during Neoadjuvant Therapy
eFigure 21. Pooled Surgical Complications (Any grade) (A) and Surgical Complications (Grade ≥3) (B) Rates
eFigure 22. Leave-One-Out Sensitivity Analyses of Pooled Risk Ratios for pCR (A) and MPR (B) for ICI+CT Versus Chemotherapy
eFigure 23. Leave-One-Out Sensitivity Analysis of Pooled pCR Rates for Treatment Strategy: ICI+CT (A), ICI+CT+A (B), Single-ICI+CRT (C), Disitamab Vedotin-based ICI+CT+H (D), and Trastuzumab-based ICI+CT+H (E)
eFigure 24. Leave-One-Out Sensitivity Analysis of Pooled MPR Rates for Treatment Strategy: ICI+CT (A), ICI+CT+A (B), Single-ICI+CRT (C), Disitamab Vedotin-based ICI+CT+H (D), and Trastuzumab-based ICI+CT+H (E)
eFigure 25. Funnel Plot for Publication Bias Assessment of pCR in the ICI+CT Versus Chemotherapy Comparison
eTable 1. Differences between protocol and review
eTable 2. Search Strategy for PubMed
eTable 3. Search Strategy for Web of Science
eTable 4. Search Strategy for Embase
eTable 5. Search Strategy for the Cochrane Library
eTable 6. Report-to-trial linkage and handling of multiple reports
eTable 7. Basic Characteristics of Studies Included in the Single-arm Meta-analysis
eTable 8. Methodological Quality of Single-arm Studies Assessed with MINORS Checklist
eTable 9. Study-Level Characteristics of TRAEs and irAEs of RCTs Included in the Pairwise Meta-analysis
eTable 10. Study-Level Characteristics of TRAEs and irAEs in Studies Included in the Single-Arm Meta-analysis
eTable 11. Sensitivity Analyses of pairwise meta-analyses
eTable 12. Sensitivity Analyses of Single-Arm Meta-analyses

eTable 13. Comparison of Pairwise Meta-Analysis Results Between ITT and PP Populations
eTable 14. Comparison of Single-Arm Meta-Analysis Results Between ITT and PP Populations
eTable 15. The Overall Certainty of Evidence
eTable 16. Comparison of Overlapping Systematic Reviews and Meta-Analyses of Neoadjuvant/Perioperative ICI-based Treatment in Resectable Gastric or Gastroesophageal Junction Cancer

eMethods

Extracted Data

Data were independently extracted by two investigators, with disagreements resolved by consultation with a third investigator. The intention-to-treat (ITT) population was used as the primary analysis population for efficacy endpoints (pCR, MPR, R0 resection rate, ORR, and DCR). For endpoints related to surgery (pCR, MPR, R0 resection rate), patients who did not undergo surgery or did not have pathological evaluation were considered as not having experienced the event. For ORR and DCR, patients who did not undergo efficacy assessment for any reason were considered as not having experienced the event in the ITT analysis. Corresponding data based on the per-protocol (PP) population were additionally extracted or calculated for sensitivity analyses. The pCR rate was defined as the percentage of patients with no residual tumor cells in the primary tumor. MPR was defined as residual tumor less than 10% in the primary tumor. Radiologic response was evaluated using RECIST (version 1.1). The ORR was defined as the proportion of patients with CR and PR, and the DCR was defined as the proportion of patients with CR, PR, and SD.

For survival outcomes, HRs and their 95%CIs were extracted directly from the articles or supplementary materials for pooled analysis. Survival rates at specific time points (1, 2, and 3 years) were pooled based on the actual population that underwent survival analysis. If the exact values were not directly provided in the original text, two investigators independently used Engauge Digitizer v4.1 software to extract coordinate data from Kaplan-Meier curves to estimate the survival rates at the corresponding time points, and the mean of the two values was taken as the final value; if at a given time point the curve had no patients at risk or the curve had terminated, extrapolation was not performed. OS was defined as the time from randomization or enrolment to death from any cause. Endpoint labels and definitions for EFS and PFS were retained as reported by each source study. EFS and PFS were extracted and synthesized separately and were not pooled or treated as interchangeable outcomes.

For safety outcomes, aggregate any-grade and grade ≥3 TRAEs and irAEs were synthesized separately for the study-defined neoadjuvant and perioperative safety periods. Safety results were classified by treatment phase only when explicitly reported in the source; phase-unspecified results were excluded from phase-specific pooling. Perioperative results included those explicitly reported over both preoperative and postoperative treatment. When both neoadjuvant and perioperative

results were available, they were included in the corresponding separate analyses. Exact observation boundaries were not standardized when unavailable, and the pooled estimates therefore represent study-defined phase-specific safety periods rather than a common fixed window. The reported safety or treated population was used as the denominator, generally comprising patients who received at least one dose of study treatment; enrolled or randomized populations were accepted when all patients received treatment. Only events explicitly reported as TRAEs or irAEs were included, whereas all-cause AEs, TEAEs, and adverse events of special interest collected irrespective of attribution were excluded. Grade 3-4 or grade 3-5 events were categorized as grade ≥3. Study-level observation windows, denominators, CTCAE versions, and attribution criteria are reported in eTables 9 and 10. Postoperative complications were pooled based on patients who underwent surgery, and only studies that explicitly reported using the Clavien-Dindo classification system were included; studies that did not explicitly use the Clavien-Dindo classification were not included in the quantitative pooling.

Across all forest plots in the main article and Supplement, k denotes the number of unique underlying trials contributing to each pooled estimate, and N denotes the corresponding outcome-specific analysis denominator. For pCR, MPR, surgical resection, R0 resection, DCR, and ORR, N represents the intention-to-treat population. For OS, EFS, and PFS, N represents the source-reported survival-analysis population. For safety outcomes, N represents the source-reported safety or treated population, generally comprising participants who received at least 1 dose of study treatment. For postoperative complications, N represents patients who underwent surgery and were included in the complication analysis. In subgroup analyses, N represents participants contributing to the corresponding subgroup-specific estimate. For multi-panel, k and N are specific to the respective panel or estimate. Multiple reports from the same trial were counted once for k, and no participant was counted more than once within any single outcome analysis.

eFigure 1. Randomized Clinical Trial Risk of Bias Assessment

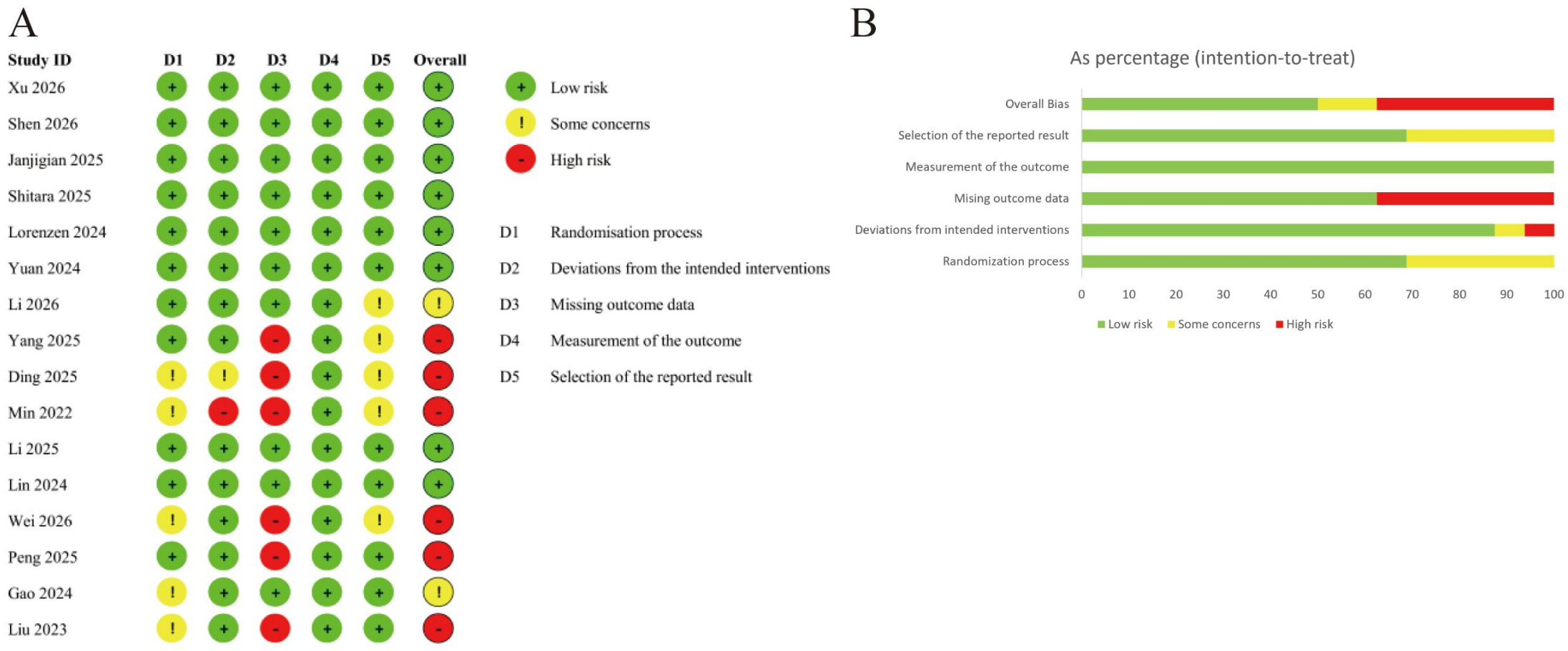

eFigure 2. Pooled pCR Rates for Treatment Strategy: ICI+CT (A), ICI+CT+A (B), ICI+CRT (C), ICI+CT+H (D), and ICI+ICI (E)

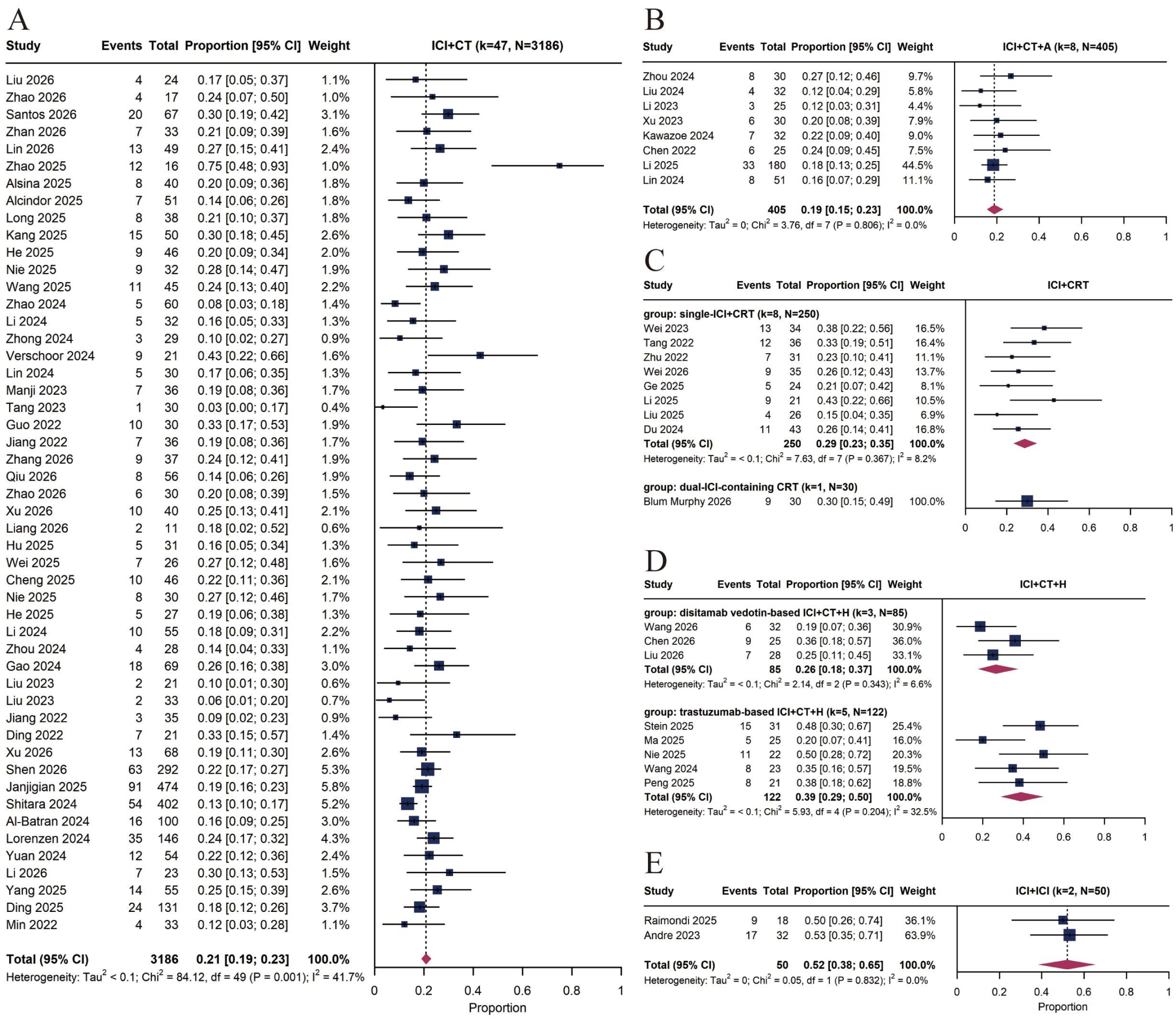

eFigure 3. Pooled MPR Rates for Treatment Strategy: ICI+CT (A), ICI+CT+A (B), ICI+CRT (C), ICI+CT+H (D), and ICI+ICI (E)

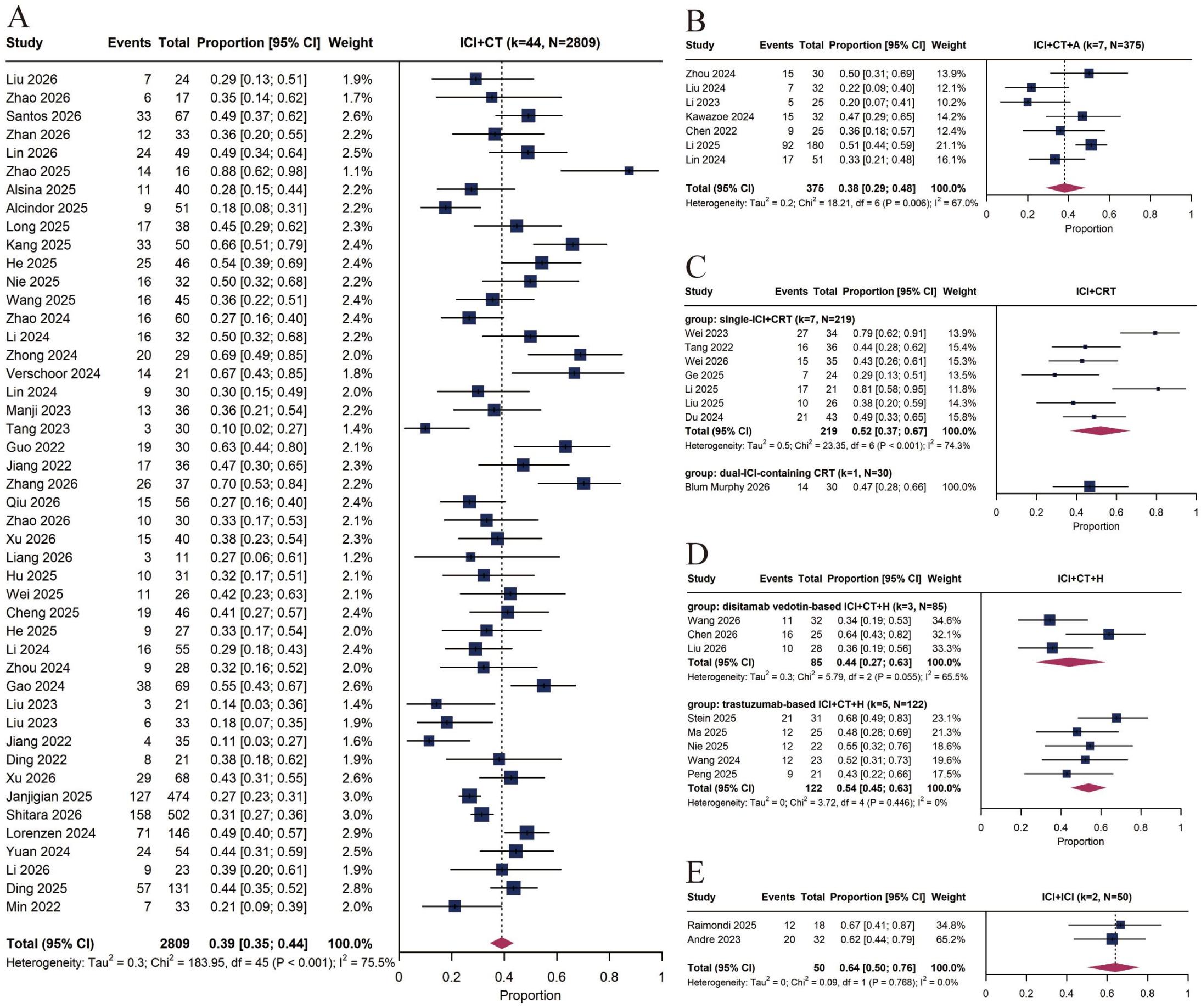

eFigure 4. Pooled Risk Ratios of pCR for ICI+CT Versus Chemotherapy by PD-L1 CPS cutoff of 1 (A), 5 (B), and 10 (C), MMR Status (D) , Lauren Classification (E) , ECOG Performance Status (F), Sex (G), Age (H), and Primary Tumor Location (I)

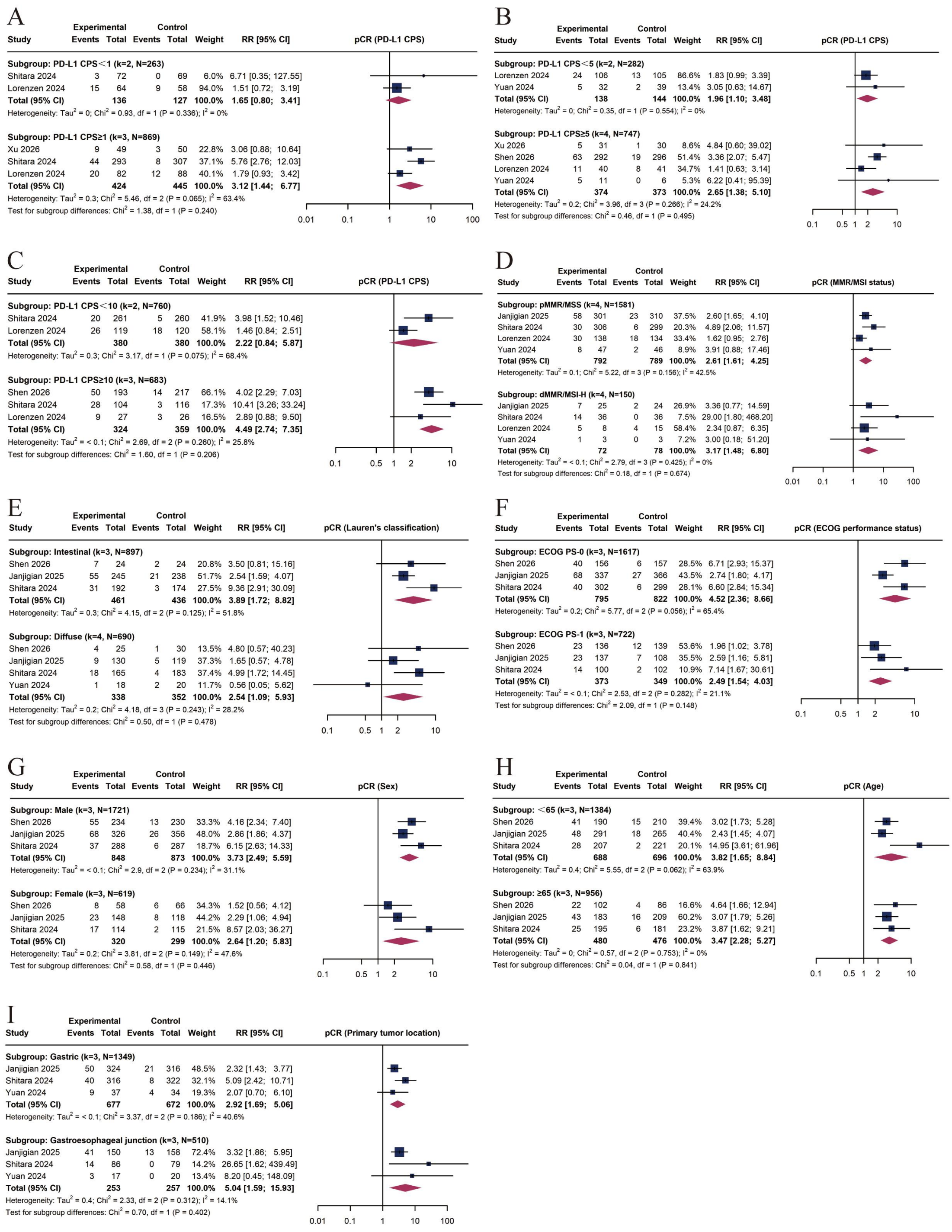

eFigure 5. Pooled Risk Ratios of MPR for ICI+CT Versus Chemotherapy by PD-L1 CPS cutoff of 1 (A), 5 (B), and MMR Status (C)

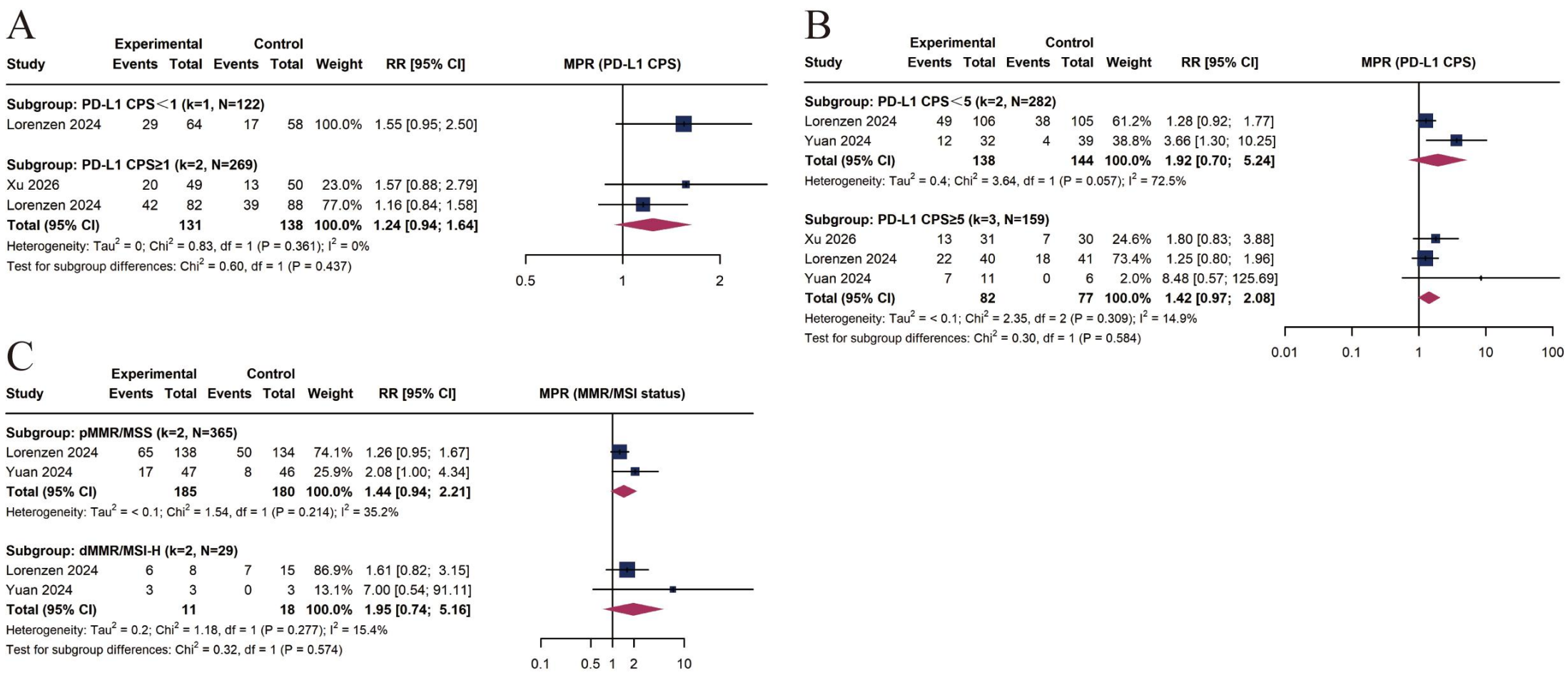

eFigure 6. Pooled Hazard Ratios of EFS among Patients Achieving MPR in Perioperative ICI+CT Versus Chemotherapy

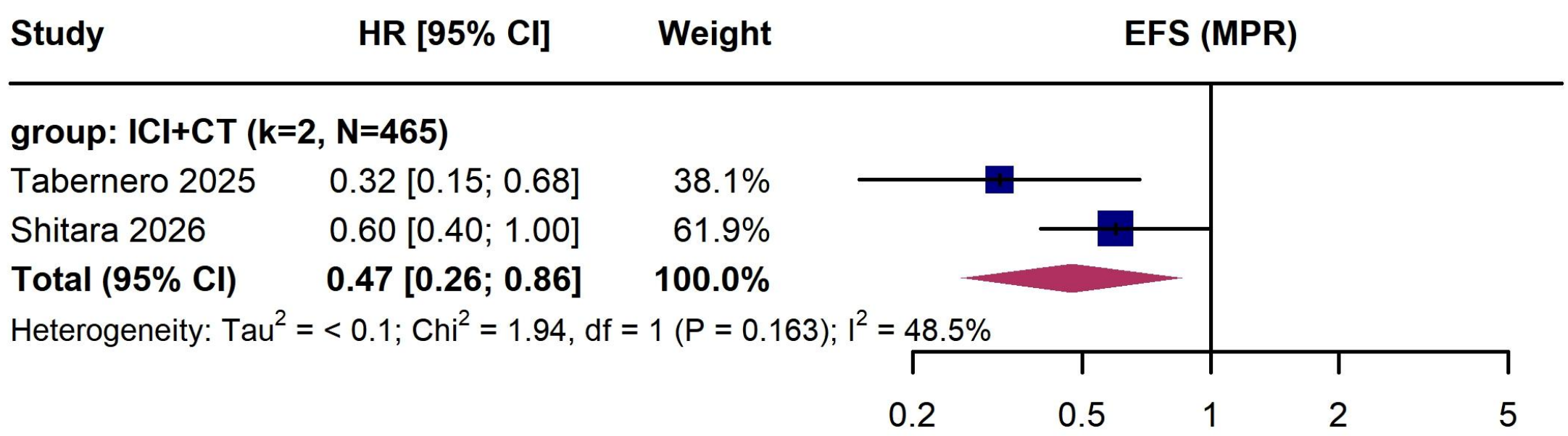

eFigure 7. Pooled 1-year OS (A), EFS (B), and PFS (C) Rates

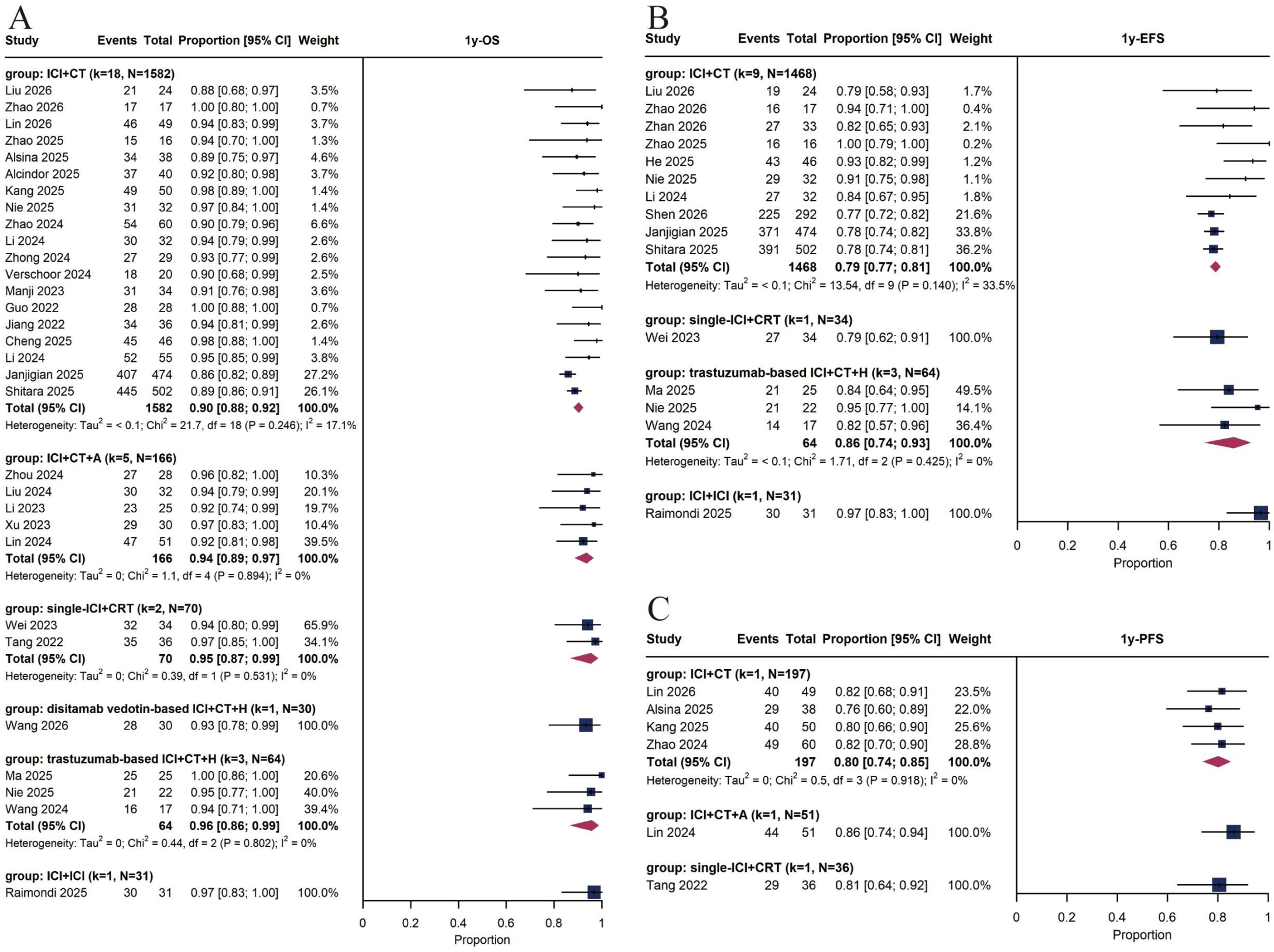

eFigure 8. Pooled 2-year OS (A), EFS (B), and PFS (C) Rates

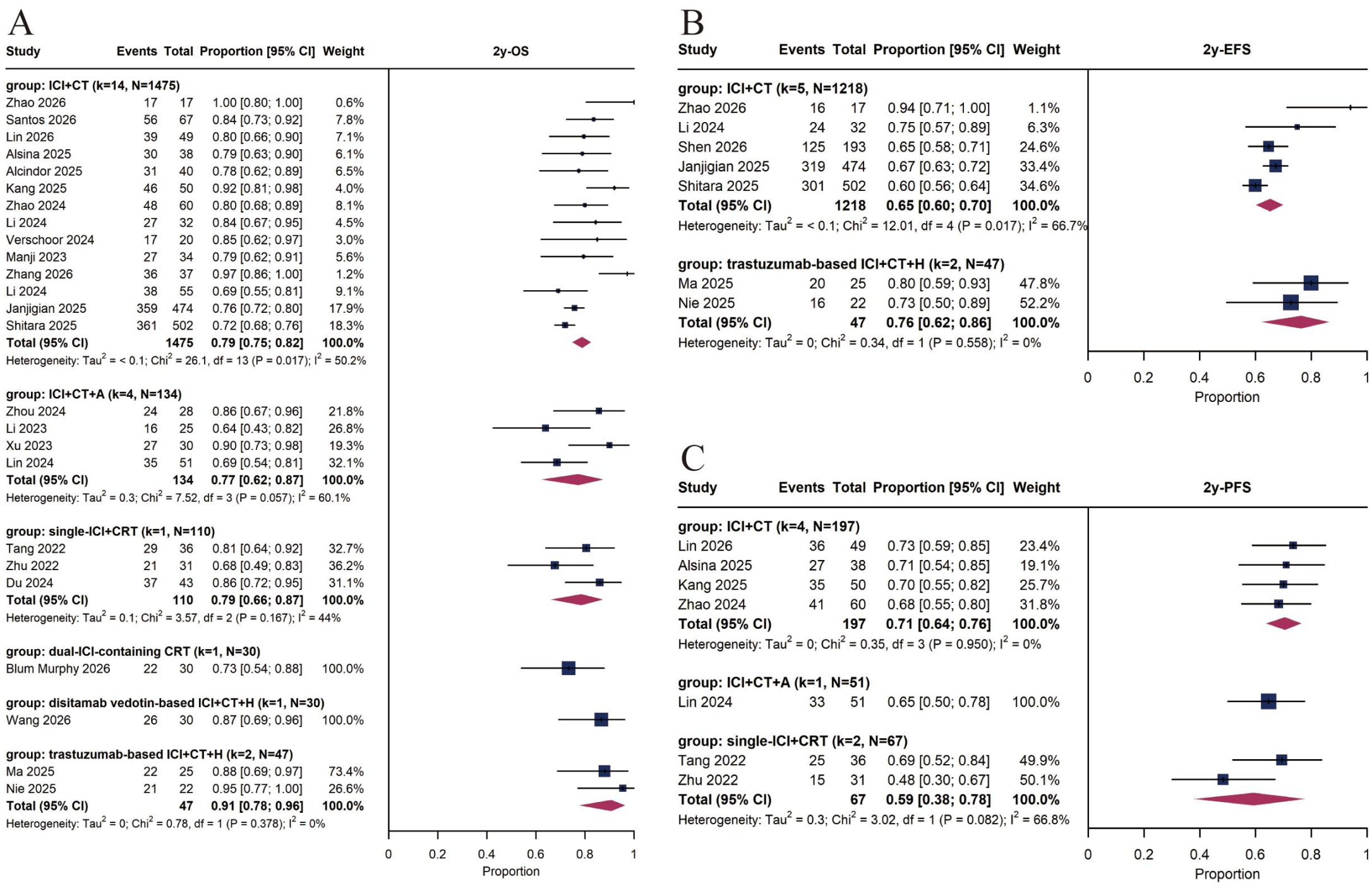

eFigure 9. Pooled 3-year OS (A), EFS (B), and PFS (C) Rates

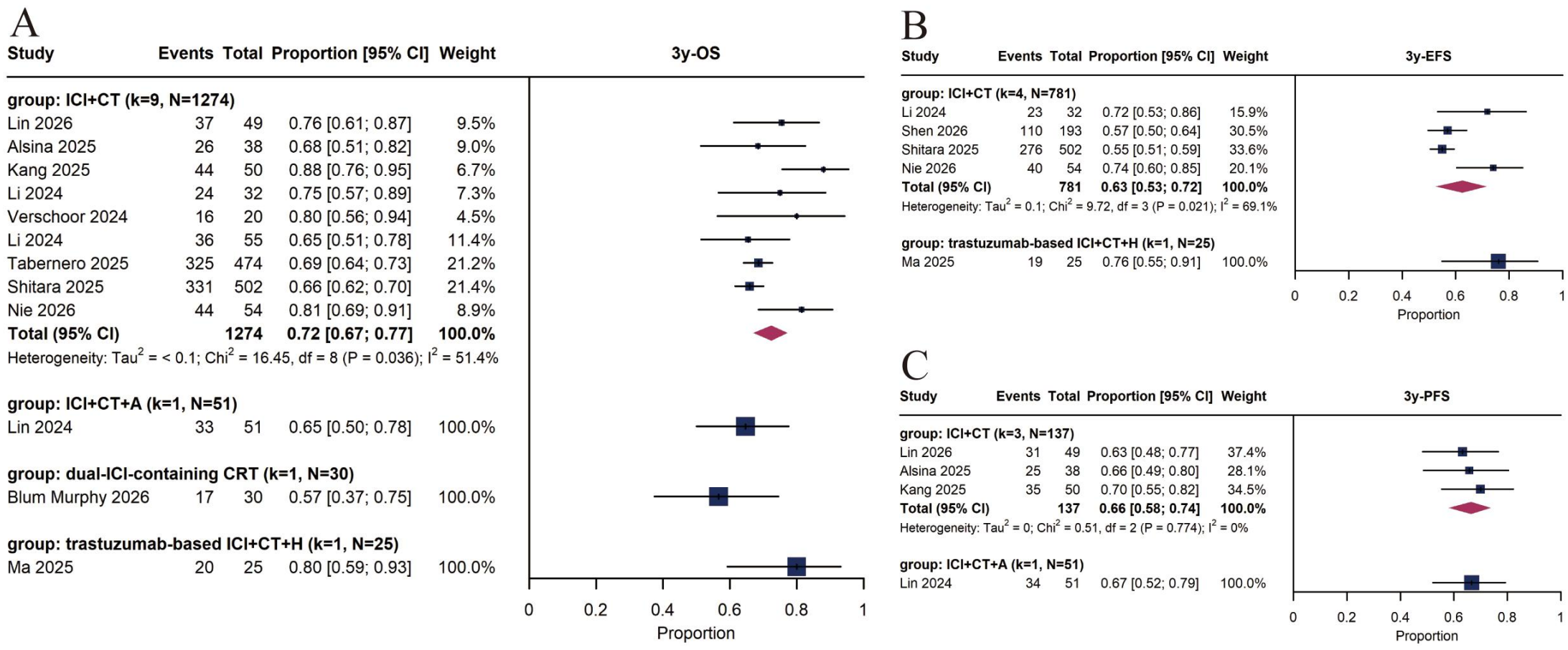

eFigure 10. Pooled Hazard Ratios of OS for Perioperative ICI+CT Versus Chemotherapy by Lauren Classification (A) , ECOG Performance Status (B), Sex (C), Age (D), Primary Tumor Location (E), Region (F), and MMR Status (G)

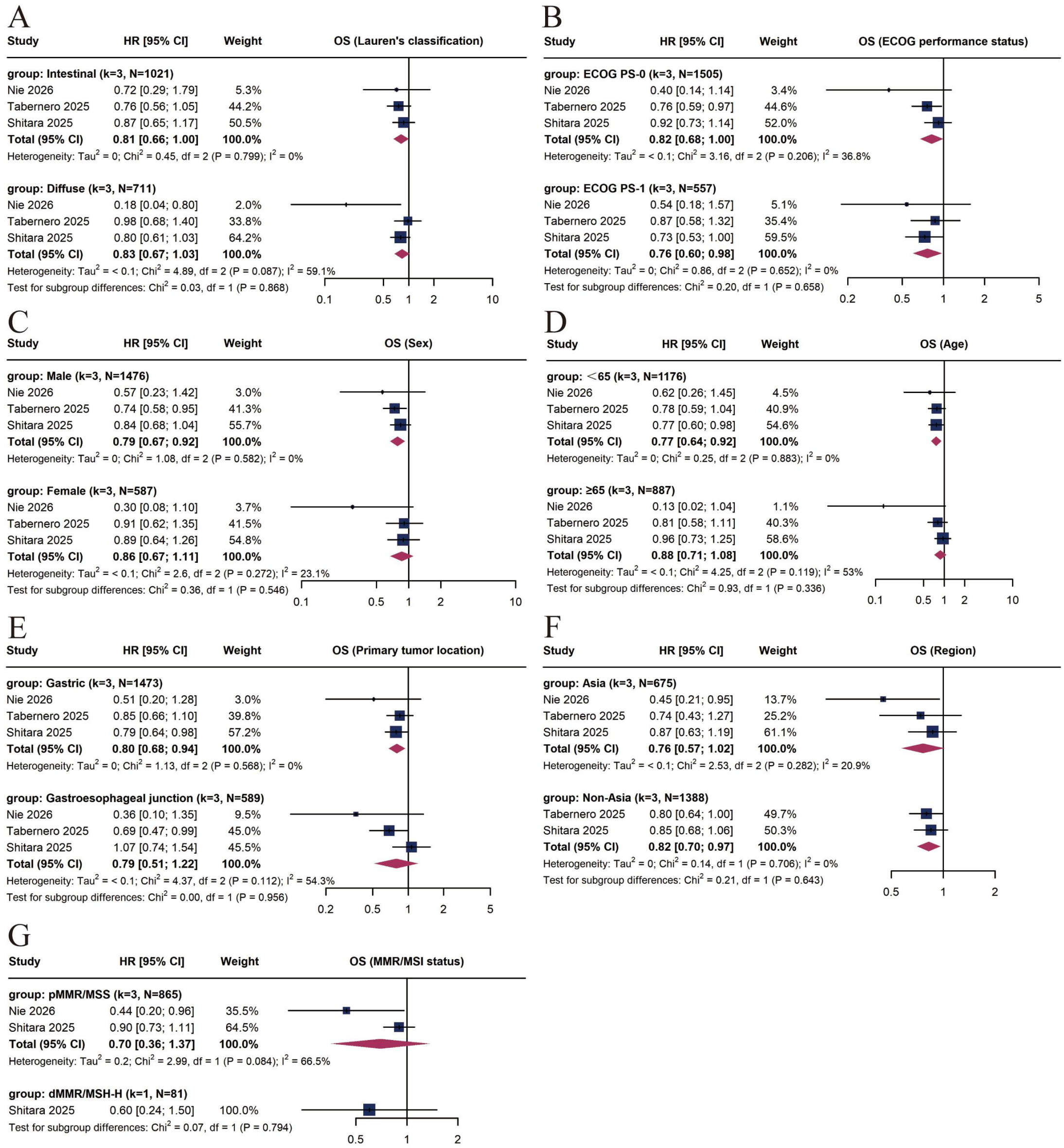

eFigure 11. Pooled Hazard Ratios of EFS for Perioperative ICI+CT Versus Chemotherapy by PD-L1 CPS cutoff of 5 (A), 10 (B), Lauren's Classification (C) , ECOG Performance Status (D), Age (E), Sex (F), Primary Tumor Location (G), and Region (H), and MMR Status (I)

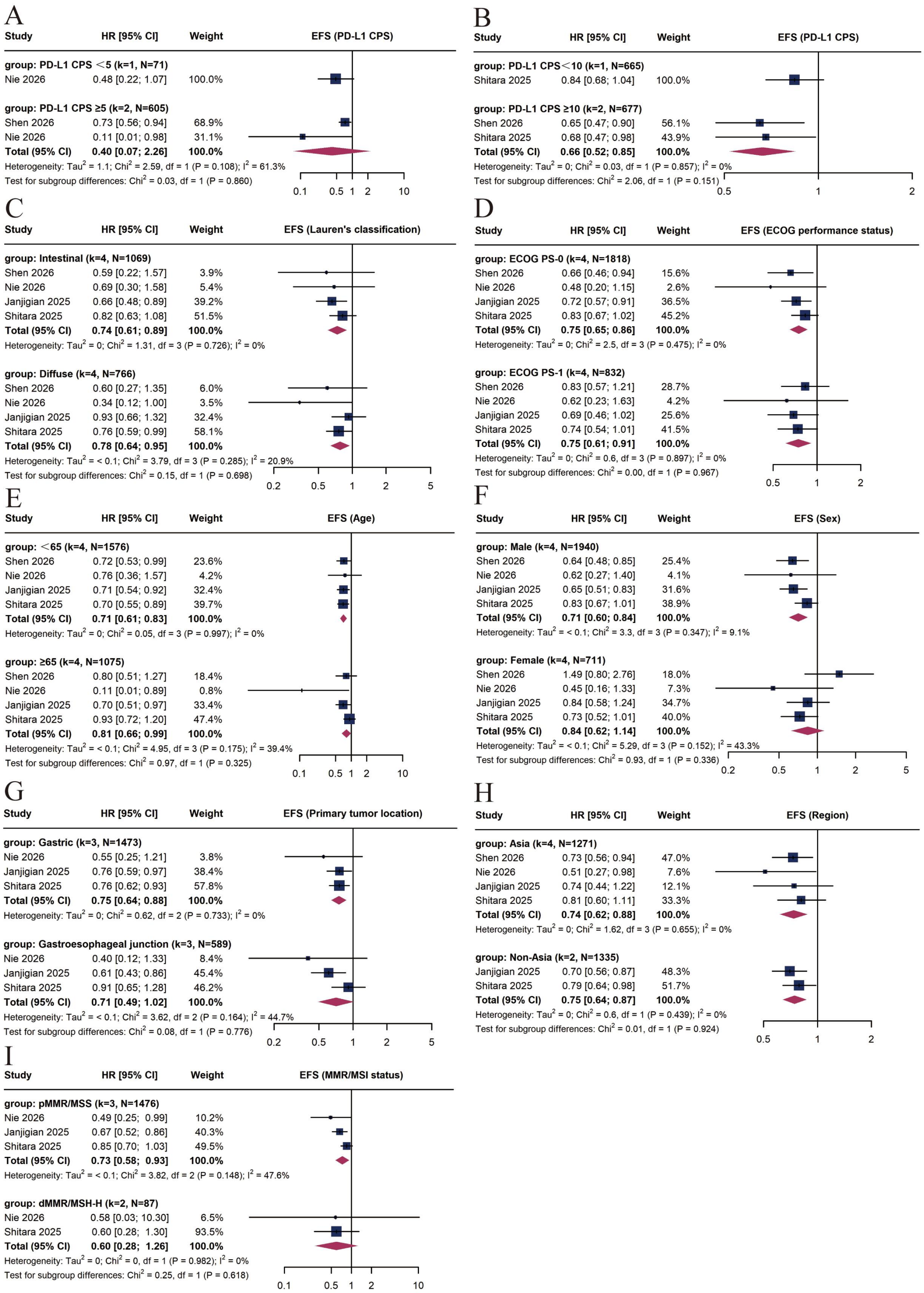

eFigure 12. Pooled Risk Ratios of Surgical Resection Rates (A) and R0 Resection Rates (B) Across Randomized Clinical Trials

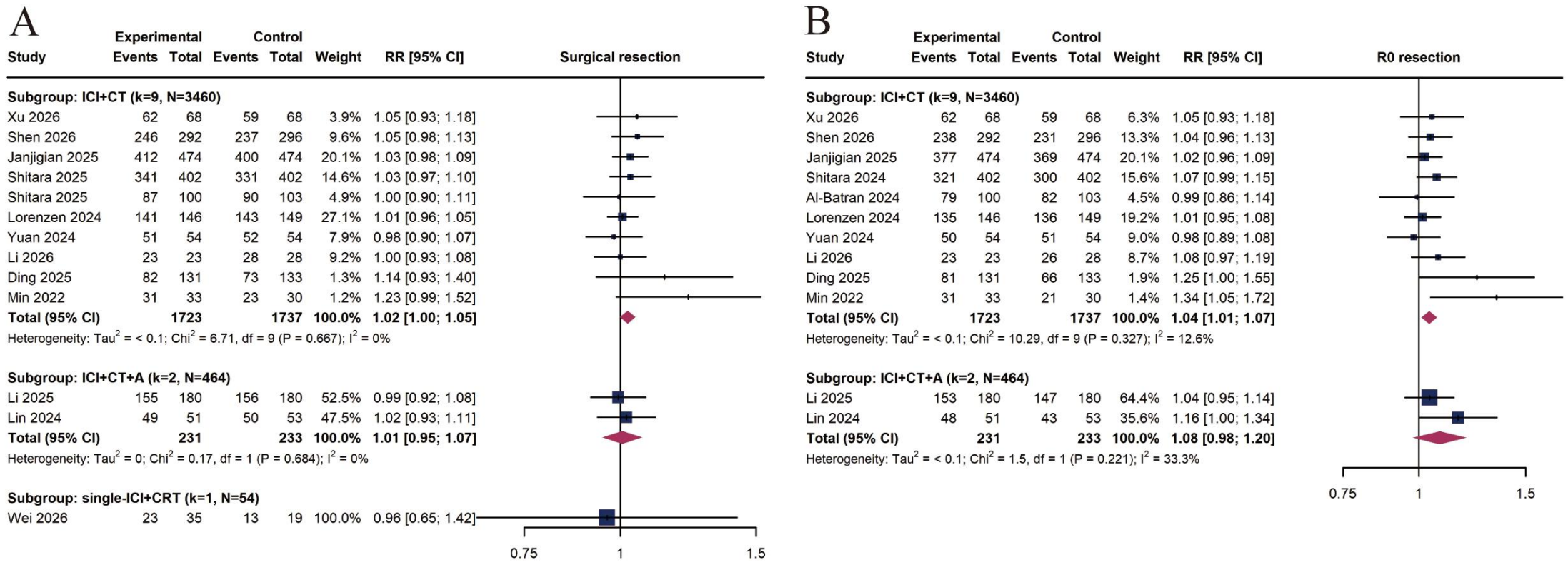

eFigure 13. Pooled Surgical Resection Rates for Treatment Strategy: ICI+CT (A), ICI+CT+A (B), ICI+CRT (C), ICI+CT+H (D), and ICI+ICI (E)

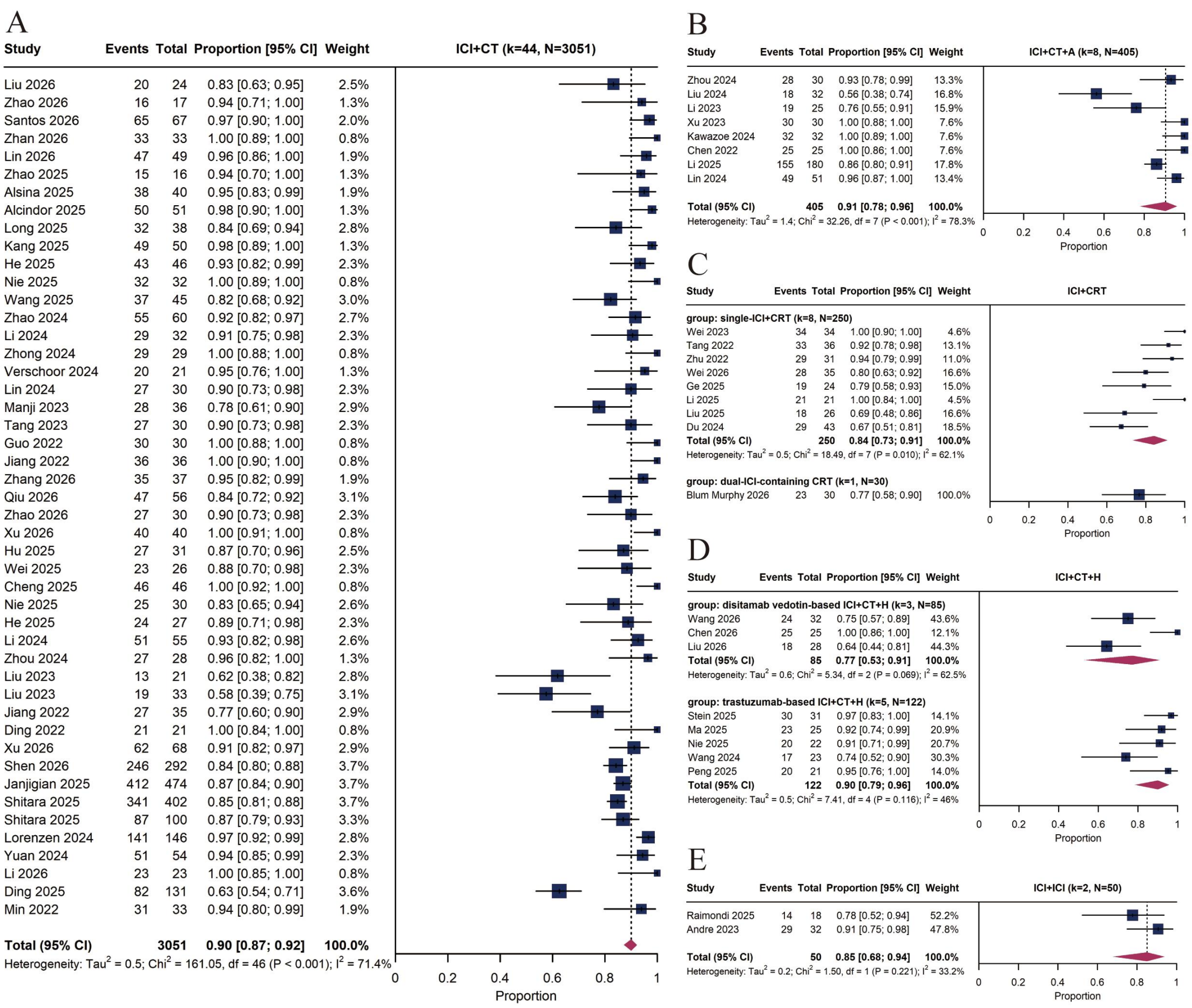

eFigure 14. Pooled R0 Resection Rates for Treatment Strategy: ICI+CT (A), ICI+CT+A (B), ICI+CRT (C), and ICI+CT+H (D)

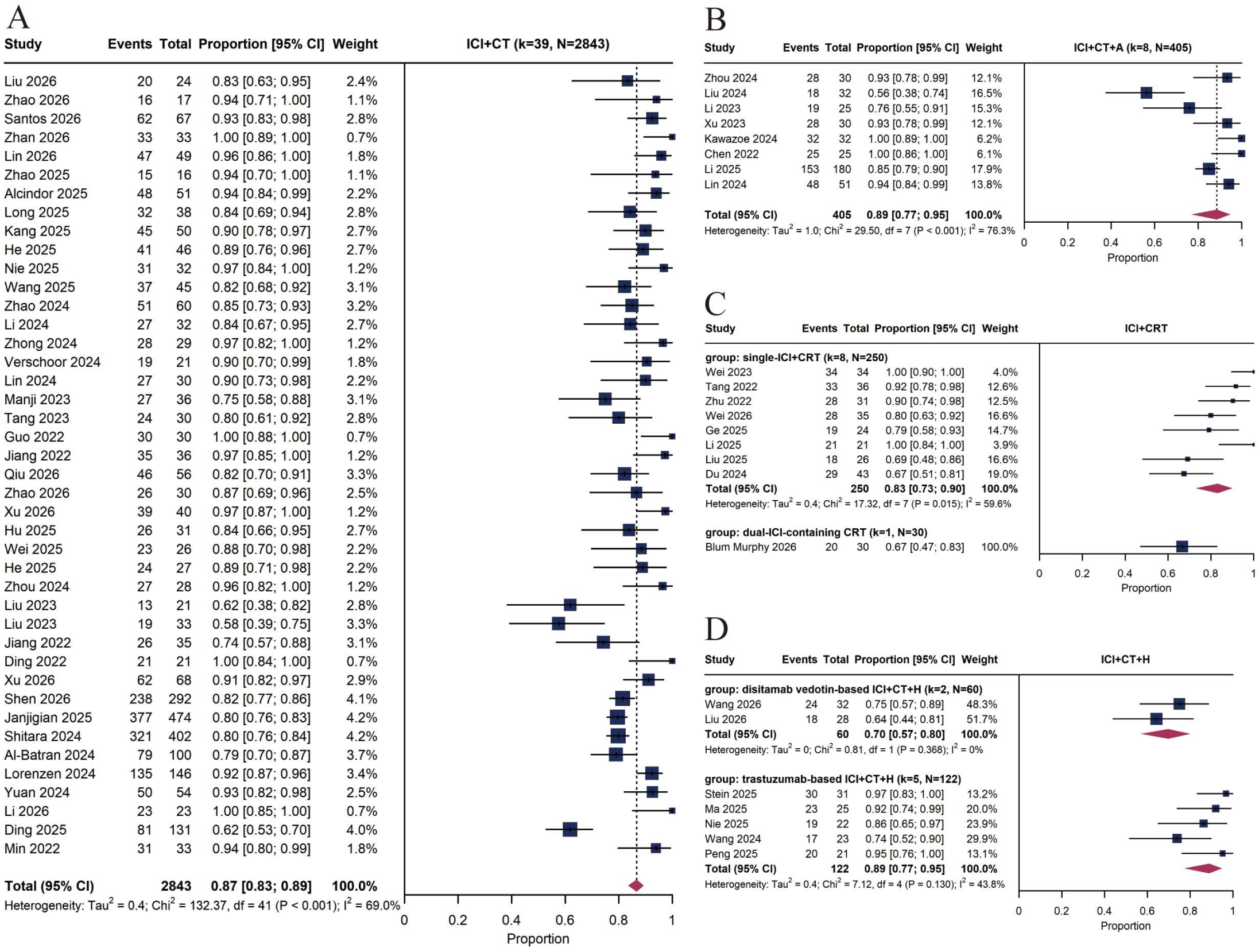

eFigure 15. Pooled DCR Rates (A) and ORR Rates (B)

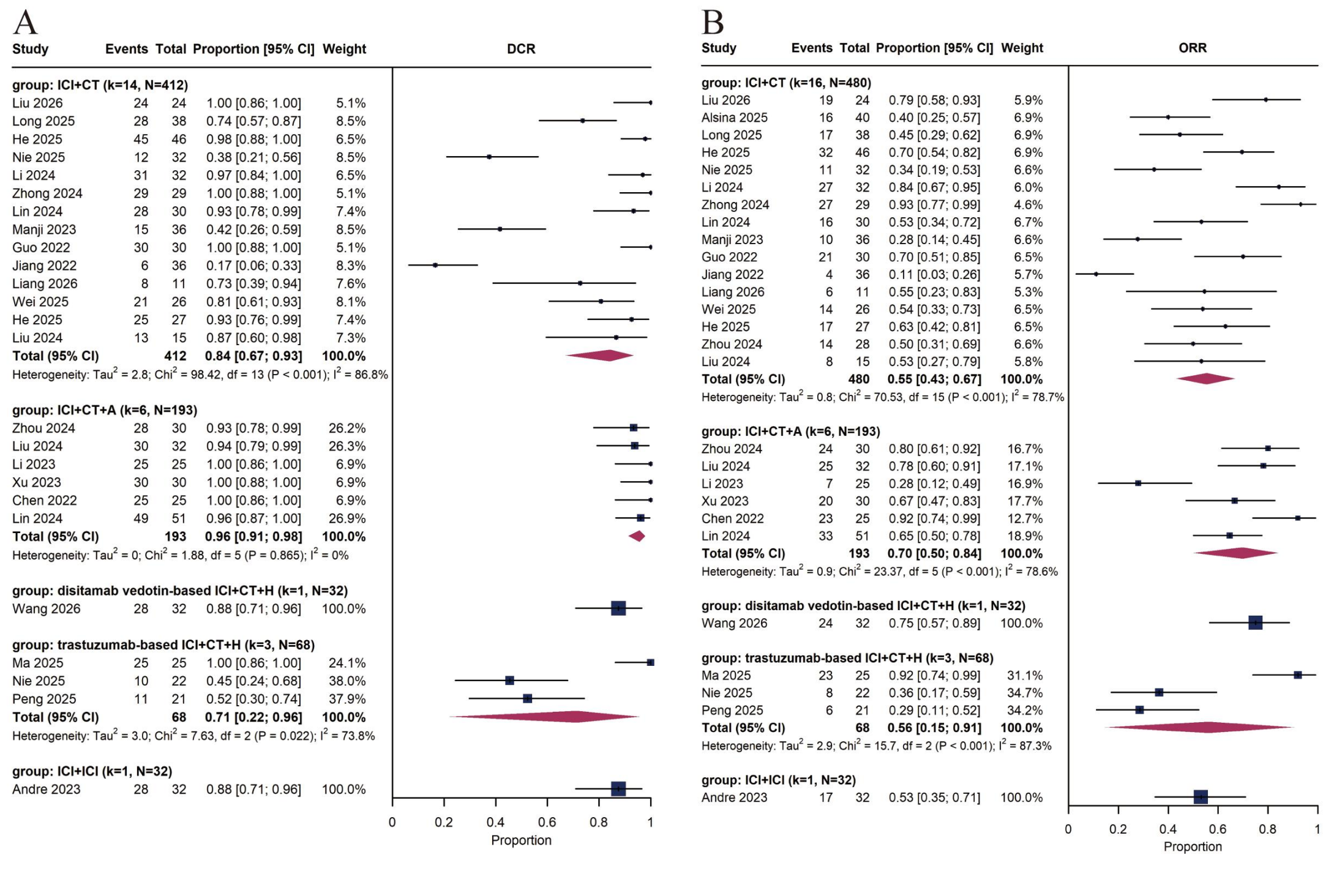

eFigure 16. Pooled Risk Ratios of TRAEs (Any Grade) during the Perioperative Period (A), TRAEs (Any Grade) during the Neoadjuvant Period (B), irAEs (Any Grade) during the Perioperative Period (C), and irAEs (Any Grade) during the Neoadjuvant Period (D) Across Randomized Clinical Trials

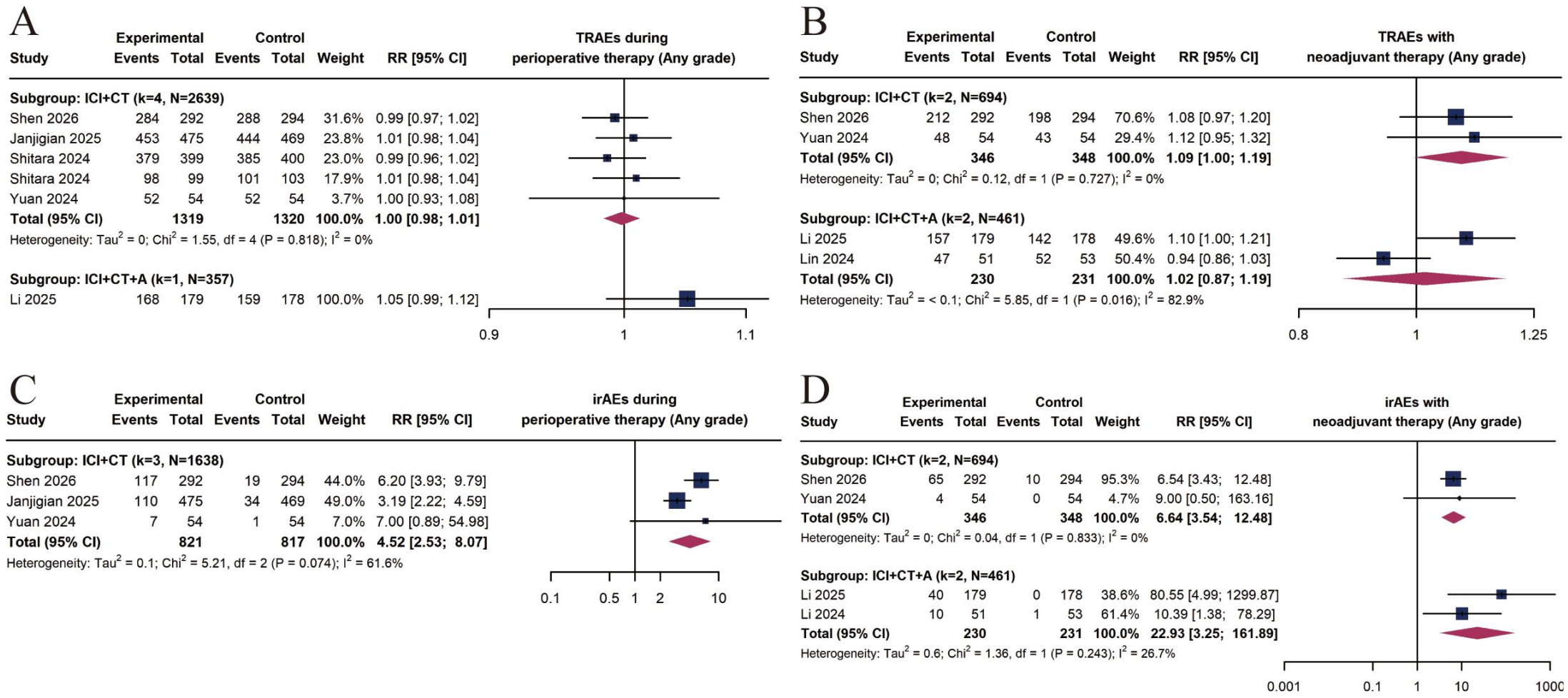

eFigure 17. Pooled TRAEs (Grade ≥3) (A) and irAEs (Grade ≥3) (B) Rates during Perioperative Therapy

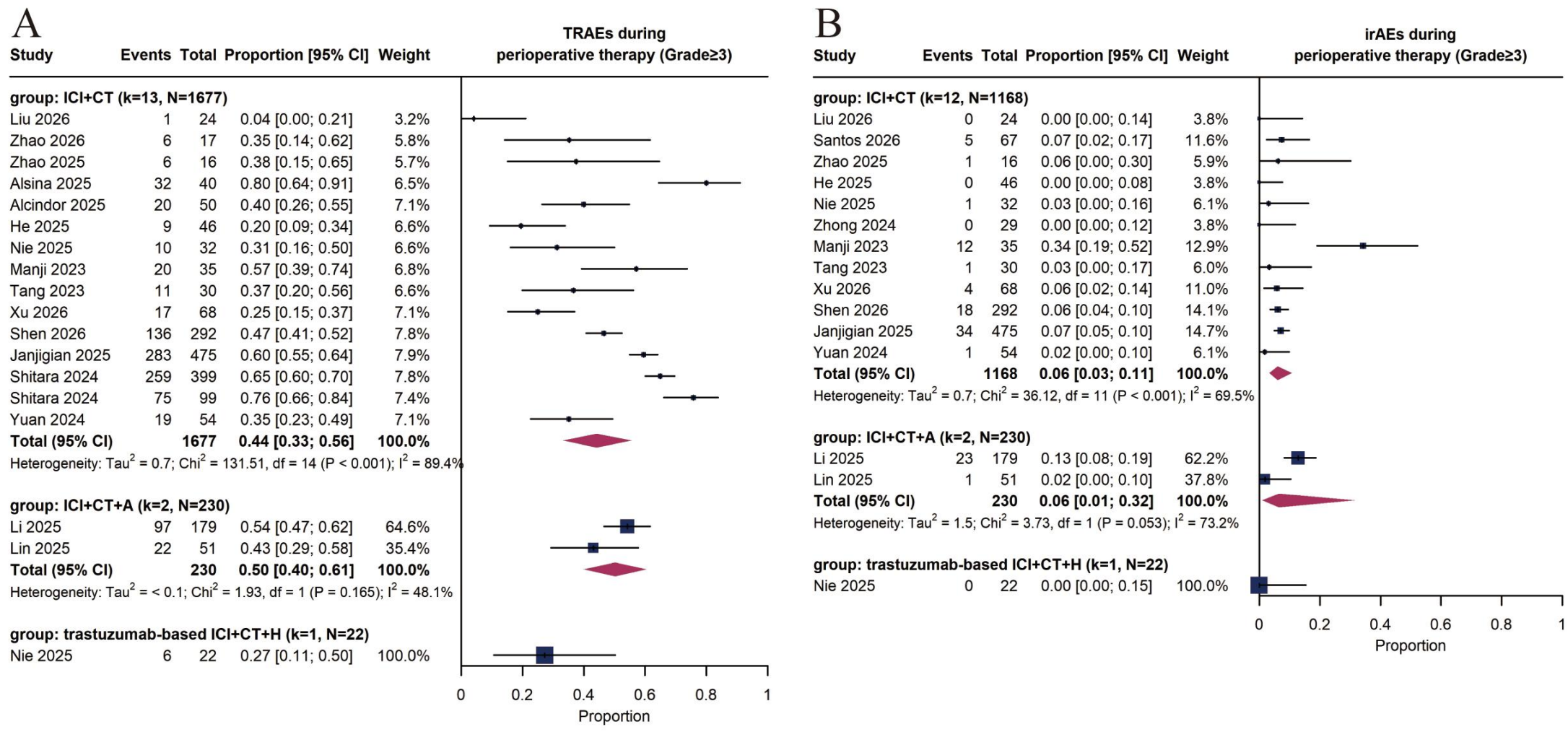

eFigure 18. Pooled TRAEs (Grade ≥3) (A) and irAEs (Grade ≥3) (B) Rates during Neoadjuvant Therapy

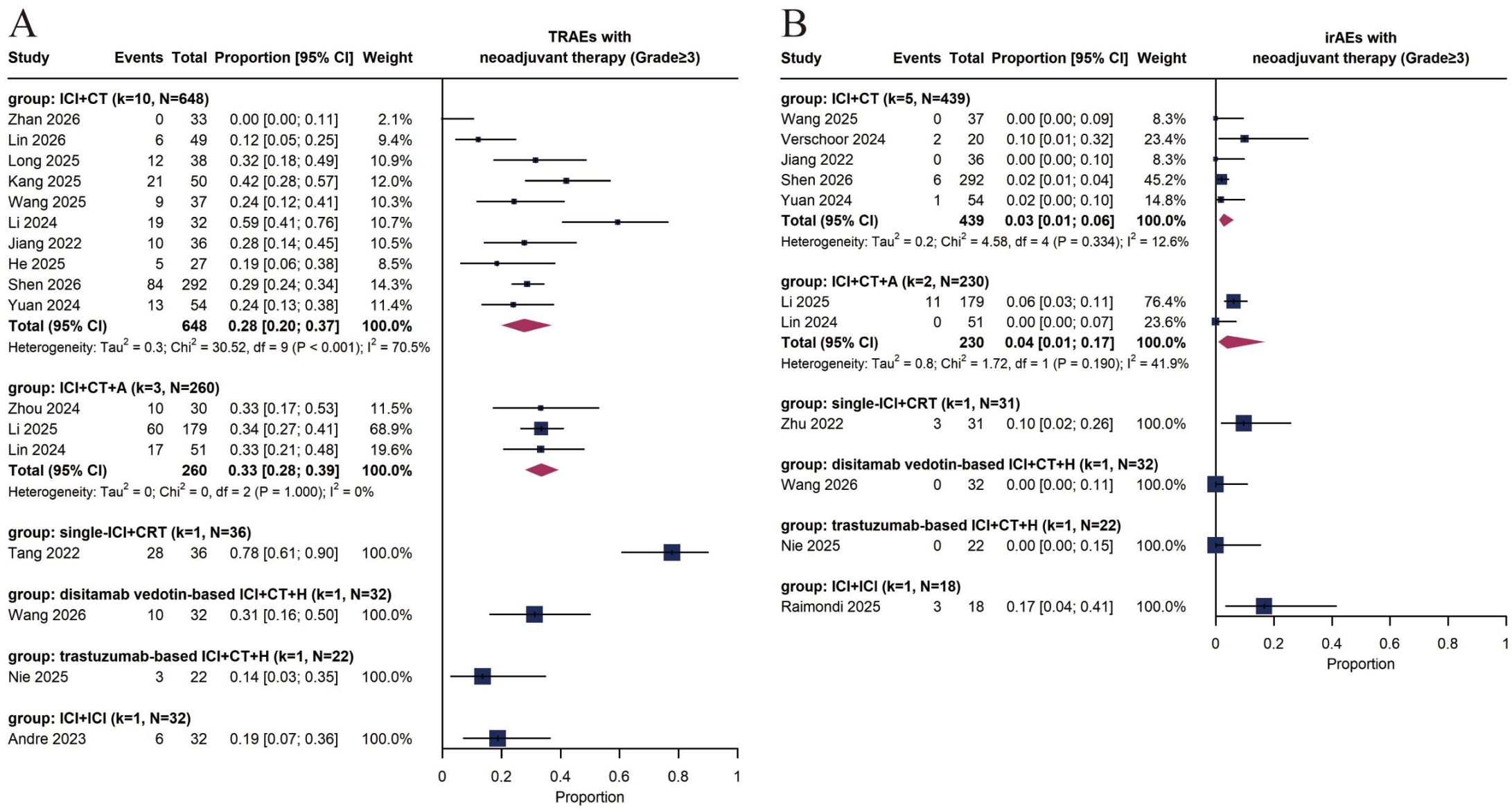

eFigure 19. Pooled TRAEs (Any Grade) (A) and irAEs (Any Grade) (B) Rates during Perioperative Therapy

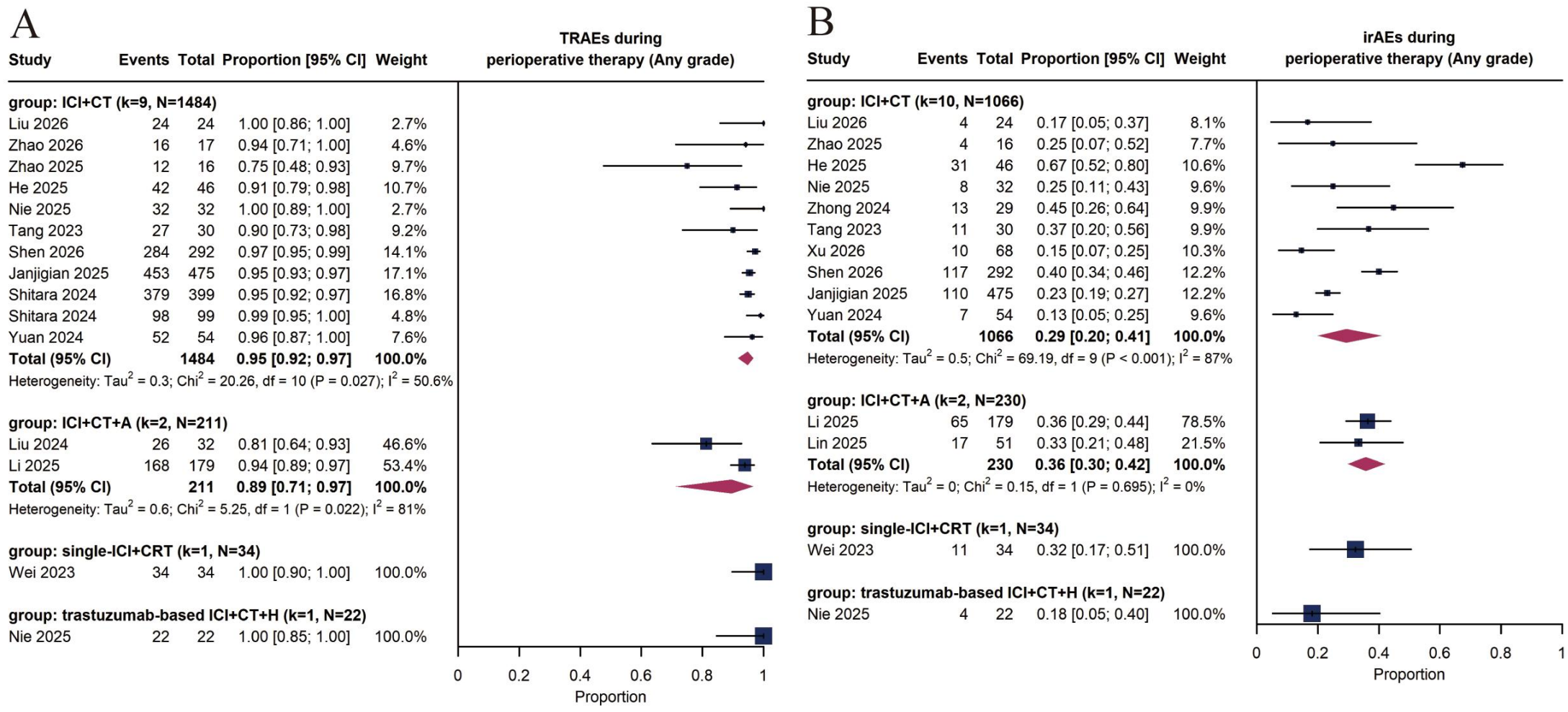

eFigure 20. Pooled TRAEs (Any Grade) (A) and irAEs (Any Grade) (B) Rates during Neoadjuvant Therapy

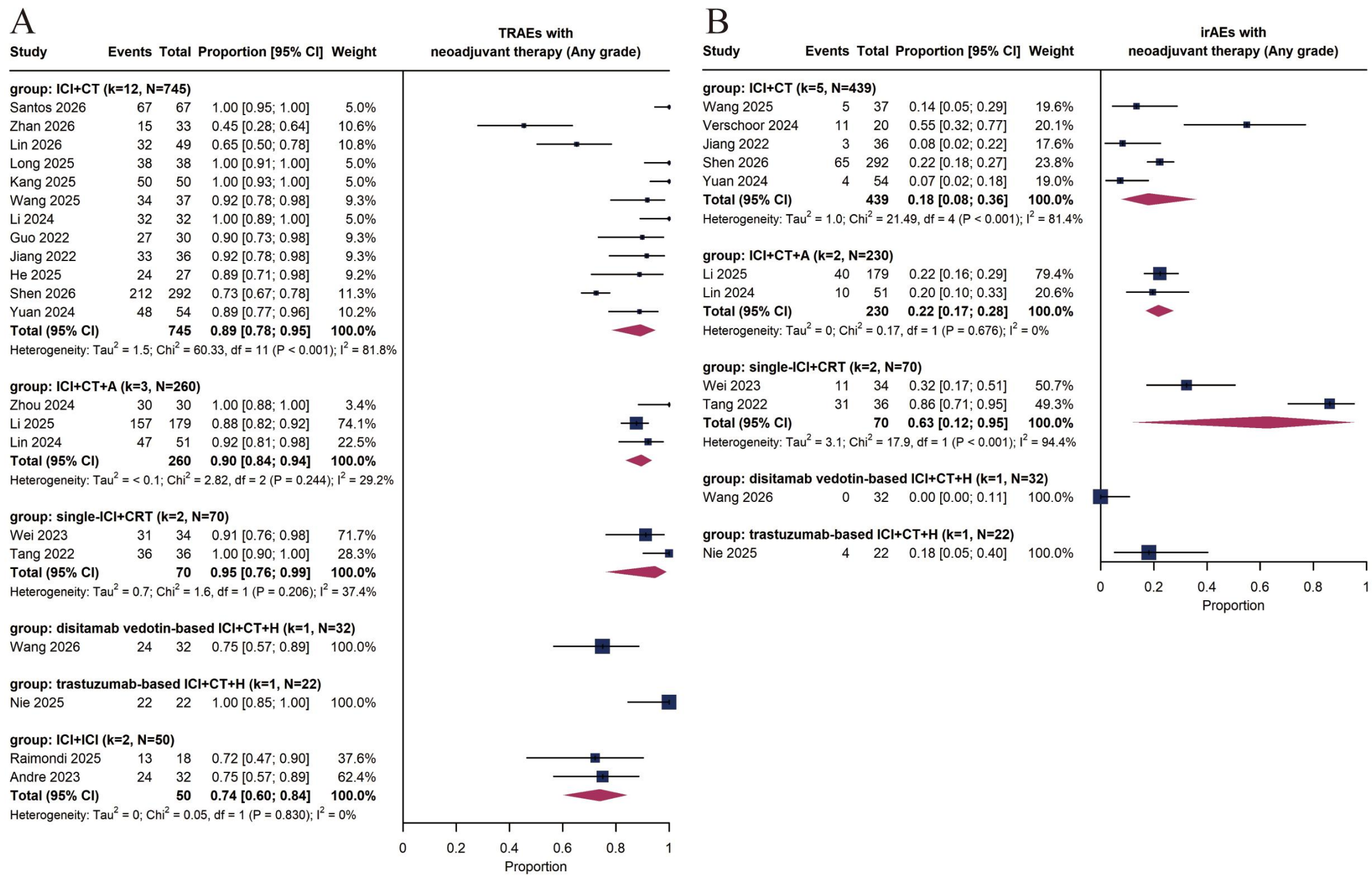

eFigure 21. Pooled Surgical Complications (Any grade) (A) and Surgical Complications (Grade ≥3) (B) Rates

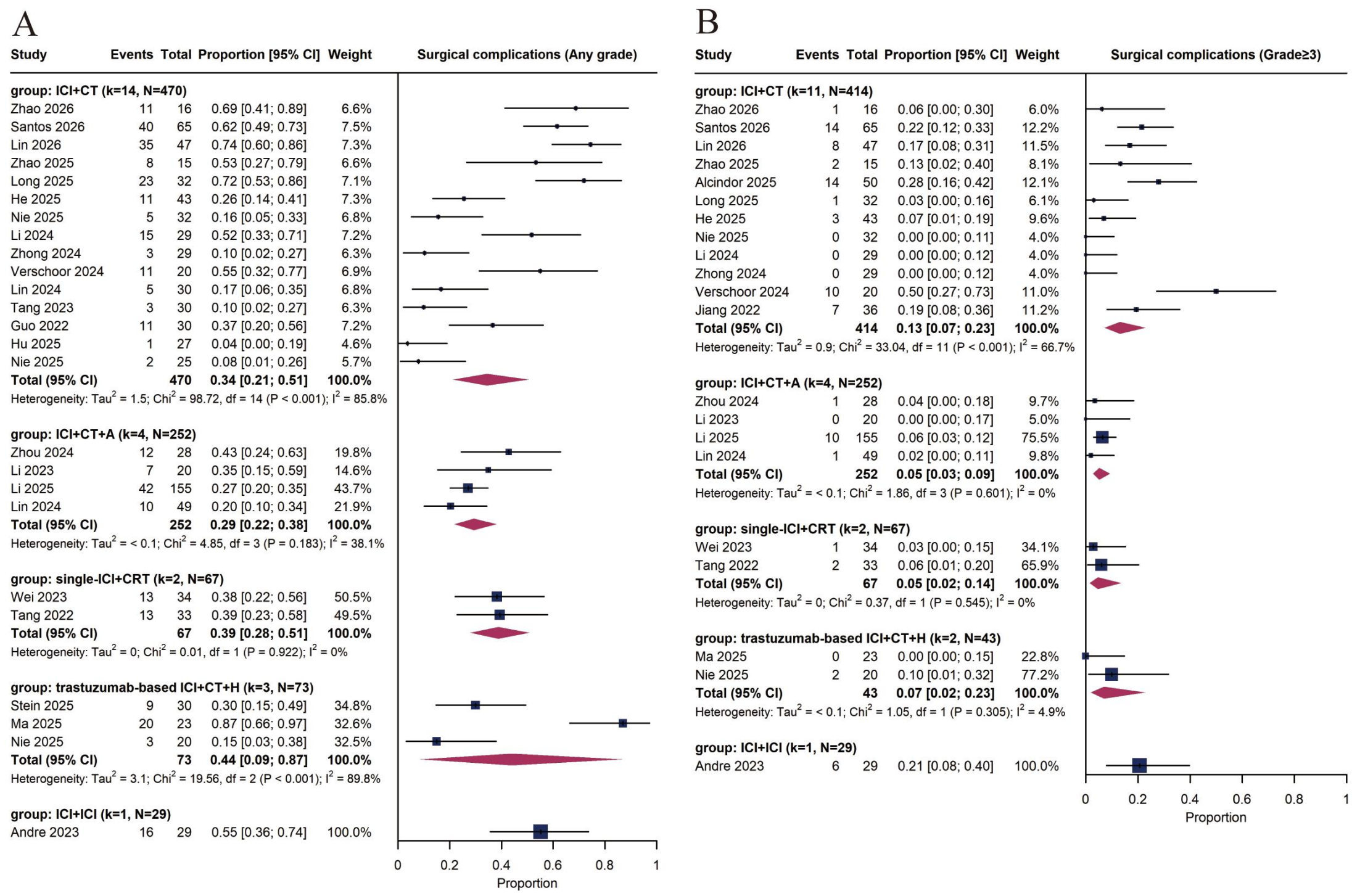

eFigure 22. Leave-One-Out Sensitivity Analyses of Pooled Risk Ratios for pCR (A) and MPR (B) for ICI+CT Versus Chemotherapy

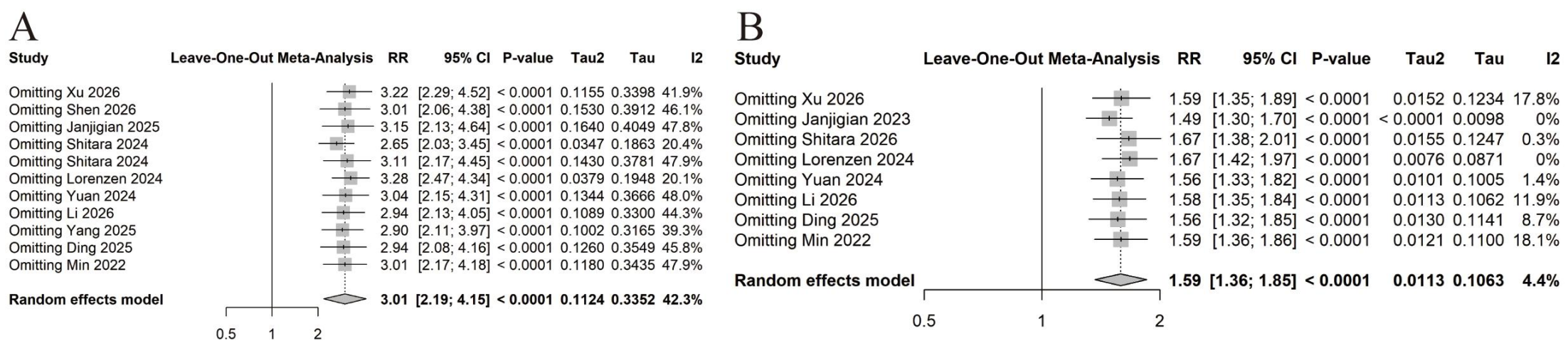

eFigure 23. Leave-One-Out Sensitivity Analysis of Pooled pCR Rates for Treatment Strategy: ICI+CT (A), ICI+CT+A (B), Single-ICI+CRT (C), Disitamab Vedotin-based ICI+CT+H (D), and Trastuzumab-based ICI+CT+H (E)

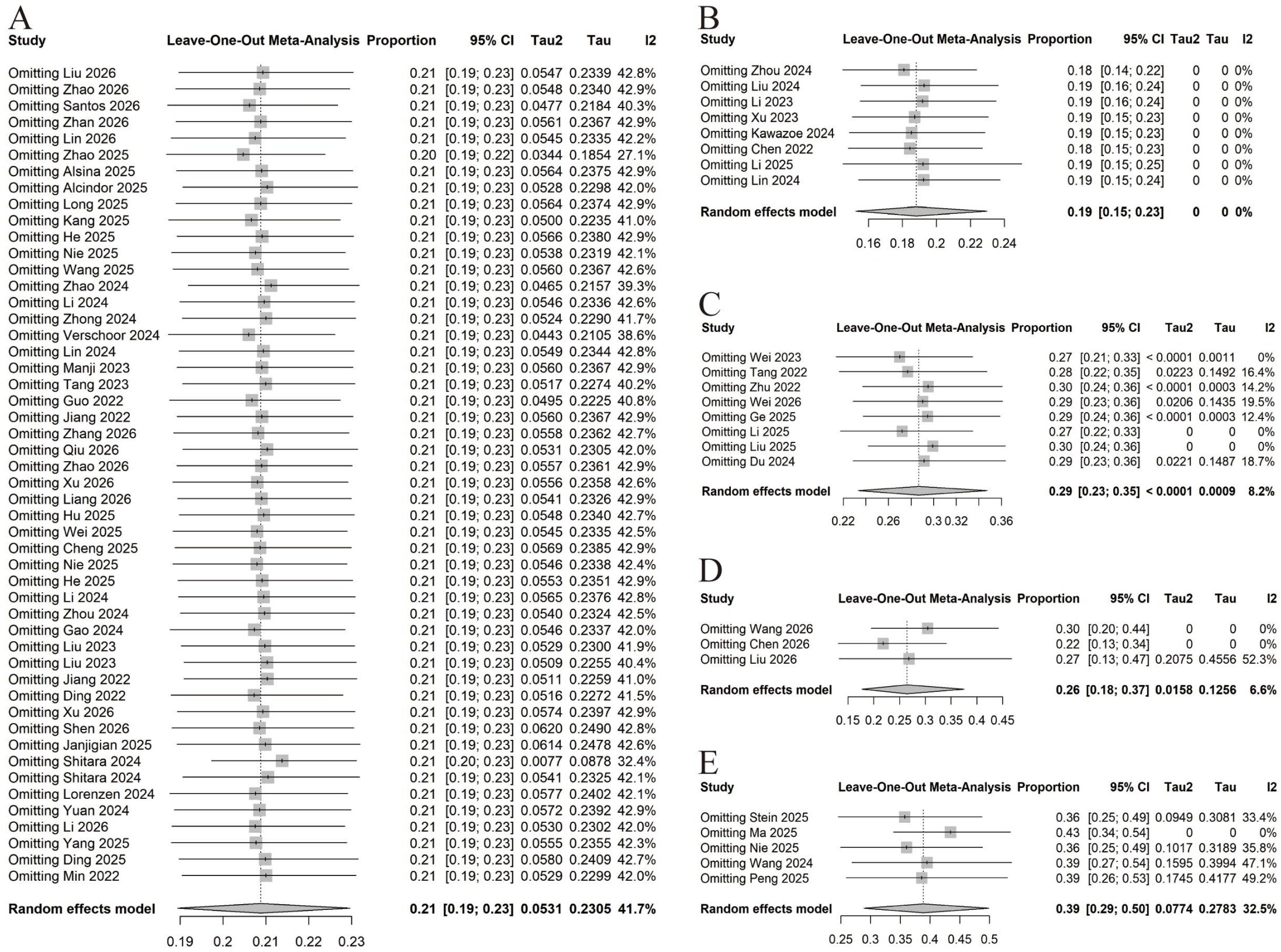

eFigure 24. Leave-One-Out Sensitivity Analysis of Pooled MPR Rates for Treatment Strategy: ICI+CT (A), ICI+CT+A (B), Single-ICI+CRT (C), Disitamab Vedotin-based ICI+CT+H (D), and Trastuzumab-based ICI+CT+H (E)

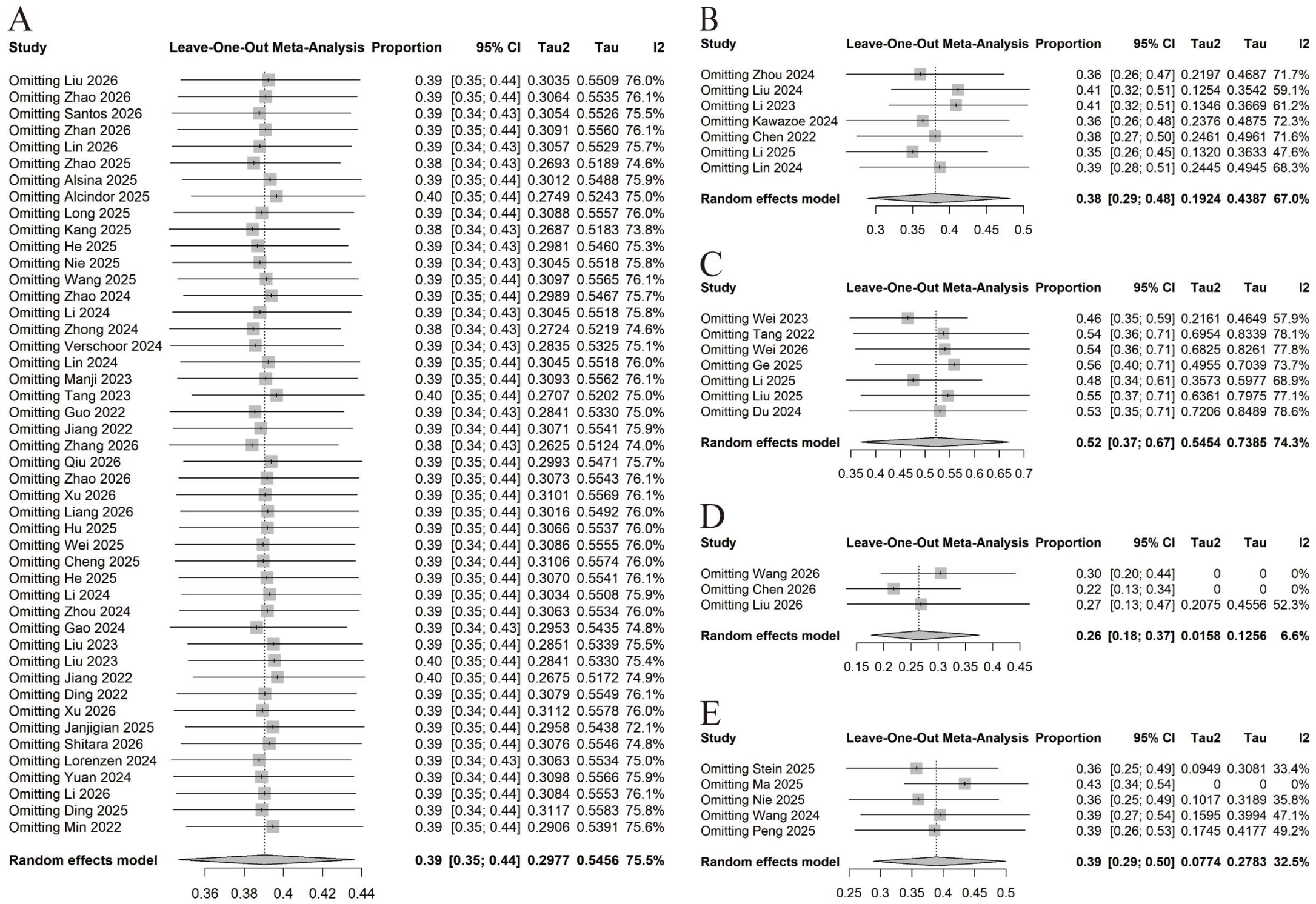

eFigure 25. Funnel Plot for Publication Bias Assessment of pCR in the ICI+CT Versus Chemotherapy Comparison

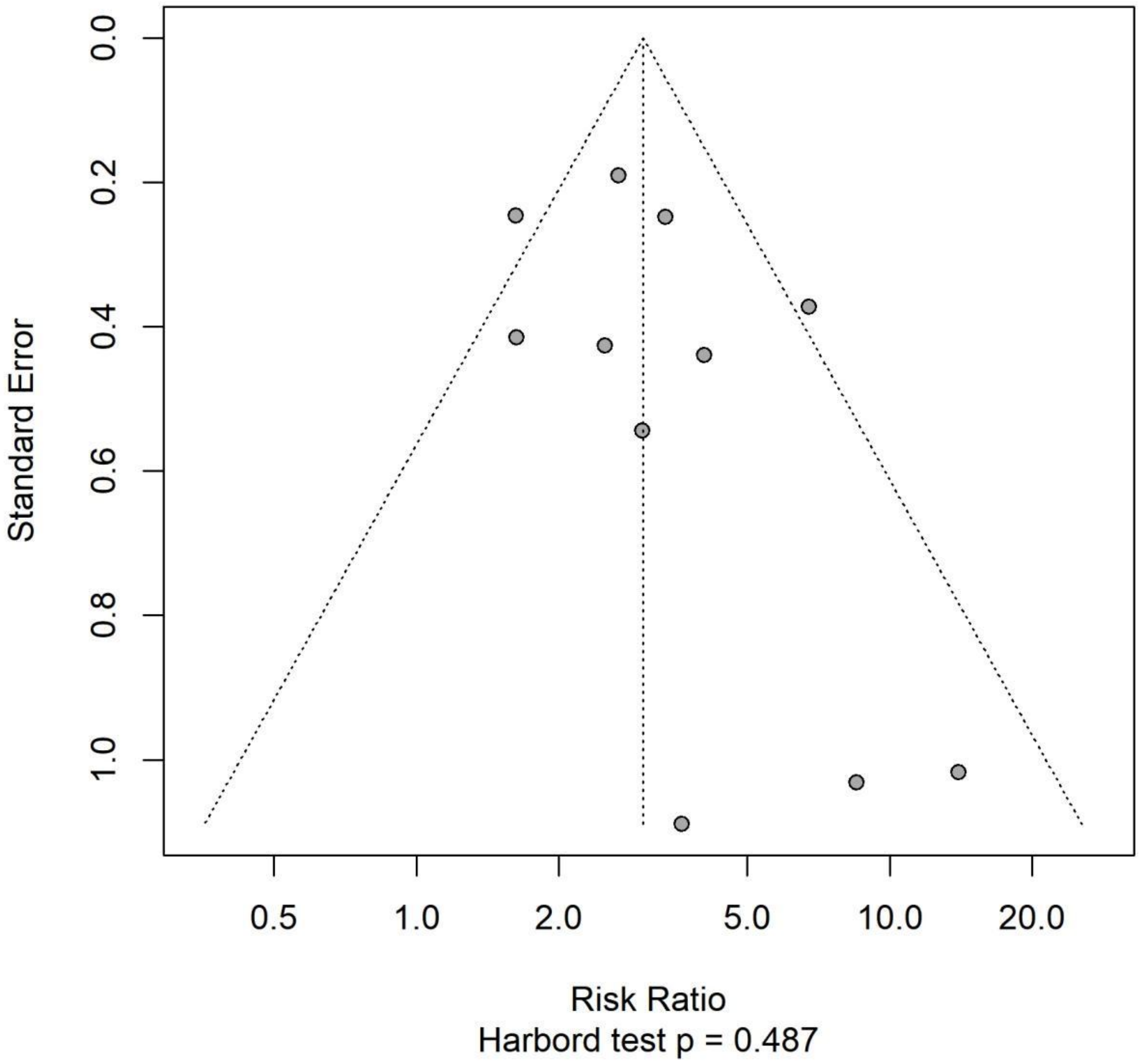

eTable 1. Differences between protocol and review

Domain	PROSPERO registration	Final review	Rationale
**Scope and treatment terminology**	The registration used the term "ICI-based neoadjuvant therapy" and defined eligible interventions according to treatment administered before surgery.	Eligible treatment was described as ICI-based neoadjuvant/perioperative therapy initiated before surgery. This included neoadjuvant-only regimens and perioperative regimens in which treatment continued after surgery. Adjuvant-only ICI initiated after surgery remained ineligible.	At registration, "neoadjuvant" was used to describe regimens containing a preoperative ICI component. The revised terminology more accurately reflects total treatment exposure in trials that continued treatment after surgery. This was a clarification of terminology rather than an expansion of eligibility because all included regimens initiated ICI before surgery, and no study became eligible solely because of postoperative treatment.
**language restrictions**	Eligibility was initially restricted to English-language reports.	The final search was conducted without language restrictions.	The language restriction was removed to reduce potential language bias and increase the comprehensiveness of the search.
**Effect measures**	ORs were planned for dichotomous outcomes, whereas HRs were planned for time-to-event outcomes.	RRs were used for dichotomous comparative outcomes and HRs for time-to-event outcomes.	RRs were considered more directly interpretable than ORs for clinical outcomes with relatively frequent events, such as pathological response and surgical resection. The selected effect measure was applied consistently across comparative analyses.
**Meta-analysis model**	Fixed- or random-effects models were to be selected according to the observed statistical heterogeneity.	REML random-effects models were applied consistently. Single-arm proportions were pooled using inverse-variance random-effects models with logit transformation.	Clinical and methodological heterogeneity was anticipated because of differences in treatment regimens, populations, and trial characteristics. A random-effects framework was therefore considered more appropriate than selecting the model according to the observed I^2 value. REML was used to estimate between-study variance.
**Reporting-bias assessment**	Risk of bias due to missing results was not planned to be assessed.	Publication bias was assessed only for strategy-specific pairwise analyses with at least 10	Reporting-bias assessment was added to provide a more complete evaluation of evidence robustness. The assessment

		unique trials, using funnel-plot inspection and Harbord's test for binary outcomes. Trim-and-fill analysis was reserved for exploratory use when asymmetry was suspected.	was restricted to analyses with at least 10 trials because funnel plots and statistical tests are unreliable when fewer studies are available.
**Outcomes**	Prespecified outcomes included pCR, MPR, R0 resection, severe TRAEs, EFS, DFS, OS, PFS, ORR, DCR, and postoperative complications.	Surgical resection rate and irAEs were additionally evaluated. TRAEs and irAEs were analyzed by severity and separately for source-reported neoadjuvant and perioperative periods. DFS was not synthesized separately because compatible extractable DFS data were unavailable.	Surgical resection and immune-related toxicity were clinically relevant and sufficiently reported in the available studies. Separating safety outcomes by severity and treatment period accounted for differences in treatment exposure.
**Subgroup analyses**	Subgroup analyses were planned according to treatment regimen, study design, PD-L1 status, MSI status, Lauren classification, Borrmann classification, tumor location, and TMB, subject to data availability.	Comparative analyses were conducted separately by treatment strategy. Exploratory subgroup analyses of ICI+CT versus chemotherapy evaluated PD-L1 status, MMR status, Lauren classification, T stage, tumor location, ECOG performance status, sex, age, and region when sufficient data were available. Borrmann classification and TMB analyses were not performed.	Strategy-specific analyses were used to avoid treating clinically heterogeneous regimens as a common treatment effect. Subgroup factors were evaluated according to clinical relevance and data availability. Borrmann classification and TMB were not analyzed because sufficiently comparable data were unavailable.
**Sensitivity analyses and analysis populations**	Specific sensitivity analyses and the primary analysis population were not prespecified.	ITT analyses were considered primary, with PP analyses performed when available. Additional analyses included leave-one-out analyses and exclusion of abstract-derived outcomes, high-risk-of-bias trials, and biomarker-enriched cohorts.	These analyses were undertaken to evaluate whether the results were sensitive to analysis population, publication format, risk of bias, individual influential studies, or biomarker enrichment. They were performed only when at least two eligible studies remained.
**Review team**	The registration listed Zhengyi Chen and Junlin Zhou as review team	The final author group included Zhengyi Chen, Xin Liu, Hanling Sun, Nini Cao, Junlin Zhou, Zihan	The team was expanded to support independent screening, data extraction and verification, statistical analysis, clinical

	members.	Chen, Yang Song, and Jun Yao. Individual roles are reported in the Author Contributions section.	interpretation, and methodological and senior supervision.
**Funding**	The registration stated that the review had no funding or agreed institutional support.	The final manuscript reports support from the Technical Research and Development Project of Shenzhen (JCYJ20240813104413019).	Funding information was updated to ensure complete disclosure. The funder had no role in study selection, data extraction, statistical analysis, interpretation of the results, or the decision to submit the manuscript.
**Review timeline**	The planned review period was December 23, 2025, to February 28, 2026.	The review continued beyond the initially planned completion date, and the literature search was updated through August 3, 2026.	The timeline was extended because of the rapidly evolving evidence base, the publication of additional randomized and prospective studies, and the need to update the search and analyses before completion of the review.

eTable 2. Search Strategy for PubMed

Step	Search strategy	Numbers
**1**	("Immune Checkpoint Inhibitors"[Mesh]) OR ((((((((((((((((((((((((((((((((((Immune Checkpoint Inhibitors) OR (Checkpoint Inhibitors, Immune)) OR (Immune Checkpoint Inhibitor)) OR (Checkpoint Inhibitor, Immune)) OR (Immune Checkpoint Blockers)) OR (Checkpoint Blockers, Immune)) OR (Immune Checkpoint Blockade)) OR (Checkpoint Blockade, Immune)) OR (Immune Checkpoint Inhibition)) OR (Checkpoint Inhibition, Immune)) OR (PD-L1 Inhibitors)) OR (PD L1 Inhibitors)) OR (PD-L1 Inhibitor)) OR (PD L1 Inhibitor)) OR (Programmed Death-Ligand 1 Inhibitors)) OR (Programmed Death Ligand 1 Inhibitors)) OR (PD-1-PD-L1 Blockade)) OR (Blockade, PD-1-PD-L1)) OR (PD 1 PD L1 Blockade)) OR (CTLA-4 Inhibitors)) OR (CTLA 4 Inhibitors)) OR (CTLA-4 Inhibitor)) OR (CTLA 4 Inhibitor)) OR (Cytotoxic T-Lymphocyte-Associated Protein 4 Inhibitors)) OR (Cytotoxic T Lymphocyte Associated Protein 4 Inhibitors)) OR (Cytotoxic T-Lymphocyte-Associated Protein 4 Inhibitor)) OR (Cytotoxic T Lymphocyte Associated Protein 4 Inhibitor)) OR (PD-1 Inhibitors)) OR (PD 1 Inhibitors)) OR (PD-1 Inhibitor)) OR (Inhibitor, PD-1)) OR (PD 1 Inhibitor)) OR (Programmed Cell Death Protein 1 Inhibitor)) OR (Programmed Cell Death Protein 1 Inhibitors))	88222
**2**	(((((((((((((((((((Tislelizumab) OR (Toripalimab)) OR (Avelumab)) OR (Cadonilimab)) OR (Durvalumab)) OR (Camrelizumab)) OR (Sintilimab)) OR (Atezolizumab)) OR (Pembrolizumab)) OR (Nivolumab)) OR (Serplulimab)) OR (Adebrelimab)) OR (Spartalizumab)) OR (Cemiplimab)) OR (Dostarlimab)) OR (Zimberelimab)) OR (Penpulimab)) OR (Sugemalimab)) OR (Envafolimab))	34690
**3**	1 OR 2	98350
**4**	((neoadjuvant) OR (neo-adjuvant)) OR (perioperative)	294804
**5**	(((stomach) OR (gastric)) OR (Esophagogastric)) OR (Gastroesophageal)	540848
**6**	3 AND 4 AND 5	484

eTable 3. Search Strategy for Web of Science

Step	Search strategy	Numbers
**1**	((((((((ALL=(Immune Checkpoint Inhibitor)) OR ALL=(Immune Checkpoint Blockers)) OR ALL=(CTLA-4 Inhibitors)) OR ALL=(Cytotoxic T-Lymphocyte-Associated Protein 4 Inhibitors)) OR ALL=(PD-1)) OR ALL=(programmed cell death 1)) OR ALL=(PD-L1)) OR ALL=(Programmed Death Ligand 1)) OR ALL=(ICI)	165469
**2**	((((((((((((((((((ALL=(Tislelizumab)) OR ALL=(Toripalimab)) OR ALL=(Avelumab)) OR ALL=(Cadonilimab)) OR ALL=(Durvalumab)) OR ALL=(Camrelizumab)) OR ALL=(Sintilimab)) OR ALL=(Atezolizumab)) OR ALL=(Pembrolizumab)) OR ALL=(Durvalumab)) OR ALL=(Nivolumab)) OR ALL=(serplulimab)) OR ALL=(Adebrelimab)) OR ALL=(Spartalizumab)) OR ALL=(Cemiplimab)) OR ALL=(Dostarlimab)) OR ALL=(Zimberelimab)) OR ALL=(Penpulimab)) OR ALL=(Sugemalimab)) OR ALL=(Envafolimab)	63422
**3**	#1 OR #2	197072
**4**	((ALL=(neoadjuvant)) OR ALL=(neo-adjuvant)) OR ALL=(perioperative)	227671
**5**	(((ALL=(stomach)) OR ALL=(gastric)) OR ALL=(Esophagogastric)) OR ALL=(Gastroesophageal)	404309
**6**	#3 AND #4 AND #5	748

eTable 4. Search Strategy for Embase

Step	Search strategy	Numbers
**1**	'immune checkpoint inhibitor'/exp	62101
**2**	'immune checkpoint inhibitor':ti,ab,kw OR 'immune checkpoint blocker':ti,ab,kw OR 'immune checkpoint inhibitors':ti,ab,kw	71977
**3**	'cytotoxic t lymphocyte antigen 4'/exp	41314
**4**	'antigen cd152':ti,ab,kw OR 'cd152 antigen':ti,ab,kw OR 'ctla 4':ti,ab,kw OR 'ctla 4 antigen':ti,ab,kw OR 'ctla-4 antigen':ti,ab,kw OR ctla4:ti,ab,kw OR 'cytotoxic t lymphocyte associated antigen 4':ti,ab,kw OR 'cytotoxic t lymphocyte antigen 4':ti,ab,kw	35158
**5**	'programmed death 1 ligand 1'/exp	91502
**6**	'programmed cell death 1 ligand 1':ti,ab,kw OR 'programmed death 1 ligand 1 protein':ti,ab,kw OR 'programmed death ligand 1':ti,ab,kw OR 'programmed death 1 ligand 1':ti,ab,kw OR 'pd l1':ti,ab,kw	84114
**7**	'programmed cell death 1':ti,ab,kw OR 'pd 1':ti,ab,kw	90013
**8**	#1 OR #2 OR #3 OR #4 OR #5 OR #6 OR #7	233898
**9**	tislelizumab:ti,ab,kw OR toripalimab:ti,ab,kw OR avelumab:ti,ab,kw OR cadonilimab:ti,ab,kw OR camrelizumab:ti,ab,kw OR sintilimab:ti,ab,kw OR atezolizumab:ti,ab,kw OR pembrolizumab:ti,ab,kw OR durvalumab:ti,ab,kw OR nivolumab:ti,ab,kw OR serplulimab:ti,ab,kw OR adebrelimab:ti,ab,kw OR spartalizumab:ti,ab,kw OR cemiplimab:ti,ab,kw OR dostarlimab:ti,ab,kw OR zimberelimab:ti,ab,kw OR penpulimab:ti,ab,kw OR sugemalimab:ti,ab,kw OR envafolimab:ti,ab,kw	84182
**10**	#8 OR #9	266338
**11**	neoadjuvant:ti,ab,kw OR 'neo adjuvant':ti,ab,kw OR perioperative:ti,ab,kw	392404
**12**	stomach:ti,ab,kw OR gastric:ti,ab,kw OR esophagogastric:ti,ab,kw OR gastroesophageal:ti,ab,kw	645100
**13**	#10 AND #11 AND #12	1287

eTable 5. Search Strategy for the Cochrane Library

Step	Search strategy	Numbers
**1**	(Immune Checkpoint Inhibitors):ti,ab,kw OR (Cytotoxic T-Lymphocyte-Associated Protein 4 Inhibitor):ti,ab,kw OR (Cytotoxic T Lymphocyte Associated Protein 4 Inhibitors):ti,ab,kw OR (CTLA 4 Inhibitors):ti,ab,kw OR (CTLA-4 Inhibitor):ti,ab,kw OR (CTLA 4 Inhibitor):ti,ab,kw OR (Cytotoxic T-Lymphocyte-Associated Protein 4 Inhibitors):ti,ab,kw OR (CTLA-4 Inhibitors):ti,ab,kw OR (Cytotoxic T Lymphocyte Associated Protein 4 Inhibitor):ti,ab,kw OR (Programmed Cell Death Protein 1 Inhibitors):ti,ab,kw OR (PD-1 Inhibitor):ti,ab,kw OR (Inhibitor, PD-1):ti,ab,kw OR (PD 1 Inhibitors):ti,ab,kw OR (PD-1 Inhibitors):ti,ab,kw OR (Programmed Cell Death Protein 1 Inhibitor):ti,ab,kw OR (PD 1 Inhibitor):ti,ab,kw OR (Checkpoint Inhibitors, Immune):ti,ab,kw OR (Immune Checkpoint Inhibitor):ti,ab,kw OR (Checkpoint Inhibitor, Immune):ti,ab,kw OR (Immune Checkpoint Blockers):ti,ab,kw OR (Checkpoint Blockers, Immune):ti,ab,kw OR (PD 1 PD L1 Blockade):ti,ab,kw OR (Immune Checkpoint Blockade):ti,ab,kw OR (Checkpoint Blockade, Immune):ti,ab,kw OR (Immune Checkpoint Inhibition):ti,ab,kw OR (Checkpoint Inhibition, Immune):ti,ab,kw OR (PD-L1 Inhibitors):ti,ab,kw OR (PD L1 Inhibitors):ti,ab,kw OR (Programmed Death-Ligand 1 Inhibitors):ti,ab,kw OR (PD-L1 Inhibitor):ti,ab,kw OR (Programmed Death Ligand 1 Inhibitors):ti,ab,kw OR (PD L1 Inhibitor):ti,ab,kw	10789
**2**	(Tislelizumab):ti,ab,kw OR (Toripalimab):ti,ab,kw OR (Avelumab):ti,ab,kw OR (Cadonilimab):ti,ab,kw OR (Durvalumab):ti,ab,kw OR (Camrelizumab):ti,ab,kw OR (Sintilimab):ti,ab,kw OR (Atezolizumab):ti,ab,kw OR (Pembrolizumab):ti,ab,kw OR (Nivolumab):ti,ab,kw OR (Serplulimab):ti,ab,kw OR (Adebrelimab):ti,ab,kw OR (Spartalizumab):ti,ab,kw OR (Cemiplimab):ti,ab,kw OR (Dostarlimab):ti,ab,kw OR (Zimberelimab):ti,ab,kw OR (Penpulimab):ti,ab,kw OR (Sugemalimab):ti,ab,kw OR (Envafolimab):ti,ab,kw	14761
**3**	#1 OR #2	21313
**4**	(neoadjuvant):ti,ab,kw OR (neo adjuvant):ti,ab,kw OR (perioperative):ti,ab,kw	48705
**5**	(stomach):ti,ab,kw OR (gastric):ti,ab,kw OR (esophagogastric):ti,ab,kw OR (Gastroesophageal):ti,ab,kw	49693
**6**	#3 AND #4 AND #5	258

eTable 6. Report-to-trial linkage and handling of multiple reports

Trial Name	Registration No.	Included Reports (Author, Year, Publication Type)	Data Source for Primary Meta-Analysis	Patient Overlap Declaration
MATTERHORN	NCT04592913	1. Janjigian 2023 (9) (Abstract)<br>2. Janjigian 2025 (10) (Full text)<br>3. Tabernero 2025 (6) (Abstract)	1. Janjigian 2023: MPR<br>2. Janjigian 2025: pCR, EFS, surgical resection rate, R0 resection rate, safety<br>3. Tabernero 2025: EFS of MPR subgroup, final OS	All three reports derive from the same randomised phase III trial and the same patient population. Janjigian 2023 provided preliminary efficacy data, whereas Janjigian 2025 reported the primary and key secondary endpoints with complete overlap of patients relative to the 2023 report. For this meta-analysis, Janjigian 2023 contributed MPR only (an outcome not reported in the 2025 full text), while all other endpoints were extracted from Janjigian 2025. Tabernero 2025 reported final OS and the association of pathological outcomes with EFS (including MPR-subgroup EFS) in the identical patient population; it was used solely for updated survival endpoints. No single outcome was extracted from more than one source, and no participant was counted twice.
KEYNOTE-585	NCT03221426	1. Al-Batran 2024 (13) (Abstract)<br>2. Shitara 2024 (11) (Full text)<br>3. Shitara 2025 (5) (Full text)<br>4. Shitara 2026 (12) (Full text)	1. Al-Batran 2024: pCR, R0 resection rate for FLOT Cohort<br>2. Shitara 2024: pCR, R0 resection rate, safety for Main Cohort; safety for FLOT Cohort<br>3. Shitara 2025: OS, EFS for Main + FLOT; surgical resection rate for Main Cohort; surgical resection rate for FLOT Cohort<br>4. Shitara 2026: MPR, EFS of MPR subgroup for Main + FLOT	KEYNOTE-585 comprises two mutually exclusive cohorts (Main Cohort receiving cisplatin-based doublet and FLOT Cohort receiving FLOT), with no patient overlap between them. For this meta-analysis, cohort-specific data were extracted and the two cohorts were pooled separately as independent entries whenever possible: pCR and R0 resection rate were derived from Al-Batran 2024 (FLOT) and Shitara 2024 (Main); safety data from Shitara 2024 (both cohorts); and surgical resection rate from Shitara 2025 (both cohorts). For outcomes where only pooled estimates were available, the combined Main plus FLOT Cohort data were used: OS and EFS from Shitara 2025, and MPR together with EFS of the MPR subgroup from Shitara 2026. No participant was counted twice for any single endpoint.
NEOSUMMIT-01	NCT04250948	1. Yuan 2024 (15) (Full text)	1. Yuan 2024: pCR, MPR, surgical resection rate, R0 resection rate, safety	Complete overlap: both reports describe the same patient population from the same trial. Different outcomes were extracted from each report, ensuring no

		2. Nie 2026 (16) (Full text)	2. Nie 2026: OS, EFS	double-counting.
Arise-FJ-G 005	NCT041 95828	1. Lin 2024 (22) (Full text)<br>2. Lin 2025 (23) (Full text)	1. Lin 2024: pCR, MPR, surgical resection rate, R0 resection rate, neoadjuvant safety<br>2. Lin 2025: OS, PFS, perioperative safety	Complete overlap: both reports describe the same patient population. Different outcomes were extracted from each report, ensuring no double-counting.
NICE	NCT047 44649	1. Zhao 2026 (29) (Full text)<br>2. Zhao 2025 (33) (Full text)	1. Zhao 2026: pCR, MPR, R0, safety, EFS, OS for Cohort B (EBV-associated)<br>2. Zhao 2025: pCR, MPR, R0, safety, EFS, OS for Cohort C (dMMR/MSI-H)	The NICE trial enrolled patients into three biomarker-defined cohorts; Cohort A (PD-L1 CPS ≥5) has not yet been published and was not included. The two included reports describe mutually exclusive cohorts: Cohort B (EBV-associated, n=17) and Cohort C (dMMR/MSI-H, n=16). Patients were assigned to cohorts based on distinct molecular profiles with no overlap between cohorts. Each cohort was analyzed as a separate entry in the meta-analysis. No participant was counted twice.

NOTE: The remaining 69 studies each corresponded to one report, with no duplicate counting of participants.

eTable 7. Basic Characteristics of Studies Included in the Single-arm Meta-analysis

Group	Study	Registration No.	Study type	Phase	Country	Sample size	Age	Proportion of males (%)	Proportion of cT4 (%)	Neoadjuvant therapy	Adjuvant therapy
**ICI+CT**	Liu 2026 (28)	NCT06198465	single-arm	II	China	24	62 (51-75)	83.3	41.7	Adebrelimab+Chemo (nab-paclitaxel+lobaplatin+S-1)	At the investigator's discretion (regimen not prespecified)
**ICI+CT**	Zhao 2026 (29)	NCT04744649	single-arm	II	China	17	59.4 (40-77)	94.1	64.7	Toripalimab+Chemo (CapeOX)	Toripalimab+Chemo (CapeOX)
**ICI+CT**	Santos 2026 (30)	NCT04736485	single-arm	II	France	67	63 (31-79)	77.6	0	Spartalizumab+Chemo (FLOT)	Spartalizumab+Chemo (FLOT)
**ICI+CT**	Zhan 2026 (31)	NCT06496789	single-arm	II	China	33	60 (33-75)	78.8	97	Serplulimab+Chemo (SOX)	Serplulimab+Chemo (SOX)
**ICI+CT**	Lin 2026 (32)	NCT04890392	single-arm	II	China	49	58.5 (34-74)	79.6	42.9	Tislelizumab+Chemo (SOX)	Chemo (SOX)
**ICI+CT**	Zhao 2025 (33)	NCT04744649	single-arm	II	China	16	68.5 (51-73)	68.8	75	Toripalimab+Chemo (CapeOX)	Toripalimab+Chemo (CapeOX)
**ICI+CT**	Alsina 2025 (34)	NCT03979131	single-arm	II	Spain	40	64 (56-70)	55	25	Avelumab+Chemo (FLOT)	Avelumab+Chemo (FLOT)
**ICI+CT**	Alcindor 2025 (35)	NCT03288350	single-arm	II	Canada	51	64 (18-79)	88.2	5.9	Avelumab+Chemo (DCF)	Avelumab+Chemo (DCF)
**ICI+CT**	Long 2025 (36)	ChiCTR2200066893	single-arm	II	China	38	57 (38-74)	89.5	60.5	Cadonilimab+Chemo (FLOT)	Chemo (FLOT)
**ICI+CT**	Kang 2025 (37)	04221555	single-arm	II	Korea	50	63 (39-83)	72	76	Durvalumab+Chemo (DOS)	Durvalumab+Chemo (S-1)

**ICI+CT**	He 2025 (38)	NCT05715632	single-arm	II	China	46	54 (44-64)	78.3	34.8	Camrelizumab+Chemo (XELOX)	Camrelizumab+Chemo (XELOX)
**ICI+CT**	Nie 2025 (39)	ChiCTR2200061906	single-arm	II	China	32	60 (28-74)	78.1	37.5	Tislelizumab+Chemo (SOX)	Tislelizumab+Chemo (SOX)
**ICI+CT**	Wang 2025 (40)	NCT05515796	single-arm	II	China	45	65 (55-69)	/	/	Tislelizumab+Chemo (XELOX)	Postoperative therapy planned; regimen not reported
**ICI+CT**	Zhao 2024 (41)	NCT03939962	single-arm	II	China	60	58 (29-72)	71.7	46.7	Camrelizumab+Chemo (mFOLFOX6)	Camrelizumab+Chemo (mFOLFOX6)
**ICI+CT**	Li 2024 (42)	NCT04341857	single-arm	II	China	32	58 (43-70)	78.1	59.4	Sintilimab+Chemo (FLOT)	Chemo (FLOT)
**ICI+CT**	Zhong 2024 (43)	NCT05602935	single-arm	II	China	29	62 (39-84)	62.1	58.6	Camrelizumab+Chemo (SOX)	Camrelizumab+Chemo (SOX)
**ICI+CT**	Verschoor 2024 (44)	NCT03448835	single-arm	II	Netherlands	21	62 (46-76)	90.5	9.5	Atezolizumab+Chemo (DOC)	None
**ICI+CT**	Lin 2024 (45)	ChiCTR2200055659	single-arm	II	China	30	59 (47-71)	63.3	83.3	Sintilimab+Chemo (SOX+albumin paclitaxel)	Chemo (SOX+albumin paclitaxel)
**ICI+CT**	Manji 2023 (46)	NCT02918162	single-arm	II	United States	36	/	/	/	Pembrolizumab+Chemo (CapeOX±Epirubicin)	Pembrolizumab+Chemo (CapeOX)
**ICI+CT**	Tang 2023 (47)	NCT04755543	single-arm	I	China	30	64.5 (50-74)	86.7	16.7	LP002+Chemo (FP)	LP002+Chemo (FP)
**ICI+CT**	Guo 2022 (48)	ChiCTR2000030414	single-arm	II	China	30	62 (30-72)	60	73.3	Sintilimab+Chemo (XELOX)	Chemo (XELOX)
**ICI+CT**	Jiang 2022 (49)	NCT04065282	single-arm	II	China	36	65.5 (43-76)	66.7	69.4	Sintilimab+Chemo (CapeOX)	Chemo (CapeOX)
**ICI+CT**	Zhang	NCT05948449	single-	II	China	37	58	/	70.3	Cadonilimab+Chemo	Not reported

	2026 (50)		arm (Abstract)				(27-72)			(SOX)	
**ICI+CT**	Qiu 2026 (51)	NCT06451211	single-arm (Abstract)	II	China	56	58 (/-/)	/	/	Tislelizumab+Chemo (CapeOX/SOX)	Not reported
**ICI+CT**	Zhao 2026 (52)	NCT06310473	single-arm (Abstract)	II	China	30	/	/	83.3	Cadonilimab+Chemo (CapeOX)	Chemo (CapeOX)
**ICI+CT**	Xu 2026 (53)	ChiCTR2300075638	single-arm (Abstract)	II	China	40	66 (37-76)	87.5	/	Tislelizumab+Chemo (SOX)	Not reported
**ICI+CT**	Liang 2026 (54)	ChiCTR2400082697	single-arm (Abstract)	II	China	11	/	/	/	Adebrelimab+Chemo (SOX+nab-paclitaxel)	Not reported
**ICI+CT**	Hu 2025 (55)	/	single-arm (Abstract)	II	China	31	/	/	/	Tislelizumab+Chemo (SOX)	Not reported
**ICI+CT**	Wei 2025 (56)	ChiCTR2500114611	single-arm (Abstr	II	China	26	/	/	/	Sintilimab+Chemo (XELOX)	Not reported

			act)								
**ICI+CT**	Cheng 2025 (57)	NCT05739045	single-arm (Abstract)	II	China	46	66 (34-74)	82.6	/	Nivolumab+Chemo (SOX)	Nivolumab+Chemo (SOX)
**ICI+CT**	Nie 2025 (58)	ChiCTR2300069000	single-arm (Abstract)	II	China	30	/	/	/	Serplulimab+Chemo (SOX)	Not reported
**ICI+CT**	He 2025 (59)	NCT06475417	single-arm (Abstract)	II	China	27	61 (39-71)	/	70.4	Adebrelimab+Chemo (DOS)	Not reported
**ICI+CT**	Li 2024 (60)	NCT04354662	single-arm (Abstract)	II	China	55	57 (28-76)	/	/	Toripalimab+Chemo (FLOT)	Toripalimab+Chemo (FLOT)
**ICI+CT**	Zhou 2024 (61)	NCT05507658/NCT05508399	single-arm (Abstract)	II	China	28	59.5 (± 8.2)	67.9	/	Tislelizumab+Chemo (XELOX)	Tislelizumab+Chemo (XELOX)
**ICI+CT**	Gao 2024 (26)	NCT04891016	RCT (Abstract)	II	China	69	/	/	/	Toripalimab+Chemo (FLOT)	Not reported
**ICI+CT**	Liu 2023 (27)	NCT04908566	RCT (Abstr	II	China	21	/	/	/	Toripalimab+Chemo (FOLFIRINOX)	Toripalimab+Chemo (FOLFIRINOX)

			act)								
**ICI+CT**	Liu 2023 (27)	NCT04908566	RCT (Abstract)	II	China	33	/	/	/	Toripalimab+Chemo (SOX)	Toripalimab+Chemo (SOX)
**ICI+CT**	Jiang 2022 (62)	NCT04119622	single-arm (Abstract)	II	China	35	61 (34-72)	80	/	Toripalimab+Chemo (XELOX)	Toripalimab+Chemo (XELOX)
**ICI+CT**	Ding 2022 (63)	ChiCTR2100043572	single-arm (Abstract)	II	China	21	56 (31-72)	47.6	90.5	Sintilimab+Chemo (SOX)	Sintilimab+Chemo (S-1)
**ICI+CT**	Xu 2026 (7)	NCT06374901	RCT	II	China	68	/	76.5	82.4	Tislelizumab+Chemo (SOX/XELOX)	Tislelizumab+Chemo (SOX/XELOX)
**ICI+CT**	Shen 2026 (8)	NCT04139135	RCT	III	China	292	61.5 (55-66)	80.1	60.6	Serplulimab+Chemo (SOX)	Serplulimab
**ICI+CT**	Janjigian 2023 (9), Janjigian 2025 (10), Tabernero 2025 (6)	NCT04592913	RCT	III	United States	474	62 (26-84)	68.8	24.7	Durvalumab+Chemo (FLOT)	Durvalumab+Chemo (FLOT)
**ICI+CT**	Shitara 2024 (11), Shitara	NCT03221426	RCT	III	Japan	402	64 (56-70)	71.6	41	Pembrolizumab+Chemo (XP/FP)	Pembrolizumab+Chemo (XP/FP)

	2025 (5), Shitara 2026 (12)										
**ICI+CT**	Al-Batran 2024 (13), Shitara 2024 (11), Shitara 2025 (5)	NCT03221426	RCT	III	Japan	100	61.5 (54.5-69)	78	/	Pembrolizumab+Chemo (FLOT)	Pembrolizumab+Chemo (FLOT)
**ICI+CT**	Lorenzen 2024 (14)	NCT03421288	RCT	II/III	Germany	146	61 (29-79)	79.5	5	Atezolizumab+Chemo (FLOT)	Atezolizumab+Chemo (FLOT)
**ICI+CT**	Yuan 2024 (15), Nie 2026 (16)	NCT04250948	RCT	II	China	54	58 (48-67)	64.8	61.1	Toripalimab+Chemo (SOX/XELOX)	Toripalimab+Chemo (SOX/XELOX)
**ICI+CT**	Li 2026 (17)	NCT06576921	RCT (Abstract)	II	China	23	/	/	/	Serplulimab+Chemo (SOX+nab-paclitaxel)	Serplulimab+Chemo (SOX+nab-paclitaxel)
**ICI+CT**	Yang 2025 (18)	NCT06192186	RCT (Abstract)	II	China	55	/	/	/	Adebrelimab+Chemo (SOX)	Determined according to postoperative pathological findings
**ICI+CT**	Ding 2025 (19)	NCT04982939	RCT (Abstract)	II	China	131	/	/	/	Sintilimab+Chemo (SOX)	Sintilimab+Chemo (SOX)
**ICI+CT**	Min 2022 (20)	ChiCTR2000030610	RCT (Abstr	II	China	33	/	/	/	Camrelizumab+Chemo (FLOT)	Not reported

			act)								
**ICI+CT+A**	Zhou 2024 (64)	ChiCTR2200055269	single-arm	II	China	30	58 (35-76)	86.7	50	Sintilimab+Apatinib+Chemo (FLOT)	Chemo (FLOT)
**ICI+CT+A**	Liu 2024 (65)	/	single-arm	/	China	32	68 (/-/)	59.4	56.3	Penpulimab+Anlotinib+Chemo (SOX)	Penpulimab+Anlotinib+Chemo (SOX)
**ICI+CT+A**	Li 2023 (66)	NCT03878472	single-arm	II	China	25	63 (48-70)	76	100	Camrelizumab+Apatinib+Chemo (S-1±oxaliplatin)	None
**ICI+CT+A**	Xu 2023 (67)	/	single-arm	/	China	30	58 (41-74)	73.3	73.3	Sintilimab/Camrelizumab+Apatinib+Chemo (SOX)	Sintilimab/Camrelizumab+Apatinib+Chemo (SOX)
**ICI+CT+A**	Kawazoe 2024 (68)	NCT04745988	single-arm (Abstract)	II	Japan	32	/	/	6.3	Pembrolizumab+Lenvatinib+Chemo (FLOT)	Pembrolizumab+Lenvatinib+Chemo (FLOT)
**ICI+CT+A**	Chen 2022 (69)	NCT05223088	single-arm (Abstract)	II	China	25	57 (39-73)	76	/	Tislelizumab+Apatinib+Chemo (SOX)	Not reported
**ICI+CT+A**	Li 2025 (21)	NCT04208347	RCT	III	China	180	63 (28-75)	83.9	66.1	Camrelizumab+Apatinib+Chemo (SOX)	Camrelizumab+Apatinib+SOX (first 3 cycles) → Camrelizumab+Apatinib (next 3 cycles)
**ICI+CT+A**	Lin 2024 (22), Lin 2025 (23)	NCT04195828	RCT	II	China	51	63 (57-68)	82.4	90.2	Camrelizumab+Apatinib+Chemo (SAP)	Camrelizumab+Apatinib+Chemo (SAP)
**ICI+CRT**	Wei 2023 (70)	ChiCTR1900024428	single-arm	II	China	34	65.5 (58-68)	82.4	70.6	Sintilimab+Radiotherapy+Chemo (SAP)	Sintilimab+Chemo (SAP)

**ICI+CRT**	Tang 2022 (71)	NCT03631615	single-arm	II	China	36	65.5 (35-72)	77.8	83.3	Camrelizumab+Radiotherapy+Chemo (CapeOX)	Chemo (CapeOX)
**ICI+CRT**	Zhu 2022 (72)	NCT02730546	single-arm	II	United States	31	62 (44-76)	96.8	0	Pembrolizumab+Radiotherapy+Chemo (carboplatin+paclitaxel)	Pembrolizumab
**ICI+CRT**	Wei 2026 (24)	NCT05687357	RCT (Abstract)	IIb	China	35	63 (43-75)	65.7	/	Tislelizumab+Radiotherapy+Chemo (SOX/SAP)	Tislelizumab+Chemo
**ICI+CRT**	Ge 2025 (73)	NCT05918419	single-arm (Abstract)	II	China	24	/	/	/	Serplulimab+Radiotherapy+Chemo (SOX)	Not reported
**ICI+CRT**	Li 2025 (74)	NCT05941481	single-arm (Abstract)	II	China	21	68 (48-76)	85.7	61.9	Tislelizumab+Radiotherapy+Chemo (CapeOX)	Not reported
**ICI+CRT**	Liu 2025 (75)	ChiCTR2100046154	single-arm (Abstract)	II	China	26	/	/	/	Sintilimab+Radiotherapy+Chemo (SOX)	Not reported
**ICI+CRT**	Du 2024 (76)	NCT04061928	single-arm (Abstract)	II	China	43	/	/	/	Toripalimab+Radiotherapy+Chemo (CapeOX)	Not reported
**ICI+CRT**	Blum Murphy	NCT03776487	single-arm	I/II	United States	30	/	/	/	Nivolumab+Ipilimumab+Radiotherapy+Chemo(5-Flu	Nivolumab

	2026 (77)		(Abstract)							orouracil/Oxaliplatin)	
**ICI+CT+H**	Wang 2026 (78)	ChiCTR2300075446[*]	single-arm	II	China	32	65 (34-78)	93.8	56.3	Camrelizumab+Disitamab vedotin+Chemo (S-1)	At the investigator's discretion (regimen not prespecified)
**ICI+CT+H**	Chen 2026 (79)	ChiCTR2400081677[*]	single-arm (Abstract)	II	China	25	58 (37-74)	/	/	Toripalimab+Disitamab vedotin+Chemo (XELOX)	Not reported
**ICI+CT+H**	Liu 2026 (80)	NCT06227325[*]	single-arm (Abstract)	II	China	28	/	/	35.7	Sintilimab+Disitamab vedotin+Chemo (XELOX)	Sintilimab+Disitamab vedotin+Chemo (XELOX)
**ICI+CT+H**	Stein 2025 (81)	NCT05504720[#]	single-arm	II	Germany	31	65 (33-76)	80.6	3.2	Pembrolizumab+Trastuzumab+Chemo (FLOT)	Pembrolizumab+Trastuzumab+Chemo (FLOT)
**ICI+CT+H**	Ma 2025 (82)	NCT03950271[#]	single-arm	II	China	25	61 (50-75)	80	56	Camrelizumab+Trastuzumab+Chemo (CapeOX)	Chemo (CapeOX)
**ICI+CT+H**	Nie 2025 (83)	ChiCTR2200058732[#]	single-arm	II	China	22	61 (52-69)	72.7	72.7	Sintilimab+Trastuzumab+Chemo (SOX)	Sintilimab+Chemo (SOX) (± Trastuzumab if HER2+)
**ICI+CT+H**	Wang 2024 (84)	NCT04819971[#]	single-arm (Abstract)	II	China	23	/	/	/	Tislelizumab+Trastuzumab+Chemo (DOS)	Tislelizumab+Trastuzumab+Chemo (SOX)
**ICI+CT+H**	Peng 2025 (25)	NCT04661150[#]	RCT	II	China	21	61 (33-72)	90.5	/	Atezolizumab+Trastuzumab+Chemo (XELOX)	Atezolizumab+Trastuzumab+Chemo (XELOX)
**ICI+ICI**	Raimondi	NCT04817826	single-	II	Italy	18	71.5	66.7	38.9	Tremelimumab+Durvalum	None

	2025 (85)		arm				(65-80)			ab	
**ICI+ICI**	Andre 2023 (86)	NCT04006262	single-arm	II	France	32	65 (40-80)	71.9	0	Nivolumab+Ipilimumab	None

Abbreviations: FLOT: Fluorouracil, Leucovorin, Oxaliplatin and Docetaxel; **SOX:** S-1 and Oxaliplatin; **SAP:** S-1 and Nab-paclitaxel; **DOS:** Docetaxel, Oxaliplatin and S-1; **DCF:** Docetaxel, Cisplatin and Fluorouracil; **CapeOX (XELOX):** Capecitabine and Oxaliplatin; **mFOLFOX6:** Leucovorin, Fluorouracil and Oxaliplatin; **DOC:** Docetaxel, Oxaliplatin and Capecitabine; **FP:** Fluorouracil and Cisplatin; **XP:** Capecitabine and Cisplatin; **FOLFIRINOX:** Leucovorin, Fluorouracil, Irinotecan and Oxaliplatin.

[*]Wang 2026, Chen 2026, Liu 2026 included patients with IHC 2+ or 3+, [#] Stein 2025, Ma 2025, Nie 2025, Wang 2024, Peng 2025 included patients with IHC 3+ or IHC 2+ with ISH+.

eTable 8. Methodological Quality of Single-Arm Studies Assessed with MINORS Checklist

Study	Q1	Q2	Q3	Q4	Q5	Q6	Q7	Q8	Quality score
**Liu 2026 (28)**	2	2	2	2	1	1	2	2	14
**Zhao 2026 (29)**	2	2	2	2	1	2	2	0	13
**Santos 2026 (30)**	2	2	2	2	1	2	1	2	14
**Zhan 2026 (31)**	2	2	2	2	1	2	2	2	15
**Lin 2026 (32)**	2	2	2	2	1	2	2	2	15
**Zhao 2025 (33)**	2	2	2	2	1	2	2	1	14
**Alsina 2025 (34)**	2	2	2	2	1	2	2	2	15
**Alcindor 2025 (35)**	2	2	2	2	1	2	2	2	15
**Long 2025 (36)**	2	2	2	2	1	2	2	2	15
**Kang 2025 (37)**	2	2	2	2	1	2	2	2	15
**He 2025 (38)**	2	2	2	2	1	2	2	2	15
**Nie 2025 (39)**	2	2	2	2	1	2	2	2	15
**Wang 2025 (40)**	2	2	2	2	1	2	2	2	15
**Zhao 2024 (41)**	2	2	2	2	1	2	2	2	15
**Li 2024 (42)**	2	2	2	2	1	2	2	1	14
**Zhong 2024 (43)**	2	2	2	2	1	2	2	2	15
**Verschoor 2024 (44)**	2	2	2	2	1	2	2	1	14
**Lin 2024 (45)**	2	2	2	2	1	1	2	1	13
**Manji 2023 (46)**	2	2	2	2	1	2	2	2	15
**Tang 2023 (47)**	2	2	2	2	1	2	2	1	14
**Guo 2022 (48)**	2	2	2	2	1	2	2	2	15
**Jiang 2022 (49)**	2	2	2	2	1	2	2	2	15

**Zhang 2026 (50)**	2	2	2	2	1	2	2	0	13
**Qiu 2026 (51)**	2	2	2	2	1	1	1	0	11
**Zhao 2026 (52)**	2	2	2	2	1	1	2	0	12
**Xu 2026 (53)**	2	2	2	2	1	1	2	0	12
**Liang 2026 (54)**	2	2	2	2	1	1	2	0	12
**Hu 2025 (55)**	2	2	2	2	1	1	2	1	13
**Wei 2025 (56)**	2	2	2	2	1	1	2	0	12
**Cheng 2025 (57)**	2	2	2	2	1	2	2	1	14
**Nie 2025 (58)**	2	2	2	2	1	2	2	1	14
**He 2025 (59)**	2	2	2	2	1	1	2	0	12
**Li 2024 (60)**	2	2	2	2	1	2	2	2	15
**Zhou 2024 (61)**	2	2	2	2	1	1	2	0	12
**Jiang 2022 (62)**	2	2	2	2	1	2	2	0	13
**Ding 2022 (63)**	2	2	2	2	1	0	2	0	11
**Zhou 2024 (64)**	2	2	2	2	1	2	2	0	13
**Liu 2024 (65)**	2	2	2	2	1	2	2	0	13
**Li 2023 (66)**	2	2	2	2	1	2	2	0	13
**Xu 2023 (67)**	2	2	2	2	1	2	2	0	13
**Kawazoe 2024 (68)**	2	2	2	2	1	1	2	2	14
**Chen 2022 (69)**	2	2	2	2	1	0	1	0	10
**Wei 2023 (70)**	2	2	2	2	1	2	2	2	15
**Tang 2022 (71)**	2	2	2	2	2	2	2	2	16
**Zhu 2022 (72)**	2	2	2	2	1	2	2	2	15
**Ge 2025 (73)**	2	2	2	2	1	1	2	0	12
**Li 2025 (74)**	2	2	2	2	1	1	2	0	12

**Liu 2025 (75)**	2	2	2	2	1	2	2	0	13
**Du 2024 (76)**	2	2	2	2	1	2	2	0	13
**Blum Murphy 2026 (77)**	2	2	2	2	1	2	1	2	14
**Wang 2026 (78)**	2	2	2	2	1	1	1	0	11
**Chen 2026 (79)**	2	2	2	2	1	1	2	0	12
**Liu 2026 (80)**	2	2	2	2	1	1	2	0	12
**Stein 2025 (81)**	2	2	2	2	1	1	2	2	14
**Ma 2025 (82)**	2	2	2	2	1	2	2	2	15
**Nie 2025 (83)**	2	2	2	2	1	2	2	2	15
**Wang 2024 (84)**	2	2	2	2	1	1	2	0	12
**Raimondi 2025 (85)**	2	2	2	2	1	2	2	2	15
**Andre 2023 (86)**	2	2	2	2	1	2	2	2	15

Numbers Q1-Q8 in heading signified: Q1, A clearly stated aim: The question addressed should be precise and relevant in the light of available literature; **Q2, Inclusion of consecutive patients:** All patients potentially fit for inclusion (satisfying the criteria for inclusion) have been included in the study during the study period (no exclusion or details about the reasons for exclusion); **Q3, Prospective collection of data:** Data were collected according to a protocol established before the beginning of the study; **Q4, Endpoints appropriate to the aim of the study:** Unambiguous explanation of the criteria used to evaluate the main outcome which should be in accordance with the question addressed by the study. Also, the endpoints should be assessed on an intention-to-treat basis; **Q5, Unbiased assessment of the study endpoint:** Blind evaluation of objective endpoints and double-blind evaluation of subjective endpoints. Otherwise the reasons for not blinding should be stated; **Q6, Follow-up period appropriate to the aim of the study:** The follow-up should be sufficiently long to allow the assessment of the main endpoint and possible adverse events; **Q7, Loss to follow up less than 5%:** All patients should be included in the follow up. Otherwise, the proportion lost to follow up should not exceed the proportion experiencing the major endpoint; **Q8, Prospective calculation of the study size:** Information of the size of detectable difference of interest with a calculation of 95% confidence interval, according to the expected incidence of the outcome event, and information about the level for statistical significance and estimates of power when comparing the outcomes.

eTable 9. Study-Level Characteristics of TRAEs and irAEs of RCTs Included in the Pairwise Meta-analysis

Group	Study	TRAEs, n/N	irAEs, n/N	Reported safety observation window	Analysis denominator	Grading system/ version	Attribution criteria
ICI+CT vs. chemotherapy	Xu 2026 (7)	**Tislelizumab+CT arm**<br>**Neoadjuvant:** NR.<br>**Perioperative:** any grade, NR; Grade ≥3, 17/68.<br>**Control arm**<br>**Neoadjuvant:** NR.<br>**Perioperative:** any grade, NR; Grade ≥3, 15/68.	**Tislelizumab+CT arm**<br>**Neoadjuvant:** NR.<br>**Perioperative:** any grade, 10/68; Grade ≥3, 4/68.<br>**Control arm**<br>**Neoadjuvant:** NR.<br>**Perioperative:** NR.	Perioperative: TRAEs and irAEs were reported as occurring "during treatment"; exact observation boundaries NR.	The safety population was defined as patients who received at least one dose of trial drug.	NR	TRAEs were reported as treatment-related and irAEs as immune-related; specific causality/attribution criteria NR.
ICI+CT vs. chemotherapy	Shen 2026 (8)	**Serplulimab arm**<br>**Neoadjuvant:** any grade, 212/292; Grade ≥3, 84/292.<br>**Perioperative:** any grade, 284/292; Grade ≥3, 136/292.<br>**Control arm**<br>**Neoadjuvant:** any grade, 198/294; Grade ≥3, 83/294.<br>**Perioperative:** any grade, 288/294; Grade ≥3, 172/294.	**Serplulimab arm**<br>**Neoadjuvant:** any grade, 65/292; Grade ≥3, 6/292.<br>**Perioperative:** any grade, 117/292; Grade ≥3, 18/292.<br>**Control arm**<br>**Neoadjuvant:** any grade, 10/294; Grade ≥3, 1/294.<br>**Perioperative:** any grade, 19/294; Grade	Neoadjuvant: TRAEs and irAEs were reported as occurring "during the neoadjuvant phase"; exact observation boundaries NR.<br>Perioperative: Aggregate safety results were reported for the safety set, and the original report stated that "Safety was monitored throughout the trial," through the August	Safety and immunogenicity were evaluated in patients who received at least one dose of assigned treatment.	NCI-CTCAE v5.0	TRAEs were related to any study treatment. irAEs were study-categorized; detailed causality threshold and assessor NR.

			≥3, 3/294.	19, 2025 data cutoff. The adjuvant phase began 3-12 weeks after surgery. The duration of AE follow-up after the last dose was NR.			
ICI+CT vs. chemotherapy	Janjigian 2025 (10)	**Durvalumab arm** **Neoadjuvant:** NR. **Perioperative:** any grade, 453/475; Grade 3-4, 283/475. **Control arm** **Neoadjuvant:** NR. **Perioperative:** any grade, 444/469; Grade 3-4, 277/469.	**Durvalumab arm** **Neoadjuvant:** NR. **Perioperative:** any grade, 110/475; Grade 3-4, 34/475. **Control arm** **Neoadjuvant:** NR. **Perioperative:** any grade, 34/469; Grade 3-4, 17/469.	Perioperative: TRAEs and immune-mediated AEs were reported as occurring "during the overall treatment period," from the first dose through 90 days after the last dose or initiation of subsequent anticancer therapy, whichever occurred first.	The safety analysis population comprised all participants who received at least one dose of trial treatment; a control-assigned participant who received durvalumab was analyzed with the durvalumab safety population.	NCI-CTCAE v5.0	Investigators assessed possible relationships to durvalumab/placebo or FLOT. Immune-mediated AEs excluded infusion and hypersensitivity reactions.
ICI+CT vs. chemotherapy	Shitara 2024 (11)	**Pembrolizumab arm** **Neoadjuvant:** NR. **Perioperative:** any grade, 379/399; Grade ≥3, 259/399. **Control arm** **Neoadjuvant:** NR. **Perioperative:** any grade, 385/400; Grade ≥3,	NR	Perioperative: TRAEs and prespecified immune-related safety events were reported for the overall study period. AEs were collected "throughout the study" and through 30 days after treatment discontinuation;	Safety was assessed in the per-protocol population, defined as randomized patients who received at least one dose of study treatment.	NCI-CTCAE v4.0	Investigators attributed TRAEs to study treatment. Prespecified AESIs and infusion reactions were collected regardless of attribution and were not treated as irAEs.

		252/400.		serious AEs through 90 days after treatment discontinuation.			
ICI+CT vs. chemotherapy	Shitara 2024 (11)	**Pembrolizumab+FLOT arm** **Neoadjuvant:** NR. **Perioperative:** any grade, 98/99; Grade ≥3, 75/99. **Placebo+FLOT arm** **Neoadjuvant:** NR. **Perioperative:** any grade, 101/103; Grade ≥3, 65/103.	NR	Perioperative: Drug-related AEs were reported as occurring in "all phases combined." AEs were collected throughout the study and through 30 days after treatment discontinuation; serious AEs through 90 days after treatment discontinuation.	Safety was assessed in the per-protocol population, defined as randomized patients who received at least one dose of study treatment.	NCI-CTCAE v4.0	Investigators attributed TRAEs to study treatment. Prespecified AESIs and infusion reactions were collected regardless of attribution and were not treated as irAEs.
ICI+CT vs. chemotherapy	Lorenzen 2024 (14)	NR	NR	NA	NA	NA	NA
ICI+CT vs. chemotherapy	Yuan 2024 (15)	**Toripalimab arm** **Neoadjuvant:** any grade, 48/54; Grade 3-4, 13/54. **Perioperative:** any grade, 52/54; Grade 3-4, 19/54. **Chemotherapy arm** **Neoadjuvant:** any grade, 43/54; Grade 3-4, 11/54. **Perioperative:** any grade, 52/54; Grade 3-4, 16/54.	**Toripalimab arm** **Neoadjuvant:** any grade, 4/54; Grade 3-4, 1/54. **Perioperative:** any grade, 7/54; Grade 3-4, 1/54. **Chemotherapy arm** **Neoadjuvant:** any grade, 0/54; Grade	Neoadjuvant: TRAEs and irAEs were reported as occurring "during preoperative treatment"; exact observation boundaries NR. Perioperative: Aggregate TRAEs and irAEs were reported in the overall "Summary of adverse	All randomized patients underwent perioperative therapy, and nonsurgical adverse events and serious adverse events were analyzed in the intention-to-treat population.	NCI-CTCAE v5.0	The study directly categorized events as TRAEs and irAEs; detailed causality threshold and assessor NR.

			3-4, 0/54.<br>**Perioperative:** any grade, 1/54; Grade 3-4, 0/54.	events"; exact observation boundaries NR.			
ICI+CT vs. chemotherapy	Li 2026 (17)	NR	NR	NA	NA	NA	NA
ICI+CT vs. chemotherapy	Yang 2025 (18)	NR	NR	NA	NA	NA	NA
ICI+CT vs. chemotherapy	Ding 2025 (19)	**Sintilimab + SOX arm**<br>**Phase unspecified:** any grade NR; Grade 3-5, 19/131.<br>**SOX arm**<br>**Phase unspecified:** any grade NR; Grade 3-5, 11/133.	NR	Phase unspecified: TRAEs were reported without a treatment-phase designation; exact observation boundaries NR.	The conference abstract reported outcome-specific denominators but did not define a safety analysis population.	NR	Reported as TRAEs; operational causality criteria and responsible assessor NR.
ICI+CT vs. chemotherapy	Min 2022 (20)	NR	NR	NA	NA	NA	NA
ICI+CT+A vs. chemotherapy	Li 2025 (21)	**SOXRC arm**<br>**Neoadjuvant:** any grade, 157/179; Grade ≥3, 60/179.<br>**Perioperative:** any grade, 168/179; Grade ≥3, 97/179.<br>**SOX arm**<br>**Neoadjuvant:** any grade,	**SOXRC arm**<br>**Neoadjuvant:** any grade, 40/179; Grade ≥3, 11/179.<br>**Perioperative:** any grade, 65/179; Grade ≥3, 23/179.<br>**SOX arm**	Neoadjuvant: TRAEs were reported as occurring "in the neoadjuvant phase," and irAEs as occurring "during neoadjuvant therapy"; exact observation boundaries	Safety analysis included patients who received study treatment among the prespecified randomized cohort.	NCI-CTCAE v5.0	Reported as TRAEs/irAEs; irAEs were stated to occur only in the SOXRC arm. Operational causality criteria and responsible assessor NR.

		142/178; Grade ≥3, 30/178.<br>**Perioperative:** any grade, 159/178; Grade ≥3, 52/178.	**Neoadjuvant:** any grade, 0/178; Grade ≥3, 0/178.<br>**Perioperative:** NR.	NR.<br>Perioperative: Aggregate TRAEs and irAEs were reported as occurring "throughout the treatment period." AEs were monitored through 30 days after the last dose of study treatment; exact starting boundary NR.			
ICI+CT+A vs. chemotherapy	Lin 2024 (22), Lin 2025 (23)	**CA-SAP arm**<br>**Neoadjuvant:** any grade, 47/51; Grade 3-4, 17/51.<br>**Perioperative:** any grade, NR; Grade 3-4, 22/51.<br>**SAP arm**<br>**Neoadjuvant:** any grade, 52/53; Grade 3-4, 14/53.<br>**Perioperative:** any grade, NR; Grade 3-4, 17/53.	**CA-SAP arm**<br>**Neoadjuvant:** any grade, 10/51; Grade 3-4, 0/51.<br>**Perioperative:** any grade, 17/51; Grade 3-4, 1/51.<br>**SAP arm**<br>**Neoadjuvant:** any grade, 1/53; Grade 3-4, 0/53.<br>**Perioperative:** NR.	Neoadjuvant: TRAEs and irAEs were reported as occurring "during the neoadjuvant treatment periods"; exact observation boundaries NR.<br>Perioperative: TRAEs and irAEs were reported as occurring "during perioperative treatment/therapy." AE evaluation continued through 90 days after the last administration of camrelizumab and 30	Safety analyses were performed in all patients who received at least one dose of allocated treatment; this was also the modified intention-to-treat population.	NCI-CTCAE v5.0	Reported as TRAEs/irAEs; perioperative irAEs were reported only for the CA-SAP arm. Operational causality criteria and responsible assessor NR.

				days after the last administration of other trial medications; exact starting boundary NR.			
ICI+CRT vs. chemotherapy	Wei 2026 (24)	**ICI+CRT arm**<br>**Phase unspecified:** any grade, NR; Grade ≥3, 5/35.<br>**Chemo arm**<br>**Phase unspecified:** any grade, NR; Grade ≥3, 2/19.	**NR**	Phase unspecified: TRAEs and irAEs were reported without a treatment-phase designation; exact observation boundaries NR. Whether postoperative events were included and the exact AE observation boundaries were NR.	The conference abstract reported outcome-specific denominators but did not define a safety analysis population.	NR	Reported as TRAEs/irAEs; operational causality criteria and responsible assessor NR.

eTable 10. Study-Level Characteristics of TRAEs and irAEs in Studies Included in the Single-Arm Meta-analysis

Group	Study	TRAEs, n/N	irAEs, n/N	Reported safety observation window	Analysis denominator	Grading system/ version	Attribution criteria
ICI+CT	Liu 2026 (28)	Neoadjuvant: NR<br>Perioperative: any grade, 24/24; Grade 3, 1/24	Neoadjuvant: NR<br>Perioperative: any grade, 4/24; Grade ≥3, 0/24	**Perioperative:** TRAEs and irAEs were reported as occurring "during perioperative therapy"; exact observation boundaries NR.	All enrolled patients received at least one dose of study treatment.	NCI-CTCAE v5.0	Reported as TRAEs/irAEs; operational causality criteria and responsible assessor NR
ICI+CT	Zhao 2026 (29)	Neoadjuvant: NR<br>Perioperative: any grade, 16/17; Grade 3-4, 6/17	NR	**Perioperative:** TRAEs were reported as occurring "during therapy and up to 3 months post-treatment."	All enrolled patients completed the planned neoadjuvant study treatment.	NCI-CTCAE v4.03	Reported as TRAEs; operational causality criteria and responsible assessor NR
ICI+CT	Santos 2026 (30)	Neoadjuvant: any grade, 67/67; Grade ≥3, NR<br>Perioperative: NR	Neoadjuvant: NR<br>Perioperative: any grade, NR; Grade 3, 5/67	**Neoadjuvant:** TRAEs and irAEs were reported as occurring "in the pre-operative period"; exact observation boundaries NR.	The safety population comprised all patients who started study treatment.	NCI-CTCAE v5.0	TRAEs were attributed to FLOT and/or spartalizumab; Grade 3 events were reported as immune-mediated; operational causality criteria and responsible assessor NR

ICI+CT	Zhan 2026 (31)	Neoadjuvant: any grade, 15/33; Grade ≥3, 0/33<br>Perioperative: NR	NR	**Neoadjuvant:** TRAEs were reported as occurring “during neoadjuvant therapy”; exact observation boundaries NR.	All patients received ≥1 dose of study treatment.	NCI-CTCAE v5.0	Reported as TRAEs; operational causality criteria and responsible assessor NR
ICI+CT	Lin 2026 (32)	Neoadjuvant: any grade, 32/49; Grade 3-4, 6/49<br>Perioperative: NR	NR	**Neoadjuvant:** TRAEs were assessed from initiation of neoadjuvant therapy through 28 days after the last dose, or until resolution if ongoing.	All enrolled patients completed neoadjuvant treatment.	NCI-CTCAE v5.0	Reported as TRAEs; irAEs were identified based on clinical presentation and laboratory findings; responsible assessor NR
ICI+CT	Zhao 2025 (33)	Neoadjuvant: NR<br>Perioperative: any grade, 12/16; Grade 3-4, 6/16	Neoadjuvant: NR<br>Perioperative: any grade, 4/16; Grade 3-4, 1/16	**Perioperative:** TRAEs and irAEs were reported as observed “throughout the neoadjuvant therapy and subsequent postoperative adjuvant therapy stages”; exact observation boundaries NR.	The treated efficacy and safety population included all patients who received at least one study treatment.	NCI-CTCAE v4.03	Reported as TRAEs/irAEs; operational causality criteria and responsible assessor NR
ICI+CT	Alsina 2025 (34)	Neoadjuvant: NR.<br>Perioperative: any grade, NR; Grade ≥3, 32/40.	NR	**Perioperative:** TRAEs were reported in the aggregate “Safety” results. The study defined the “overall treatment duration” as including preoperative therapy, surgery, and postoperative therapy; exact observation boundaries NR.	All enrolled patients received study treatment.	NCI-CTCAE v4.0	The abstract labels the Grade ≥3 aggregate as treatment-related, whereas the main-text Safety section labels it as Grade ≥3 AEs.

							Grade ≥3 avelumab-related AEs, 10/40, were a subset. AESIs were not treated as aggregate irAEs.
ICI+CT	Alcindor 2025 (35)	Neoadjuvant: NR<br>Perioperative: any grade, NR; Grade 3-4, 20/50	NR	**Perioperative:** TRAEs were reported as observed “safety during neoadjuvant and adjuvant treatment”; exact observation boundaries NR.	Safety was evaluated in the safety-evaluable population, N=51. Grade 3-4 TRAEs were reported for N=50; reason for the different denominator NR.	NCI-CTCAE v3.0	Grade 3-4 events were reported as treatment-related; operational causality criteria and responsible assessor NR
ICI+CT	Long 2025 (36)	Neoadjuvant: any grade, 38/38; Grade 3, 12/38<br>Perioperative: NR	NR	**Neoadjuvant:** TRAEs were reported as occurring “during neoadjuvant treatment.” Safety was defined as TRAEs occurring during the NAT period and through 30 days after the last dose or before initiation of new antitumor therapy.	Patients who received at least one neoadjuvant treatment were included in the safety assessment.	NCI-CTCAE v5.0	Reported as TRAEs; operational causality criteria and responsible assessor NR
ICI+CT	Kang 2025 (37)	Neoadjuvant: any grade, 50/50; Grade ≥3, 21/50<br>Perioperative: NR	NR	**Neoadjuvant:** TRAEs were reported as occurring during neoadjuvant chemotherapy; exact observation boundaries NR.	The main-arm safety population included patients who received at least one	NCI-CTCAE v5.0	Reported as TRAEs; operational causality criteria and responsible assessor

					dose of neoadjuvant treatment.		NR
ICI+CT	He 2025 (38)	Neoadjuvant: NR<br>Perioperative: any grade, 42/46; Grade 3, 9/46	Neoadjuvant: NR<br>Perioperative: any grade, 31/46; Grade ≥3, 0/46	**Perioperative:** TRAEs and irAEs were reported as occurring "during perioperative therapy"; exact observation boundaries NR.	All enrolled patients received at least one dose of study treatment.	NCI-CTCAE v5.0	Reported as TRAEs/irAEs; operational causality criteria and responsible assessor NR
ICI+CT	Nie 2025 (39)	Neoadjuvant: NR<br>Perioperative: any grade, 32/32; Grade 3-4, 10/32	Neoadjuvant: NR<br>Perioperative: any grade, 8/32; Grade 3, 1/32	**Perioperative:** TRAEs and irAEs were reported as occurring "during perioperative therapy"; exact observation boundaries NR.	All included patients were used to analyze nonsurgical adverse events and serious adverse events; all received at least one cycle of assigned neoadjuvant treatment.	NCI-CTCAE v5.0	Reported as TRAEs/irAEs; operational causality criteria and responsible assessor NR
ICI+CT	Wang 2025 (40)	Neoadjuvant: any grade, 34/37; Grade 3, 9/37<br>Perioperative: NR	Neoadjuvant: any grade, 5/37; Grade ≥3, 0/37<br>Perioperative: NR	**Neoadjuvant:** TRAEs and irAEs were reported during neoadjuvant immunochemotherapy; exact observation boundaries NR.	Enrolled patients received neoadjuvant immunochemotherapy	NCI-CTCAE v5.0	Reported as TRAEs and potential irAEs; operational causality criteria and responsible assessor NR
ICI+CT	Zhao 2024 (41)	NR	NR	NA	NA	NA	NA
ICI+CT	Li 2024 (42)	Neoadjuvant: any grade, 32/32; Grade	NR	**Neoadjuvant:** TRAEs were reported as occurring "during neoadjuvant	The safety analysis set comprised	NCI-CTCAE v5.0	Reported as TRAEs; operational causality

		≥3, 19/32<br>Perioperative: NR		therapy"; exact observation boundaries NR.	patients who received at least one dose of study treatment and had available safety records.		criteria and responsible assessor NR
ICI+CT	Zhong 2024 (43)	NR	Neoadjuvant: NR<br>Perioperative: any grade, 13/29; Grade ≥3, 0/29	**Perioperative:** irAEs were reported in the aggregate safety results. Safety profiles were assessed from initiation of neoadjuvant therapy through 30 days after the last adjuvant treatment.	All enrolled patients completed neoadjuvant treatment.	NCI-CTCAE v5.0	Reported as irAEs; operational causality criteria and responsible assessor NR; TEAEs were not treated as TRAEs
ICI+CT	Verschoor 2024 (44)	NR	Neoadjuvant: any grade, 11/20; Grade ≥3, 2/20<br>Perioperative: NR	**Neoadjuvant:** irAEs were reported during neoadjuvant treatment. Patients were monitored for AEs until 100 days after the last study-treatment dose.	Safety analyses were performed in the per-protocol population, defined as all patients who received at least one dose of the study drugs.	NCI-CTCAE v4.03	Included AEs considered at least possibly related to atezolizumab
ICI+CT	Lin 2024 (45)	NR	NR	NA	NA	NA	NA
ICI+CT	Manji 2023 (46)	Neoadjuvant: NR<br>Perioperative: any grade, NR; Grade ≥3, 20/35	Neoadjuvant: NR<br>Perioperative: any grade, NR; Grade ≥3, 12/35	**Perioperative:** TRAEs and irAEs were reported for perioperative treatment followed by pembrolizumab maintenance; exact observation boundaries NR.	The safety population included all patients who received at least one dose of study	NCI-CTCAE v4.0	Reported as treatment-related and immune-related events; operational causality criteria and

					treatment.		responsible assessor NR
ICI+CT	Tang 2023 (47)	Neoadjuvant: NR<br>Perioperative: any grade, 27/30; Grade 3-4, 11/30	Neoadjuvant: NR<br>Perioperative: any grade, 11/30; Grade 3-4, 1/30	**Perioperative:** TRAEs and irAEs were reported as occurring "during perioperative treatment"; exact observation boundaries NR.	The intention-to-treat population included all patients who received at least one dose of any study drug.	NCI-CTCAE v4.03	TRAEs were reported by the study; events attributed to LP002 or LP002 plus chemotherapy were categorized as irAEs
ICI+CT	Guo 2022 (48)	Neoadjuvant: any grade, 27/30; Grade ≥3, NR<br>Perioperative: NR	NR	**Neoadjuvant:** TRAEs were reported as occurring "during the neoadjuvant therapy period"; exact observation boundaries NR.	All enrolled patients completed neoadjuvant study treatment.	NCI-CTCAE v5.0	Reported as TRAEs; operational causality criteria and responsible assessor NR
ICI+CT	Jiang 2022 (49)	Neoadjuvant: any grade, 33/36; Grade 3, 10/36<br>Perioperative: NR	Neoadjuvant: any grade, 3/36; Grade ≥3, 0/36<br>Perioperative: NR	**Neoadjuvant:** TRAEs and irAEs were reported as occurring "during the neoadjuvant treatment period"; exact observation boundaries NR.	The safety population included patients administered at least one dose of neoadjuvant or adjuvant study treatment.	NCI-CTCAE v5.0	TRAEs were treatment-related to any study drug; immune-related events were described as AEs with potential immunologic etiology; responsible assessor NR
ICI+CT	Zhang 2026 (50)	Treatment phase unspecified: any	NR	**Phase unspecified:** TRAEs were reported; treatment phase and exact	Reported-result denominator: N=37;	NR	Reported as TRAEs; operational causality

		grade, NR; Grade 3-4, 9/37 Phase-specific values: NR		observation boundaries NR.	formal safety-set definition NR.		criteria and responsible assessor NR
ICI+CT	Qiu 2026 (51)	Treatment phase unspecified: any grade, NR; Grade 3-4, 18/56 Phase-specific values: NR	NR	**Phase unspecified:** TRAEs were reported; treatment phase and exact observation boundaries NR.	Reported-result denominator: N=56; formal safety-set definition NR.	NR	Reported as treatment-related adverse events; operational causality criteria and responsible assessor NR
ICI+CT	Zhao 2026 (52)	Treatment phase unspecified: any grade, 30/30; Grade ≥3, 6/30 Phase-specific values: NR	NR	**Phase unspecified:** TRAEs were reported; treatment phase and exact observation boundaries NR.	All enrolled patients completed neoadjuvant study treatment.	NR	Reported as treatment-related events; operational causality criteria and responsible assessor NR
ICI+CT	Xu 2026 (53)	Treatment phase unspecified: any grade, 38/40; Grade ≥3, 6/40 Phase-specific values: NR	NR	**Phase unspecified:** TRAEs were reported; treatment phase and exact observation boundaries NR.	All enrolled patients received at least one cycle of neoadjuvant treatment.	NR	Reported as TRAEs; operational causality criteria and responsible assessor NR
ICI+CT	Liang 2026 (54)	NR	NR	NA	NA	NA	NA
ICI+CT	Hu 2025 (55)	Treatment phase unspecified: any	NR	**Phase unspecified:** TRAEs were reported; treatment phase and exact	The safety analysis set excluded patients	NR	Reported as treatment-related

		grade, NR; Grade 3-4, 1/27<br>Phase-specific values: NR		observation boundaries NR.	who were still receiving preoperative treatment.		events; operational causality criteria and responsible assessor NR
ICI+CT	Wei 2025 (56)	NR	NR	NA	NA	NA	NA
ICI+CT	Cheng 2025 (57)	NR	NR	NA	NA	NA	NA
ICI+CT	Nie 2025 (58)	Treatment phase unspecified: any grade, NR; Grade 3-4, 11/30<br>Phase-specific values: NR	NR	**Phase unspecified:** TRAEs were reported; treatment phase and exact observation boundaries NR.	All treated patients received at least one assigned neoadjuvant cycle.	NR	Reported as treatment-related events; operational causality criteria and responsible assessor NR
ICI+CT	He 2025 (59)	Neoadjuvant: any grade, 24/27; Grade 3-4, 5/27<br>Perioperative: NR	NR	**Neoadjuvant:** TRAEs were reported as occurring during neoadjuvant therapy; exact observation boundaries NR.	All enrolled patients received neoadjuvant study treatment.	NCI-CTCAE v4.0	Reported as TRAEs; operational causality criteria and responsible assessor NR
ICI+CT	Li 2024 (60)	NR	NR	NA	NA	NA	NA
ICI+CT	Zhou 2024 (61)	NR	NR	NA	NA	NA	NA
ICI+CT	Gao 2024 (26)	NR	NR	NA	NA	NA	NA
ICI+CT	Liu 2023 (27)	NR	NR	NA	NA	NA	NA
ICI+CT	Jiang 2022 (62)	Treatment phase unspecified: any grade, NR; Grade ≥3, 4/35<br>Phase-specific	Treatment phase unspecified: any grade, NR; Grade 3, 1/35<br>Phase-specific	**Phase unspecified:** TRAEs and irAEs were reported; treatment phase and exact observation boundaries NR.	Safety percentages were reported for the enrolled and treated population.	NR	Grade ≥3 TRAE was supported by four Grade 3 neutropenia cases and the statement that no

		values: NR	values: NR				other Grade ≥3 TRAEs occurred; potential irAEs were explicitly labelled, but operational definition and assessor NR
ICI+CT	Ding 2022 (63)	NR	NR	NA	NA	NA	NA
ICI+CT	Xu 2026 (7)	**Neoadjuvant:** NR **Perioperative:** any grade, NR; Grade ≥3, 17/68	**Neoadjuvant:** NR. **Perioperative:** any grade, 10/68; Grade ≥3, 4/68	**Perioperative:** TRAEs and irAEs were reported as occurring "during treatment"; exact observation boundaries NR.	The safety population was defined as patients who received at least one dose of trial drug.	NR	TRAEs were reported as treatment-related and irAEs as immune-related; specific causality/attribution criteria NR.
ICI+CT	Shen 2026 (8)	**Neoadjuvant:** any grade, 212/292; Grade ≥3, 84/292 **Perioperative:** any grade, 284/292; Grade ≥3, 136/292	**Neoadjuvant:** any grade, 65/292; Grade ≥3, 6/292 **Perioperative:** any grade, 117/292; Grade ≥3, 18/292	**Neoadjuvant:** TRAEs and irAEs were reported as occurring "during the neoadjuvant phase"; exact observation boundaries NR. **Perioperative:** Aggregate TRAEs and irAEs were reported for the safety set, with safety monitored "throughout the trial"; exact observation boundaries NR.	The safety set included patients who received at least one dose of assigned treatment.	NCI-CTCAE v5.0	TRAEs were related to any study treatment. irAEs were study-categorized; detailed causality threshold and assessor NR.
ICI+CT	Janjigian 2025	**Neoadjuvant:** NR	Neoadjuvant: NR	**Perioperative:** TRAEs and	The safety analysis	NCI-CTCAE	Investigators

	(10)	**Perioperative:** any grade, 453/475; Grade 3-4, 283/475	Perioperative: any grade, 110/475; Grade 3-4, 34/475	immune-mediated AEs were reported as occurring “during the overall treatment period,” from the first dose through 90 days after the last dose or initiation of subsequent anticancer therapy, whichever occurred first.	population comprised all participants who received at least one dose of trial treatment; a control-assigned participant who received durvalumab was included in this population.	v5.0	assessed possible relationships to durvalumab/placebo or FLOT. Immune-mediated AEs excluded infusion and hypersensitivity reactions.
ICI+CT	Shitara 2024 (11)	**Neoadjuvant:** NR. **Perioperative:** any grade, 379/399; Grade ≥3, 259/399	NR	**Perioperative:** TRAEs and prespecified immune-related safety events were reported for the overall study period. AEs were collected “throughout the study” and through 30 days after treatment discontinuation; serious AEs through 90 days after treatment discontinuation.	Safety was assessed in the per-protocol population, defined as randomized patients who received at least one dose of study treatment.	NCI-CTCAE v4.0	Investigators attributed TRAEs to study treatment. Prespecified AESIs and infusion reactions were collected regardless of attribution and were not treated as irAEs.
ICI+CT	Shitara 2024 (11)	**Neoadjuvant:** NR. **Perioperative:** any grade, 98/99; Grade ≥3, 75/99	NR	**Perioperative:** Drug-related AEs were reported as occurring in “all phases combined.” AEs were collected throughout the study and through 30	Safety was assessed in the per-protocol population, defined as randomized	NCI-CTCAE v4.0	Investigators attributed TRAEs to study treatment. Prespecified AESIs

				days after treatment discontinuation; serious AEs through 90 days after treatment discontinuation.	patients who received at least one dose of study treatment.		and infusion reactions were collected regardless of attribution and were not treated as irAEs.
ICI+CT	Lorenzen 2024 (14)	NR	NR	NA	NA	NA	NA
ICI+CT	Yuan 2024 (15)	**Neoadjuvant:** any grade, 48/54; Grade 3-4, 13/54<br>**Perioperative:** any grade, 52/54; Grade 3-4, 19/54	**Neoadjuvant:** any grade, 4/54; Grade 3-4, 1/54<br>**Perioperative:** any grade, 7/54; Grade 3-4, 1/54	**Neoadjuvant:** TRAEs and irAEs were reported as occurring “during preoperative treatment”; exact observation boundaries NR.<br>**Perioperative:** Aggregate TRAEs and irAEs were reported in the overall “Summary of adverse events”; exact observation boundaries NR.	All randomized patients underwent perioperative therapy, and nonsurgical adverse events and serious adverse events were analyzed in the intention-to-treat population.	NCI-CTCAE v5.0	The study directly categorized events as TRAEs and irAEs; detailed causality threshold and assessor NR.
ICI+CT	Li 2026 (17)	NR	NR	NA	NA	NA	NA
ICI+CT	Yang 2025 (18)	NR	NR	NA	NA	NA	NA
ICI+CT	Ding 2025 (19)	**Phase unspecified:** any grade NR; Grade 3-5, 19/131<br>Phase-specific values: NR	NR	**Phase unspecified:** TRAEs were reported; treatment phase and exact observation boundaries NR.	The conference abstract reported an outcome-specific denominator but did not define a safety	NR	Reported as TRAEs; operational causality criteria and responsible assessor NR.

					analysis population.		
ICI+CT	Min 2022 (20)	NR	NR	NA	NA	NA	NA
ICI+CT+A	Zhou 2024 (64)	Neoadjuvant: any grade, 30/30; Grade 3-4, 10/30<br>Perioperative: NR	NR	**Neoadjuvant:** TRAEs were reported as occurring during neoadjuvant treatment; exact observation boundaries NR.	All patients received neoadjuvant study treatment.	NCI-CTCAE v5.0	Reported as TRAEs; operational causality criteria and responsible assessor NR
ICI+CT+A	Liu 2024 (65)	Neoadjuvant: NR<br>perioperative: any grade, 26/32; Grade ≥3, NR	NR	**Perioperative:** TRAEs were reported as occurring "during the treatment process"; exact observation boundaries NR.	All patients received study treatment.	NCI-CTCAE v5.0	Reported as treatment-related adverse reactions; operational causality criteria and responsible assessor NR
ICI+CT+A	Li 2023 (66)	NR	NR	NA	NA	NA	NA
ICI+CT+A	Xu 2023 (67)	NR	NR	NA	NA	NA	NA
ICI+CT+A	Kawazoe 2024 (68)	NR	NR	NA	NA	NA	NA
ICI+CT+A	Chen 2022 (69)	NR	NR	NA	NA	NA	NA
ICI+CT+A	Li 2025 (21)	**Neoadjuvant:** any grade, 157/179; Grade ≥3, 60/179<br>**Perioperative:** any grade, 168/179; Grade ≥3, 97/179	**Neoadjuvant:** any grade, 40/179; Grade ≥3, 11/179<br>**Perioperative:** any grade, 65/179; Grade ≥3, 23/179	**Neoadjuvant:** TRAEs were reported as occurring "in the neoadjuvant phase," and irAEs as occurring "during neoadjuvant therapy"; exact observation boundaries NR.<br>**Perioperative:** Aggregate TRAEs and	Safety analysis included patients who received study treatment among the prespecified randomized cohort.	NCI-CTCAE v5.0	Reported as TRAEs/irAEs; irAEs were stated to occur only in the SOXRC arm. Operational causality criteria and

				irAEs were reported as occurring "throughout the treatment period." AEs were monitored through 30 days after the last dose of study treatment; exact starting boundary NR.			responsible assessor NR
ICI+CT+A	Lin 2024 (22), Lin 2025 (23)	**Neoadjuvant:** any grade, 47/51; Grade 3-4, 17/51<br>**Perioperative:** any grade, NR; Grade 3-4, 22/51	**Neoadjuvant:** any grade, 10/51; Grade 3-4, 0/51<br>**Perioperative:** any grade, 17/51; Grade 3-4, 1/51	**Neoadjuvant:** TRAEs and irAEs were reported as occurring "during the neoadjuvant treatment periods"; exact observation boundaries NR.<br>**Perioperative:** TRAEs and irAEs were reported as occurring "during perioperative treatment/therapy." AE evaluation continued through 90 days after the last administration of camrelizumab and 30 days after the last administration of other trial medications; exact starting boundary NR.	Safety analyses were performed in all patients who received at least one dose of allocated treatment.	NCI-CTCAE v5.0	Reported as TRAEs/irAEs; perioperative irAEs were reported only for the CA-SAP arm. Operational causality criteria and responsible assessor NR.
ICI+CRT	Wei 2023 (70)	Neoadjuvant: any grade, 31/34; Grade ≥3, NR<br>Perioperative: any grade, 34/34; Grade ≥3, NR	Neoadjuvant: any grade, 11/34; Grade ≥3, NR<br>Perioperative: any grade, 11/34; Grade ≥3, NR	**Neoadjuvant:** TRAEs and irAEs were reported for the "neoadjuvant period"; exact phase-specific boundaries NR.<br>**Perioperative:** Aggregate TRAEs and irAEs were reported for "any periods," from the first dose of the study regimen through 90 days after the last dose.	All enrolled patients received study treatment.	NCI-CTCAE v4.03	TRAEs/irAEs were study-reported; AE judgment used timing, clinical manifestations and relevant tests/examinations, with

							multidisciplinary discussion when necessary; explicit causality threshold and primary assessor NR
ICI+CRT	Tang 2022 (71)	NR	Neoadjuvant: any grade, 31/36; Grade ≥3, NR<br>Perioperative: NR	**Neoadjuvant:** irAEs were reported during neoadjuvant therapy. AEs were assessed from initiation of neoadjuvant therapy through 28 days after the last dose.	The safety population included all patients who received at least one dose of study treatment.	NCI-CTCAE v4.03	Reported as irAEs; operational causality criteria and responsible assessor NR; TEAEs were not treated as TRAEs
ICI+CRT	Zhu 2022 (72)	NR	Neoadjuvant: any grade, NR; Grade ≥3, 3/31<br>Perioperative: NR	**Neoadjuvant:** irAEs were reported as occurring "during neoadjuvant therapy." Neoadjuvant AEs were defined from the start of neoadjuvant therapy through 90 days after the last neoadjuvant dose or until surgery, whichever occurred first.	All evaluable patients started neoadjuvant therapy per protocol.	NCI-CTCAE v4.03	All AEs were prospectively collected with grade and attribution; operational criteria for classifying irAEs and responsible assessor NR
ICI+CRT	Wei 2026 (24)	**Phase unspecified:** any grade, NR; Grade ≥3, 5/35. Phase-specific values: NR	NR	**Phase unspecified:** TRAEs were reported; treatment phase and exact observation boundaries NR.	The conference abstract reported an outcome-specific denominator but did not define a safety analysis population.	NR	Reported as TRAEs/irAEs; operational causality criteria and responsible assessor NR.

ICI+CRT	Ge 2025 (73)	NR	NR	NA	NA	NA	NA
ICI+CRT	Li 2025 (74)	Treatment phase unspecified: any grade, NR; Grade ≥3, 8/21 Phase-specific values: NR	NR	**Phase unspecified:** TRAEs were reported; treatment phase and exact observation boundaries NR.	The conference abstract reported an outcome-specific denominator based on the enrolled population but did not define a separate safety analysis set.	NR	Reported as TRAEs; operational causality criteria and responsible assessor NR
ICI+CRT	Liu 2025 (75)	NR	NR	NA	NA	NA	NA
ICI+CRT	Du 2024 (76)	NR	NR	NA	NA	NA	NA
ICI+CRT	Blum Murphy 2026 (77)	Treatment phase unspecified: any grade, NR; Grade 3-4, 13/30 Phase-specific values: NR	NR	**Phase unspecified:** TRAEs were reported; treatment phase NR. Patients were monitored for a minimum of 100 days after the final study-drug dose and until AEs resolved, returned to baseline, or were deemed irreversible.	The safety-evaluable population included all patients who received at least one cycle of chemotherapy.	NCI-CTCAE v5.0	The aggregate 13/30 Grade ≥3 result was all-cause AEs and was not used as an aggregate TRAE result. Only Grade 4 treatment-related toxicities were explicitly aggregated (3/30).
ICI+CT+H	Wang 2026 (78)	Neoadjuvant: any grade, 24/32; Grade ≥3, 10/32 Perioperative: NR	NR	**Neoadjuvant:** TRAEs were reported as occurring "during the neoadjuvant period"; exact observation boundaries NR.	The safety analysis set comprised patients who received at least one dose of study	NR	Reported as TRAEs; operational causality criteria and responsible assessor NR

					treatment and had available safety data.		
ICI+CT+H	Chen 2026 (79)	Treatment phase unspecified: any grade, NR; Grade ≥3, 10/25<br>Phase-specific values: NR	Neoadjuvant: NR<br>Perioperative: NR	**Phase unspecified:** TRAEs were reported; treatment phase and exact observation boundaries NR.	The conference abstract reported an outcome-specific denominator based on the enrolled population but did not define a separate safety analysis set.	NR	Reported as treatment-related adverse events; operational causality criteria and responsible assessor NR
ICI+CT+H	Liu 2026 (80)	Treatment phase unspecified: any grade, NR; Grade ≥3, 7/28<br>Phase-specific values: NR	Neoadjuvant: NR<br>Perioperative: NR	**Phase unspecified:** TRAEs were reported; treatment phase and exact observation boundaries NR.	The conference abstract reported an outcome-specific denominator based on the enrolled population but did not define a separate safety analysis set.	NR	Reported as TRAEs; operational causality criteria and responsible assessor NR
ICI+CT+H	Stein 2025 (81)	NR	NR	NA	NA	NA	NA
ICI+CT+H	Ma 2025 (82)	NR	NR	NA	NA	NA	NA
ICI+CT+H	Nie 2025 (83)	Neoadjuvant: any grade, 22/22; Grade ≥3, 3/22<br>Perioperative: any grade, 22/22; Grade	Neoadjuvant: any grade, 4/22; Grade ≥3, 0/22<br>Perioperative: any grade, 4/22; Grade	**Neoadjuvant:** TRAEs and irAEs were reported during neoadjuvant therapy; exact observation boundaries NR.<br>**Perioperative:** TRAEs and irAEs were reported as occurring “during	All enrolled patients received study treatment.	NCI-CTCAE v5.0	Reported as TRAEs/irAEs; operational causality criteria and responsible assessor

		≥3, 6/22	≥3, 0/22	perioperative therapy"; exact observation boundaries NR.			NR
ICI+CT+H	Wang 2024 (84)	NR	NR	NA	NA	NA	NA
ICI+CT+H	Peng 2025 (25)	NR	NR	NA	NA	NA	NA
ICI+ICI	Raimondi 2025 (85)	NR	Neoadjuvant: any grade, NR; Grade ≥3, 3/18 Perioperative: NR	**Neoadjuvant:** Grade ≥3 irAEs were reported in the Cohort 1 safety results. Safety was defined as AEs "during the treatment"; exact observation boundaries NR.	The cohort safety population included all enrolled patients who received at least one dose of study treatment.	NCI-CTCAE v5.0	Grade ≥3 events were explicitly categorized as immune-related AEs; operational irAE definition, causality categories, attribution threshold and responsible assessor NR; any-grade AEs were not treatment-attributed
ICI+ICI	André 2023 (86)	Neoadjuvant: any grade, 24/32; Grade 3-4, 6/32 Perioperative: NR	NR	**Neoadjuvant:** TRAEs were reported as occurring "during neoadjuvant treatment." Safety assessments were performed continuously throughout treatment and for a minimum of 100 days after surgery or treatment discontinuation.	All enrolled patients received neoadjuvant study treatment.	NCI-CTCAE v5.0	TRAEs were explicitly defined as AEs with potential immunologic etiology; a separate aggregate irAE result was not reported; more detailed causality categories,

							attribution threshold and responsible assessor NR

eTable 11. Sensitivity analyses of pairwise meta-analyses

Outcome	Comparison	Analysis	Unique studies, k	Participants, N	Effect estimate (95% CI)	I², %
**pCR**	**ICI+CT vs CT**	Primary analysis	10	3570	3.01 (2.19-4.15)	I2=42.3%
		Excluding abstract-derived outcome data	6	2879	2.75 (1.83-4.12)	I2=60.4%
		Excluding studies at high risk of bias	7	3133	2.80 (1.97-3.96)	I2=49.7%
		Excluding biomarker-enriched cohort	9	2982	3.01 (2.06-4.38)	I2=46.1%
**MPR**	**ICI+CT vs CT**	Primary analysis	8	2869	1.59 (1.36-1.85)	I2=4.4%
		Excluding abstract-derived outcome data	5	2491	1.54 (1.29-1.84)	I2=33.3%
		Excluding studies at high risk of bias	6	2542	1.56 (1.31-1.86)	I2=23.9%
		Excluding biomarker-enriched cohort	8	2869	1.59 (1.36-1.85)	I2=4.4%
**EFS**	**ICI+CT vs CT**	Primary analysis	4	2651	0.74 (0.66-0.83)	I2=0%
		Excluding biomarker-enriched cohort	3	2063	0.75 (0.66-0.85)	I2=1.1%
**Surgical resection**	**ICI+CT vs CT**	Primary analysis	9	3460	1.02 (1.00-1.05)	I2=0%
		Excluding abstract-derived outcome data	6	3082	1.02 (0.99-1.05)	I2=0%
		Excluding studies at high risk of bias	7	3133	1.02 (0.99-1.04)	I2=0%
**R0 resection**	**ICI+CT vs CT**	Primary analysis	9	3460	1.04 (1.01-1.07)	I2=12.6%
		Excluding abstract-derived outcome data	6	2879	1.03 (1.00-1.06)	I2=0%
		Excluding studies at high risk of bias	7	3133	1.03 (1.00-1.06)	I2=0%

eTable 12. Sensitivity analyses of single-arm meta-analyses

Outcome	Strategy	Analysis	Unique studies, k	Participants, N	Pooled proportion (95% CI)	I^2, %
**pCR**	**ICI+CT**	Primary analysis	47	3186	21% (19-23%)	I2=41.7%
		Excluding conference-abstract-derived outcome data	28	2348	21% (19-24%)	I2=56.3%
		Excluding biomarker-enriched cohort	44	2831	20% (19-23%)	I2=26.9%
**pCR**	**ICI+CT+A**	Primary analysis	8	405	19% (15-23%)	I2=0%
		Excluding conference-abstract-derived outcome data	6	348	18% (14-23%)	I2=0%
**pCR**	**single-ICI+CRT**	Primary analysis	8	250	29% (23-35%)	I2=8.2%
		Excluding conference-abstract-derived outcome data	3	101	32% (24-42%)	I2=0%
**pCR**	**trastuzumab-based ICI+CT+H**	Primary analysis	5	122	39% (29-50%)	I2=32.5%
		Excluding conference-abstract-derived outcome data	4	99	39% (27-54%)	I2=47.1%
**MPR**	**ICI+CT**	Primary analysis	44	2809	39% (35-44%)	I2=75.5%
		Excluding conference-abstract-derived outcome data	26	2056	42% (36-48%)	I2=80.2%
		Excluding biomarker-enriched cohort	42	2746	39% (35-44%)	I2=74.6%
**MPR**	**ICI+CT+A**	Primary analysis	7	375	38% (29-48%)	I2=67.0%
		Excluding conference-abstract-derived outcome data	5	318	36% (24-50%)	I2=77.1%
**MPR**	**single-ICI+CRT**	Primary analysis	7	219	52% (37-67%)	I2=74.3%
		Excluding conference-abstract-derived outcome data	2	70	63% (27-89%)	I2=88.2%

**MPR**	**trastuzumab-based ICI+CT+H**	Primary analysis	5	122	54% (45-63%)	I2=0%
		Excluding conference-abstract-derived outcome data	4	99	54% (43-65%)	I2=18.6%

eTable 13. Comparison of Pairwise Meta-Analysis Results Between ITT and PP Populations

	pCR		MPR		R0	
	ITT	PP	ITT	PP	ITT	PP
**ICI+CT vs. chemotherapy**	3.01 (2.19-4.15), I^2=42.3%	2.78 (2.05-3.77), I^2=36.7%	1.59 (1.36-1.85), I^2=4.4%	1.52 (1.33-1.74), I^2=0%	1.04 (1.01-1.07), I^2=12.6%	1.01 (0.99-1.02), I^2=18.4%
**ICI+CT+A vs. chemotherapy**	3.43 (1.85-6.37), I^2=0%	3.44 (1.86-6.35), I^2=0%	1.40 (1.12-1.76), I^2=0%	1.40 (1.14-1.73), I^2=0%	1.08 (0.98-1.20), I^2=33.3%	1.07 (1.00-1.15), I^2=40.7%
**ICI+CRT vs. chemotherapy**	10.44 (0.64-169.86)	10.91 (0.69-173.13)	4.07 (1.04-15.95)	4.24 (1.14-15.70)	NA	NA

eTable 14. Comparison of Single-Arm Meta-Analysis Results Between ITT and PP Populations

	pCR		MPR		R0		DCR		ORR	
	ITT	PP	ITT	PP	ITT	PP	ITT	PP	ITT	PP
**ICI+CT**	21% (19-23%), I^2=41.7%	23% (21-25%), I^2=36.4%	39% (35-44%), I^2=75.5%	43% (38-48%), I^2=73.9%	87% (83-89%), I^2=69.0%	95% (94-96%), I^2=0%	84% (67-93%), I^2=86.8%	94% (90-97%), I^2=0	55% (43-67%), I^2=78.7%	67% (60-72%), I^2=35.4%
**ICI+CT+A**	19% (15-23%), I^2=0%	21% (17-26%), I^2=0%	38% (29-48%), I^2=67.0%	44% (35-54%), I^2=64.1%	89% (77-95%), I^2=76.3%	98% (95-99%), I^2=0%	96% (91-98%), I^2=0%	97% (93-99%), I^2=0%	70% (50-84%), I^2=78.6%	71% (51-86%), I^2=80%
**single-ICI+CRT**	29% (23-35%), I^2=8.2%	34% (27-40%), I^2=0%	52% (37-67%), I^2=74.3%	62% (49-73%), I^2=63.9%	83% (73-90%), I^2=59.6%	98% (95-99%), I^2=0%	NA	NA	NA	NA
**disitamab vedotin-based ICI+CT+H**	26% (18-37%), I^2=6.6%	33% (23-45%), I^2=0	44% (27-63%), I^2=65.5%	55% (43-67%), I^2=0%	70% (57-80%), I^2=0%	98% (85-100%), I^2=0%	NA	NA	NA	NA
**trastuzumab-based ICI+CT+H**	39% (29-50%), I^2=32.5%	43% (32-55%), I^2=31.3%	54% (45-63%), I^2=0%	60% (49-69%), I^2=9.2%	89% (77-95%), I^2=43.8%	97% (91-99%), I^2=0%	71% (22-96%), I^2=73.8%	89% (39-99%), I^2=79.2%	56% (15-91%), I^2=87.3%	71% (25-95%), I^2=88%
**ICI+ICI**	52% (38-65%), I^2=0%	60% (45-74%), I^2=0%	64% (50-76%), I^2=0%	75% (55-88%), I^2=24.5%	NA	NA	NA	NA	NA	NA

eTable 15. The overall certainty of evidence

Certainty assessment							№ of patients		Effect		Certainty	Importance
№ of studies	Study design	Risk of bias	Inconsistency	Indirectness	Imprecision	Other considerations	ICI-based neoadjuvant/perioperative strategies	neoadjuvant/perioperative chemotherapy	Relative (95% CI)	Absolute (95% CI)		
**pCR of ICI+CT vs. chemo**												
10	randomised trials	not serious	not serious	not serious	not serious	none	334/1778 (18.8%)	111/1792 (6.2%)	**RR 3.01** (2.19 to 4.15)	**125 more per 1,000** (from 74 more to 195 more)	⨁⨁⨁⨁ High	IMPORTANT
**MPR of ICI+CT vs. chemo**												
8	randomised trials	not serious	not serious	not serious	not serious	none	482/1431 (33.7%)	307/1438 (21.3%)	**RR 1.59** (1.36 to 1.85)	**126 more per 1,000** (from 77 more to 181 more)	⨁⨁⨁⨁ High	IMPORTANT
**OS for ICI+CT vs. chemo**												
3	randomised trials	not serious	not serious	not serious	not serious	none	1030 participants<br>-	1033 participants<br>-	**HR 0.81** (0.70 to 0.93) [OS for ICI+CT vs. chemo]	-<br>-	⨁⨁⨁⨁ High	CRITICAL

Certainty assessment							№ of patients		Effect		Certainty	Importance
№ of studies	Study design	Risk of bias	Inconsistency	Indirectness	Imprecision	Other considerations	ICI-based neoadjuvant/perioperative strategies	neoadjuvant/perioperative chemotherapy	Relative (95% CI)	Absolute (95% CI)		
**EFS for ICI+CT vs. chemo**												
4	randomised trials	not serious	not serious	not serious	not serious	none	1322 participants<br>-	1329 participants<br>-	**HR 0.74** (0.66 to 0.83) [EFS for ICI+CT vs. chemo]	-<br>-	⊕⊕⊕⊕ High	CRITICAL
**Surgical resection of ICI+CT vs. chemo**												
9	randomised trials	not serious	not serious	not serious	serious[a]	none	1476/1723 (85.7%)	1436/1737 (82.7%)	**RR 1.02** (1.00 to 1.05)	**17 more per 1,000** (from 0 fewer to 41 more)	⊕⊕⊕◯ Moderate[a]	IMPORTANT
**R0 resection for ICI+CT vs. chemo**												
9	randomised trials	not serious	not serious	not serious	not serious	none	1397/1723 (81.1%)	1341/1737 (77.2%)	**RR 1.04** (1.01 to 1.07)	**31 more per 1,000** (from 8 more to 54 more)	⊕⊕⊕⊕ High	IMPORTANT
**Grade ≥3 TRAEs during perioperative therapy, ICI+CT vs. chemo**												

Certainty assessment							№ of patients		Effect		Certainty	Importance
№ of studies	Study design	Risk of bias	Inconsistency	Indirectness	Imprecision	Other considerations	ICI-based neoadjuvant/perioperative strategies	neoadjuvant/perioperative chemotherapy	Relative (95% CI)	Absolute (95% CI)		
5	randomised trials	not serious	serious[b]	serious[c]	not serious	none	789/1387 (56.9%)	797/1390 (57.3%)	**RR 1.01** (0.89 to 1.15)	**6 more per 1,000** (from 63 fewer to 86 more)	⊕⊕◯◯ Low[b,c]	IMPORTANT
**Grade ≥3 TRAEs during neoadjuvant therapy, ICI+CT vs. chemo**												
2	randomised trials	not serious	not serious	serious[c]	serious[a]	none	97/346 (28.0%)	94/348 (27.0%)	**RR 1.04** (0.81 to 1.32)	**11 more per 1,000** (from 51 fewer to 86 more)	⊕⊕◯◯ Low[a,c]	IMPORTANT
**Grade ≥3 irAEs during perioperative therapy, ICI+CT vs. chemo**												
3	randomised trials	not serious	not serious	serious[c]	serious[a]	none	53/821 (6.5%)	20/817 (2.4%)	**RR 2.93** (1.19 to 7.23)	**47 more per 1,000** (from 5 more to 153 more)	⊕⊕◯◯ Low[a,c]	IMPORTANT

Grade ≥3 irAEs during neoadjuvant therapy, ICI+CT vs. chemo

Certainty assessment							№ of patients		Effect		Certainty	Importance
№ of studies	Study design	Risk of bias	Inconsistency	Indirectness	Imprecision	Other considerations	ICI-based neoadjuvant/perioperative strategies	neoadjuvant/perioperative chemotherapy	Relative (95% CI)	Absolute (95% CI)		
2	randomised trials	not serious	not serious	serious[c]	very serious[d]	none	7/346 (2.0%)	1/348 (0.3%)	**RR 4.88** (0.84 to 28.30)	**11 more per 1,000** (from 0 fewer to 78 more)	⨁◯◯◯ Very low[c,d]	IMPORTANT

pCR of ICI+CT+A vs. chemo

2	randomised trials	not serious	not serious	not serious	serious[a]	none	41/231 (17.7%)	12/233 (5.2%)	**RR 3.43** (1.85 to 6.37)	**125 more per 1,000** (from 44 more to 277 more)	⨁⨁⨁◯ Moderate[a]	IMPORTANT

MPR of ICI+CT+A vs. chemo

2	randomised trials	not serious	not serious	not serious	serious[a]	none	109/231 (47.2%)	77/233 (33.0%)	**RR 1.40** (1.12 to 1.76)	**132 more per 1,000** (from 40 more to 251 more)	⨁⨁⨁◯ Moderate[a]	IMPORTANT

Surgical resection of ICI+CT+A vs. chemo

Certainty assessment							№ of patients		Effect		Certainty	Importance
№ of studies	Study design	Risk of bias	Inconsistency	Indirectness	Imprecision	Other considerations	ICI-based neoadjuvant/perioperative strategies	neoadjuvant/perioperative chemotherapy	Relative (95% CI)	Absolute (95% CI)		
2	randomised trials	not serious	not serious	not serious	serious[a]	none	204/231 (88.3%)	206/233 (88.4%)	**RR 1.01** (0.95 to 1.07)	**9 more per 1,000** (from 44 fewer to 62 more)	⊕⊕⊕◯ Moderate[a]	IMPORTANT
**R0 resection for ICI+CT+A vs. chemo**												
2	randomised trials	not serious	not serious	not serious	serious[a]	none	201/231 (87.0%)	190/233 (81.5%)	**RR 1.08** (0.98 to 1.20)	**65 more per 1,000** (from 16 fewer to 163 more)	⊕⊕⊕◯ Moderate[a]	IMPORTANT
**Grade ≥3 TRAEs during perioperative therapy, ICI+CT+A vs. chemo**												
2	randomised trials	not serious	not serious	serious[c]	not serious	none	119/230 (51.7%)	69/231 (29.9%)	**RR 1.70** (1.29 to 2.25)	**209 more per 1,000** (from 87 more to 373 more)	⊕⊕⊕◯ Moderate[c]	IMPORTANT
**Grade ≥3 TRAEs during neoadjuvant therapy, ICI+CT+A vs. chemo**												

Certainty assessment							№ of patients		Effect		Certainty	Importance
№ of studies	Study design	Risk of bias	Inconsistency	Indirectness	Imprecision	Other considerations	ICI-based neoadjuvant/perioperative strategies	neoadjuvant/perioperative chemotherapy	Relative (95% CI)	Absolute (95% CI)		
2	randomised trials	not serious	not serious	serious[c]	not serious	none	77/230 (33.5%)	44/231 (19.0%)	**RR 1.68** (1.09 to 2.58)	**130 more per 1,000** (from 17 more to 301 more)	⊕⊕⊕◯ Moderate[c]	IMPORTANT

CI: confidence interval; **HR:** hazard ratio; **RR:** risk ratio

Explanations

a. Rated down one level for imprecision due to the wide 95% confidence interval.

b. Rated down one level for inconsistency because substantial heterogeneity was observed across studies.

c. Rated down one level for indirectness because the reported safety observation windows and safety-assessment methods varied across studies.

d. Rated down two levels for very serious imprecision because of sparse events and a very wide 95% confidence interval.

Not downgraded for risk of bias because conference-abstract-derived and high-risk-of-bias studies contributed a limited proportion of the pooled weight, and their exclusion did not materially alter the effect estimates.

eTable 16. Comparison of overlapping systematic reviews and meta-analyses of neoadjuvant/perioperative ICI-based treatment in resectable gastric or gastroesophageal junction cancer

Review	Search sources and cutoff	Question and eligibility criteria	Evidence included	Main findings and conclusions	Relation to the present review
Li 2023 (87)	PubMed, Embase, Cochrane; ASCO, ESMO, ASCO-GI, and ESMO-GI. Jan 1, 2019-Aug 1, 2022; conference abstracts from 2019-2022.	Locally advanced gastric/GEJ cancer receiving neoadjuvant ICI-based therapy. Prospective phase I/II ICI studies were pooled; neoadjuvant chemotherapy studies were synthesized separately as a historical benchmark.	21 prospective ICI studies (687 patients) plus 21 chemotherapy studies (4,800 patients). Regimens included ICI alone, ICI+CT, ICI+CT+A, and ICI+CRT.	Pooled ICI pCR, MPR, and R0 rates were 21%, 41%, and 94%. Efficacy was described as promising; numerical differences among strategies were interpreted from noncomparative data. Mature comparative survival evidence was unavailable.	Broad early synthesis dominated by small single-arm trials and historical comparison. The present review updates the evidence and keeps direct randomized effects separate from descriptive single-arm estimates.
Huang 2024 (88)	PubMed, Embase, Web of Science, Cochrane; ASCO and ESMO. Through Nov 30, 2023. English-language studies.	Locally advanced resectable gastric/GEJ adenocarcinoma. RCTs comparing perioperative ICI-containing therapy plus chemotherapy with chemotherapy alone.	5 RCTs (6 reports; 2,718 patients), including a TKI-containing ICI+CT+A regimen alongside ICI+CT trials.	ICI-containing therapy improved pCR (OR 3.43) but not R0 resection or D2 lymphadenectomy. Overall and grade 3-4 TRAEs were similar, whereas irAEs were increased. Survival was not pooled.	Randomized and comparative, but clinically different strategies were combined and survival data were immature. The present review makes strategy-specific comparisons and incorporates mature OS/EFS evidence.
Wu 2024 (89)	PubMed, Embase, Web of Science, and Cochrane. Search cutoff not reported in the article.	Resectable GEJ cancer receiving neoadjuvant immunotherapy. Heterogeneous immunotherapy combinations were eligible; outcomes were evaluated mainly as single-arm proportions and compared with published	21 studies (753 patients), predominantly small noncomparative cohorts across heterogeneous neoadjuvant immunotherapy regimens.	Pooled pCR, MPR, and ypN0 downstaging rates were 26%, 45%, and 60%; the mean resection rate was 90%. Toxicity was considered acceptable, but long-term effects remained uncertain.	Focused on GEJ cancer and relied mainly on noncomparative estimates and historical ranges. The present review is restricted to G/GEJ adenocarcinoma and distinguishes comparative RCT

		nCT/nCRT ranges.			evidence from prospective single-arm evidence.
Lin 2025 (90)	PubMed and major conference proceedings. Through Aug 15, 2025.	Resectable gastric/GEJ cancer. Randomized phase II/III trials evaluating the addition of an ICI to perioperative chemotherapy.	4 trials (2,358 patients): NEOSUMMIT-01, DANTE, KEYNOTE-585, and MATTERHORN.	ICI+CT improved pCR (RR 2.80), EFS (HR 0.76), and OS (HR 0.78), but not R0 resection. Grade 3-5 irAEs and treatment-related serious AEs were increased.	Strong overlap for perioperative ICI+CT, but no coverage of anti-angiogenic, chemoradiotherapy, HER2-directed, or dual-ICI strategies and no prospective single-arm framework.
Li 2026 (91)	PubMed, Embase, Web of Science, and Cochrane. Through Aug 30, 2025; reference lists screened. English full-text studies only.	Adults undergoing surgery for gastric cancer. Comparative nICT versus nCT studies included RCTs and prospective/retrospective observational cohorts; single-arm nICT studies were added for response, survival, and biomarker analyses.	15 comparative studies plus 34 single-arm or biomarker datasets. Evidence mixed randomized, prospective, retrospective, and observational designs.	nICT improved OS (HR 0.80), RFS (HR 0.78), pathological response, and R0 resection. TRAEs increased, but postoperative outcomes were similar. PD-L1 CPS and dMMR/MSI-H enriched pathological response.	Focused on immunochemotherapy and combined randomized and nonrandomized, including retrospective, evidence. The present review excludes retrospective studies and evaluates five strategies separately.
Zhang 2026 (92)	PubMed, Embase, and CENTRAL through Oct 2024; ASCO, ESMO, and ESMO-GI proceedings from 2023-2024.	Locally advanced resectable gastric/GEJ adenocarcinoma. Phase III RCTs comparing perioperative ICI-containing chemotherapy with chemotherapy alone.	3 phase III RCTs (2,271 patients): KEYNOTE-585, MATTERHORN, and DRAGON IV; the ICI+CT+A DRAGON IV regimen was pooled with ICI+CT trials.	ICI-containing treatment improved pCR (OR 4.04); grade >=3 TRAEs were numerically higher. The authors regarded the approach as promising pending mature OS data.	Restricted to phase III trials and pooled ICI+CT with ICI+CT+A. The present review analyzes these strategies separately and includes updated survival and other prospective strategies.

Ma 2026 (93)	PubMed, Embase, Cochrane, ClinicalTrials.gov, ASCO, ESMO, and CNKI. Through Jan 2025.	Resectable cT3/4aN+M0 EGJ/gastric cancer, including Siewert I-III tumors, treated with ICI+nCRT. RCTs, single-arm studies, and prospective cohorts were eligible; retrospective studies were excluded.	12 studies (406 patients), mostly small single-arm phase I/II trials; sequential/concurrent CRT and single-/dual-ICI regimens were combined. Some cohorts included esophageal tumors.	Pooled pCR, MPR, and R0 rates were 29%, 52%, and 100%; grade >=3 TRAEs occurred in 47%. Two-year PFS and OS were 65% and 74%. Conclusions were explicitly exploratory.	Dedicated ICI+CRT synthesis without an ICI-free comparative effect. The present review places ICI+CRT within a wider framework and reports single-ICI+CRT separately from dual-ICI multimodality treatment.
Pang 2026 (94)	PubMed, Embase, Cochrane Library, Web of Science, Google Scholar, and Research Square; to March 1, 2026.	Evaluated neoadjuvant immunotherapy-based regimens versus chemotherapy in resectable dMMR/MSI-H gastric cancer.	Authors' retrospective cohort plus eight studies (337 patients), including retrospective studies, RCT subgroup analyses, a conference abstract, and an IPD pooled analysis.	Immunotherapy improved pCR, MPR, and EFS, but not OS; pathological-response benefits were not significant after restricting analyses to low-risk-of-bias studies. The evidence was considered preliminary.	Focused exclusively on dMMR/MSI-H disease and mixed retrospective and prospective evidence. Our review covers a broader gastric/GEJ population, includes only RCTs and prospective single-arm trials, and separates evidence by treatment strategy and study design.
